# Efficacy of flavonoids in non-alcoholic fatty liver disease: an updated systematic review and meta-analysis

**DOI:** 10.3389/fnut.2025.1660065

**Published:** 2025-09-15

**Authors:** Qianqian Liu, Haodi Luan, Zhijiao Duan, Jing Ai, Yan Wang, Ping Chen

**Affiliations:** ^1^Department of Gastroenterology, The Affiliated Hospital of Inner Mongolia Medical University, Hohhot, China; ^2^Clinical Nutrition Department, The Affiliated Hospital of Inner Mongolia Medical University, Hohhot, China; ^3^Critical Care Medicine Department, The Affiliated Hospital of Inner Mongolia Medical University, Hohhot, China; ^4^Health Examination Center, Hohhot Traditional Chinese and Mongolian Medical Hospital, Hohhot, China

**Keywords:** non-alcoholic fatty liver disease (NAFLD), flavonoids, liver enzymes, insulin resistance, meta-analysis

## Abstract

**Background:**

Non-alcoholic fatty liver disease (NAFLD) is a common health challenge worldwide and urgently requires effective therapeutic interventions. Flavonoids, a diverse group of plant-derived polyphenols, have demonstrated multifaceted biological functions, like anti-inflammatory, antioxidant and metabolic regulatory capacities, suggesting their potential utility in the management of NAFLD.

**Objective:**

This systematic review and meta-analysis aims to synthesize evidence from randomized controlled trials (RCTs) that examined the effects of flavonoid supplementation (including specific subclasses) in patients with NAFLD on liver function, markers of inflammation, lipid profiles, anthropometric measures and insulin resistance.

**Methods:**

This study rigorously adhered to the Cochrane Handbook for Systematic Reviews of Interventions (Version 6.5, 2024) and the PRISMA guidelines. A substantial literature review was obtained from PubMed, OVID, Web of Science Core Collection, Embase, and Cochrane Library databases, including studies published through December 2024.

**Results:**

The analysis incorporated 25 RCTs involving 1,689 participants with NAFLD. Flavonoids intervention significantly decreased aspartate aminotransferase (AST), alkaline phosphatase (ALP), alanine aminotransferase (ALT), total cholesterol (TC), steatosis score, body mass index (BMI), triglycerides (TG), fasting blood sugar (FBS), insulin levels and augmented the quantitative insulin sensitivity check index (QUICKI) levels. However, no significant alterations were observed in gamma-glutamyl transferase (GGT), low-density lipoprotein cholesterol (LDL-C), high-density lipoprotein cholesterol (HDL-C), body weight (WT), waist-to-height ratio (WHtR), hip circumference (HC), Waist Circumference (WC). Moreover, the impact on hepatic steatosis and fibrosis scores was non-significant.

**Conclusion:**

Flavonoids exhibit potential therapeutic benefits in mitigating liver enzyme levels, lipid profiles, enhancing insulin sensitivity among NAFLD patients. Nonetheless, their influence on inflammatory markers, and fibrosis scores appears to be limited. Future investigations should focus on assessing the long-term security and effectiveness of flavonoid supplementation in managing NAFLD and exploring their synergistic potential in combination with other therapeutic strategies.

**Systematic review registration:**

https://www.crd.york.ac.uk/PROSPERO/view/CRD420251001203.

## 1 Introduction

NAFLD is a spectrum disorder defined by ≥5% hepatic macrovesicular steatosis after excluding secondary etiologies (e.g., drugs, monogenic disorders) and excessive alcohol use. NAFLD can progress from simple steatosis to more severe conditions such as non-alcoholic steatohepatitis (NASH), fibrosis, cirrhosis and eventually hepatocellular carcinoma (HCC) (1). Since June 2023, NAFLD has been redefined as metabolic dysfunction-associated steatotic liver disease (MASLD), which emphasizes the importance of metabolic dysfunction in the pathogenesis (2). Global epidemiological trends reveal expanding overweight/obesity rates, driven by significant shifts in diet and lifestyle. This surge correlates with rising prevalence of chronic illnesses associated with obesity, including cardiovascular disease (CVD), metabolic syndrome, type 2 diabetes (T2DM) and NAFLD (3, 4). Latest evidence from the Global Burden of Disease (GBD) study reveals a concerning escalation in the prevalence of NAFLD, with the global number of affected patients doubling over the three-decade period from 1990 to 2021 (5). NAFLD affects 30.05% of the population worldwide, and it is predicted that 33.5% of adults aged ≥15 years will have NAFLD by 2030 (6, 7).

NAFLD is a clinically diverse disease influenced by environment, diet, and genetics, with its specific pathogenesis still unclear. NAFLD pathogenesis is classically attributed to the “two-hit” hypothesis: First hit – Adipose insulin resistance drives lipolysis and free fatty acid flux, overwhelming hepatic uptake to cause steatosis; Second hit – Prolonged lipid toxicity induces ER stress, mitochondrial damage, and oxidative injury, triggering inflammation and progression to NASH. Adipose dysfunction (reduced adiponectin, pro-inflammatory cytokines) further fuels insulin resistance and inflammation in a self-amplifying loop (8).

NAFLD/NASH currently lacks approved pharmacotherapies, with clinical management primarily focused on lifestyle modifications such as Mediterranean diets rich in Flavonoids (9). Flavonoids—a structurally diverse class of plant-derived phenolic compounds—show particular promise. Flavonoids demonstrate multi-target bioactivities by modulating a range of signaling pathways. Their antioxidant activity involves direct free radical scavenging, chelation of metal ions (e.g., Fe^2+^, Cu^2+^), and enhancement of endogenous antioxidant enzymes (e.g., SOD, CAT, GPx) through the Nrf2/ARE pathway. Furthermore, flavonoids support cardiovascular health by improving lipid metabolism—notably reducing ox-LDL and triglycerides—promoting endothelial NO release, and suppressing platelet activation. They also exhibit anti-diabetic effects via stimulation of insulin secretion, activation of insulin signaling pathways (including PI3K/Akt and AMPK), and inhibition of gluconeogenic enzymes such as PEPCK and G-6-Pase (10). Flavonoids, due to these characteristics, have shown promise in clinical interventions for multiple metabolic disorders, such as CVD, NAFLD and neurodegenerative diseases (11–13)_._

Over 5,000 identified flavonoids (classified into 7 subgroups: flavones, isoflavones, flavanols, etc.) demonstrate multi-target effects in NAFLD through antioxidation, anti-inflammation, and metabolic regulation properties (14, 15). Despite preclinical evidence supporting flavonoids’ multi-target effects in NAFLD, clinical translation remains limited by inconsistent efficacy reports and mechanistic ambiguity across trials, particularly regarding their impacts on metabolic parameters. The heterogeneity may partially stem from variations in flavonoid subtypes and dosage regimens. Existing meta-analyses may not encompass the latest clinical trials, particularly involving novel formulations. By integrating the most recent data, this systematic review and meta-analysis provides an updated and comprehensive efficacy assessment, and synthesizes RCTs to delineate subclass-specific benefits, dose–response relationships, and biomarker correlations, providing evidence-based guidance for integrating flavonoids into precision nutrition strategies against NAFLD.

## 2 Materials and methods

To ensure methodological rigor and transparency, this systematic review and meta-analysis strictly followed the Cochrane Handbook for Systematic Reviews of Interventions (Version 6.5, 2024) (16) and adhered to the Preferred Reporting Items for Systematic Reviews and Meta-Analyses (PRISMA) guidelines (17). The protocol of the study has been prospectively submitted to the PROSPERO international registry (Registration ID: CRD420251001203).

### 2.1 Search strategy and selection criteria

A systematic search of the literature was carried out in 5 databases (PubMed, OVID, Web of Science Core Collection, Embase, Cochrane Library) to identify studies published before December 2024. The search strategy incorporated both Medical Subject Headings (MeSH) terms and keywords associated with NAFLD, flavonoids, and therapeutic interventions. The selection criteria were limited by language and publication type (Appendix A1). Furthermore, a manual examination of reference lists from both included studies and pertinent review articles was undertaken to locate additional qualifying literature.

### 2.2 Inclusion and exclusion criteria

Two independent reviewers (Qianqian Liu and Haodi Luan) employed the PICOS framework to filter articles that examined the efficacy of flavonoid supplements on health outcomes. The inclusion criteria were: (1) Adult patients diagnosed with NAFLD; (2) Flavonoids supplementation for at least 4 weeks; (3) Presence of a placebo or control group for comparison; (4) Only randomized controlled trials (RCTs) were included. (5) Studies published in English. Trials were excluded if they failed to meet the following criteria: (1) review articles, case reports, conference proceedings, non-randomized controlled trials, observational studies, and animal studies; (2) lack of data availability; (3) did not report on the specified outcome measures of interest. Inconsistencies were reconciled by consensus among the reviewers or, if necessary, in consultation with a third reviewer (Zhijiao Duan).

### 2.3 Study selection and data extraction

The study implemented a systematic literature screening and data extraction protocol: two reviewers (Haodi Luan and Zhijiao Duan) conducted dual-blind title/abstract screening, resolving discrepancies through consensus discussions with third-reviewer arbitration (Qianqian Liu) when needed, followed by full-text assessments. Independent parallel data extraction by trained researchers (Haodi Luan and Zhijiao Duan) utilized a standardized form capturing study design, demographics, intervention parameters, outcome metrics, and quantitative data (pre/post means ± SDs), supplemented by tripartite verification—third-researcher validation and author communications with customized data templates.

### 2.4 Risk of bias assessment

The quality of the included studies was assessed using the Cochrane Risk of Bias Tool. Two reviewers (Jing Ai and Yan Wang) independently assessed the risk of bias. Divergent assessments underwent structured group deliberation with all research team members to establish consensus-based ratings, ensuring inter-rater reliability and minimizing subjective interpretation errors.

### 2.5 Statistical analysis

The Statistical analyses were performed using STATA, version 16(Stata Corp, College Station, TX). To assess the effects of flavonoid supplementation on health outcomes, we calculated the standard deviations (SD) and mean differences between the intervention and control groups. These data were then used to determine the standardized mean difference (SMD) with 95% confidence intervals (CIs), applying a random-effects model for analysis. A random-effects model was applied for all meta-analyses, as we anticipated substantial clinical and methodological heterogeneity across studies due to variations in flavonoid subtypes, dosages, intervention durations, and participant characteristics. This model provides a more conservative estimate of the effect size and is appropriate when studies are not functionally identical, allowing for generalization beyond the included studies. Heterogeneity among the included studies was evaluated using *I*^2^ statistics, the interpretation of *I*^2^ values was as follows: 0% indicated no heterogeneity, <25% represented mild heterogeneity, 25–50% suggested moderate heterogeneity, and >50% reflected substantial heterogeneity across studies (18). To further investigate potential influencing factors, we conducted subgroup analyses based on key study characteristics, such as flavonoids type, flavonoids dose.

To evaluate potential publication bias, we conducted funnel plot visualization and quantified asymmetry using Egger’s regression test (Appendix A2). To address missing data, we used multiple imputation techniques to estimate missing values, ensuring robustness in our findings. To assess result stability, we conducted sensitivity analyses that systematically excluded studies with high risk of bias and re-analyzed the data (Appendix A2). All statistical analyses were performed using two-sided tests, with a significance threshold set at *p* < 0.05.

## 3 Results

### 3.1 Search results

**Figure 1:**
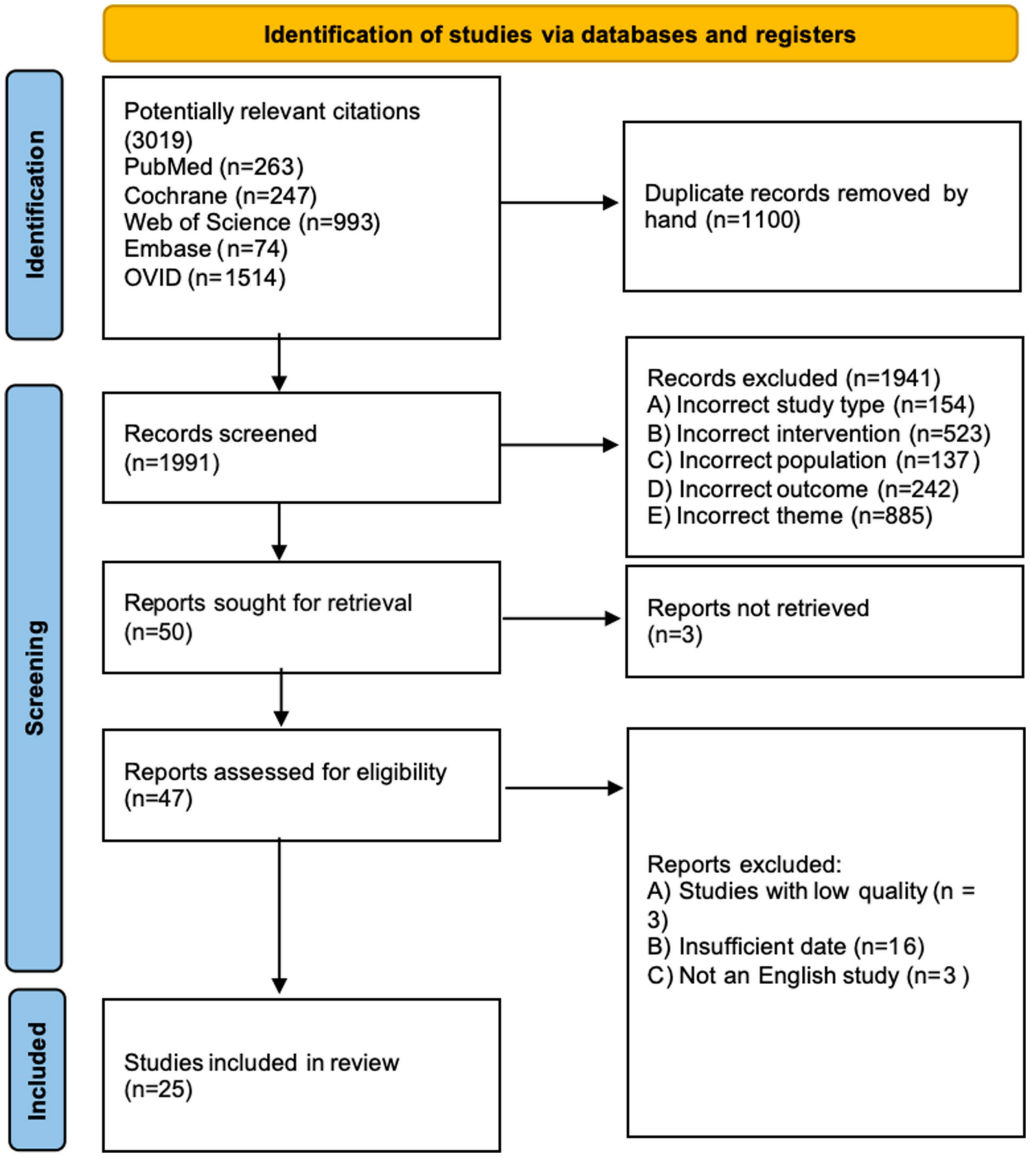
Prisma flow diagram of study selection process.

Our literature search initially yielded 3,019 publications (see attached document for the detailed search strategy). Following duplicate removal, we screened 1991 records based on their titles and abstracts. Of these, 47 articles underwent full-text assessment for eligibility. After careful evaluation, our review and meta-analysis incorporated 25 eligible studies. The PRISMA flow diagram provides a comprehensive overview of the exclusion process and reasons for article exclusion at each stage (Figure 1).

### 3.2 Characteristics of included studies

This meta-analysis included 25 RCTs investigating the effects of nine flavonoid—quercetin, genistein, naringenin, hesperidin, anthocyanin, pueraria, isoflavones, catechin, and silymarin—on patients with NAFLD or NASH. Flavonoid dosages varied from 32 to 1,080.6 mg per day, with intervention periods ranging from 4 to 24 weeks, and the most frequent duration was 12 weeks (*n* = 10). The RCTs were conducted across multiple countries, including Iran (*n* = 17), China (*n* = 3), Pakistan (*n* = 3), Turkey (*n* = 1), Spain (*n* = 1). Detailed study characteristics are presented in Table 1.

**Table 1:** Characteristics of all trials included in the present meta-analysis.

Flavonoids	Author	Design	Country	Population (IG/CG)	Mean age			Gender (M/F)		Intervention		Duration	Relevant outcomes
					IG	CG		IG	CG	IG	CG		
Quercetin	Li et al.2024 (35)	Double-BlindRCT, Crossover	China	41/41	39.2 ± 11.7	40.1 ± 9.1	I:27/14	C:27/14	Quercetin 500 mg	Placebo	12 weeks	WT, HbA1c, ALT TG, LDL-C	
	Hosseinikia et al.2020 (36)	Double-Blind RCT, Parallel	Iran	39/39	43.4 ± 11.1	45.9 ± 9.2	I:15/24	C:13/26	Quercetin 500 mg	Placebo	12 weeks	TNF-α, GGT, TG, TC LDL, HDL, BMI, Hs-CRP, WHR	
	Pasdar et al.2020 (37)	Double-Blind RCT, Pilot	Iran	39/39	43.46 ± 11.13	45.89 ± 9.16	I:15/24	C:13/26	Quercetin 500 mg	Placebo	12 weeks	RBC, MCHC	
Genistein	Amanat et al.2018 (38)	Double-Blind RCT, Parallel	Iran	41/41	44.22 ± 11.80	42.94 ± 9.55	I:30/11	C:31/10	Genistein 250 mg	Placebo	8 weeks	FBS, HOMA-IR, IL-6, TNF-α WHR, TG, BMI, LDL-C, HDL-C, AST, ALT	
Naringenin	Namkhah et al.2021 (39)	Double-Blind RCT, Parallel	Iran	22/22	44.7 ± 10.7	47 ± 9.4	I:12/10	C:13/9	Naringenin 200 mg	Placebo	4 weeks	NFS, WT, BMI, TG, TC, LDL, HDL, WC	
Hesperidin	Yari et al.2020 (40)	Open-labeled Parallel	Iran	22/21	45.82 ± 11.69	46.11 ± 11.63	I:11/11	C:10/11	Hesperidin1,000 mg + Lifestyle modification	Lifestyle modification	12 weeks	HOMA-IR, CRP, TNF-α, NF-κB, Fibrosis score、Steatosis score FLI	
Anthocyanin	Sangsefidi et al. 2021 (41)	Double-Blind RCT, Parallel	Iran	25/25	41.48 ± 9.53	42.68 ± 9.96	I:12/13	C:11/14	Anthocyanin 32 mg	Placebo	12 weeks	ALT, AST, CK-18, Fibrosis score, Steatosis score	
	Yarhosseini et al. 2021 (42)	Double-Blind RCT, Parallel	Iran	25/25	41.4 ± 9.5	42.6 ± 9.9	I:12/13	C:11/14	Anthocyanin 32 mg	Placebo	12 weeks	WC, HC, WHR	
	Izadi et al. 2020 (43)	Double-Blind RCT, Parallel	Iran	30/31	43.3 ± 10.2	42.8 ± 10.6	I:17/13	C:19/12	Anthocyanin 250 mg	Placebo	8 weeks	TG, ALT, AST, TC, LDL-C, HDL-C, WT, BMI, WC	
	Zhang et al. 2014 (44)	Double-Blind RCT, Parallel	China	37/37	44.9 ± 7.5	46.9 ± 7.7	I:19/18	C:20/17	Anthocyanin 320 mg	Placebo	12 weeks	ALT, AST, TG, TC, LDL, HDL-C, BMI, CK-18, HOMA-IR, WHR	
	Bayram et al. 2024 (45)	RCT, Parallel	Turkey	22/22	43.90 ± 10.44	43.40 ± 12.46	I:10/12	C:10/12	Anthocyanin 350 mg	Placebo	8 weeks	ALT, AST, ALP, GGT, TG, TC, LDL, HDL, FBG, HbA1c, HOMA-IR, CRP, BMI, WC, HC	
	Ghanbari et al. 2024 (46)	Double-Blind RCT, Parallel	Iran	25/25	43.52 ± 8.12	44.88 ± 10.14	I:10/15	C:14/11	Grape Seed Extract 520 mg	Placebo	8 weeks	ALT, AST, TG, TC, LDL-c, HDL-c, BMI, QUICK, HOMA-IR	
Pueraria	Li et al. 2024 (47)	Triple-blind RCT, Parallel	China	60/61	56.2 ± 10	58.1 ± 9.6	I:30/30	C:31/30	Silybin 138.0 mg, Puerarin 68.4 mg, Salvianolic acid 65.4 mg	Placebo	24 weeks	FIB-4, HOMA-IR, WT, BMI, WC, WHR, CRP, IL-6, FIB-4, APRI, NFS	
Isoflavones	Tehrani et al. 2024 (48)	Double-Blind RCT, Parallel	Iran	25/21	F:51.93 ± 11.15, M:47.60 ± 14.98	F:52.09 ± 5.73 M:46.0 ± 14.10	I:10/15	C:10/11	Soy isoflavones 100 mg	Placebo	12 weeks	ALT, AST, Steatosis score, Fibrosis score, GGT, FGF-21, WT, BMI, WC, WHR	
Catechin	Pezeshki et al. 2016 (49)	Double-Blind RCT, Parallel	Iran	35/36	None	None	I:16/19	C:16/20	Catechins 3.45 mg Caffeine 11.375 mg, GC:25.66 mg, EGC:15.38 mg, EC:29.305 mg, EGCG:157.145 mg, GCG:11.45 mg, ECG:13.235 mg	Placebo	90 days	AST, ALT, ALP, WT, BMI	
Silymarin	Hashemi et al. 2009 (50)	Double-Blind RCT, Parallel	Iran	50/50	39.28 ± 11.117	39.0 ± 10.70	I:28/22	C:29/21	Silymarin 280 mg	Placebo	24 weeks	AST, ALT, FBS, TG, TC, LDL, HDL, BMI	
	Solhi et al.2014 (51)	RCT, Parallel	Iran	33/31	43.6 ± 8.3	39.36 ± 10.5	I:19/14	C:19/12	Silymarin 210 mg	Placebo	8 weeks	AST, ALT	
	Anushiravani et al. 2019 (52)	Double-Blind RCT, Parallel	Iran	30/30	None	None	None	None	Silymarin 140 mg	Placebo	12 weeks	BMI, WC, TG, TC, LDL-C, HDL-C, FBS	
	Aller et al. 2015 (53)	RCT, Parallel	Spain	18/18	None	None	None	None	Silymarin 1080.6 mg + Vitamin E 72 mg + Lifestyle modification	Lifestyle modification	12 weeks	BMI, WC, GGT, FLI, NFS, ALT, AST, TG, FBS, HOMA-IR, WT	
	Memon et al. 2015 (54)	Double-Blind RCT, Parallel	Pakistan	31/33	49.0 ± 9.70	48 ± 8.9	I:21/12	C:21/10	Silymarin 280 mg	Placebo	3 months	ALT, AST, TG, TC, LDL, HDL	
	Rangboo et al. 2016 (55)	Double-Blind RCT, Parallel	Iran	30/30	47.27 ± 8.12	49.83 ± 12.79	I:21/9	C:21/9	2,700 mg *Cynara scolymus* extract	Placebo	2 months	ALT, AST, TG, TC, LDL-C, HDL-C, FBS	
	Shaikh et al. 2021 (56)	RCT, Parallel	Pakistan	100/100	None	None	None	None	Silymarin 400 mg	Placebo	12 weeks	AST, ALT	
	Mirhashemi et al. 2022 (57)	Double-Blind RCT, Parallel	Iran	27/25	37.81 ± 9.93	38.08 ± 10.01	None	None	560 mg Silymarin+Lifestyle modification	Placebo +Lifestyle modification	8 weeks	AST, ALT, AST/ALT, BMI, Fib-4, NFS	
	Atarodi et al. 2022 (58)	Double-Blind RCT, Parallel	Iran	27/29	36.46 ± 10.00	37.52 ± 8.94	I:9/18	C:8/21	Silymarin 140 mg	Placebo	4 weeks	ALT, AST, ALP, TB, DB, TG, TC, LDL-C, HDL-C, BMI	
	Hafiza et al. 2024 (59)	RCT, Parallel	Pakistan	I1:16 I2:16 I3:16 C:16	None	None		I1:7/9 I2:6/10 I3:8/8	C:8/8	I1: Silymarin 200 mg I2: Silymarin 300 mg I3: Silymarin 400 mg	Placebo	3 months	ALT, AST, ALP, CRP, ESR, BMI

AST, Aspartate Aminotransferase; ALT, Alanine Aminotransferase; ALP, Alkaline Phosphatase; GGT, G-Glutamyl-Transferase, BMI, Body Mass Index; WT, Weight; HC, Hip Circumference; WC, Waist Circumference; WHR, Waist-to-Hip Ratio; CRP; C-Reactive Protein; TNF-α:tumor necrosis factor; IL-6, Interleukin-6; CK-18, Cytokeratin-18; LDL-C, Low-Density lipo-Protein Cholesterol; TC, Total Cholesterol; HDL-C: High-Density Lipoprotein Cholesterol; TG, Triglycerides; FBS, Fasting Blood Sugar; HOMA-IR, Homeostatic Model Assessment of Insulin Resistance; QUICKI, Quantitative Insulin Sensitivity Check Index; IG, Intervention group; CG, Control group; I, Intervention; C, Control.

### 3.3 Risk of bias assessment

**Figure 2:**
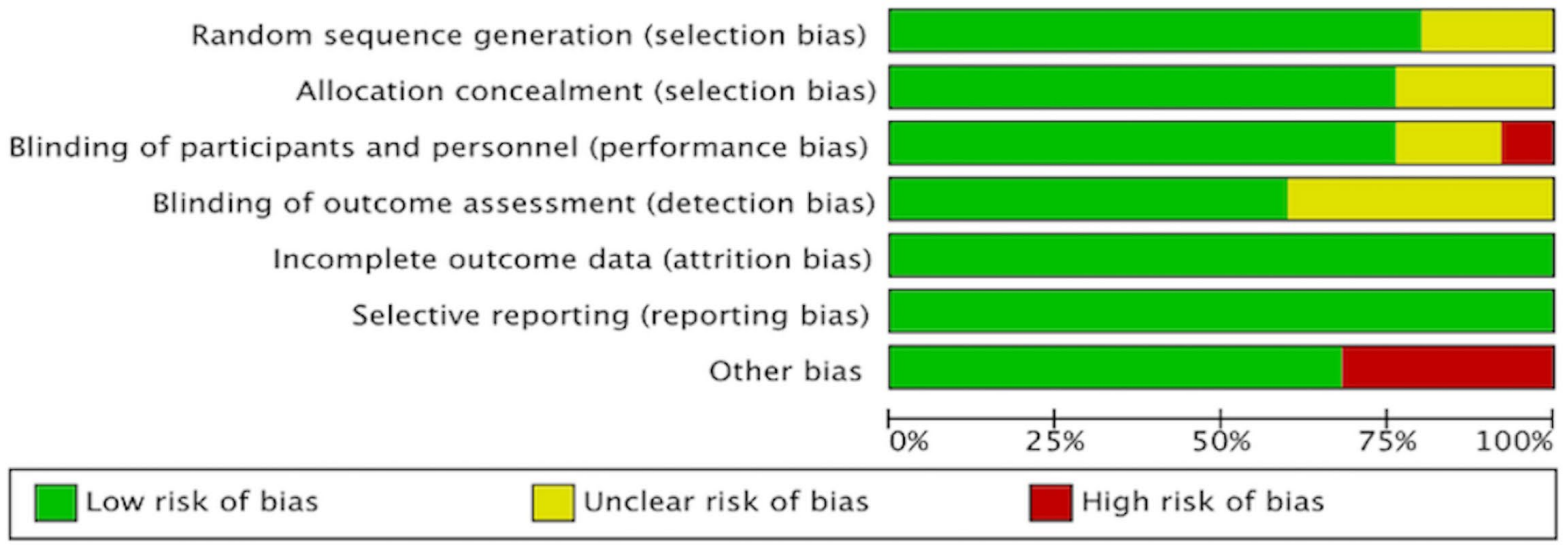
Risk of bias assessment of the included studies.

**Figure 3:**
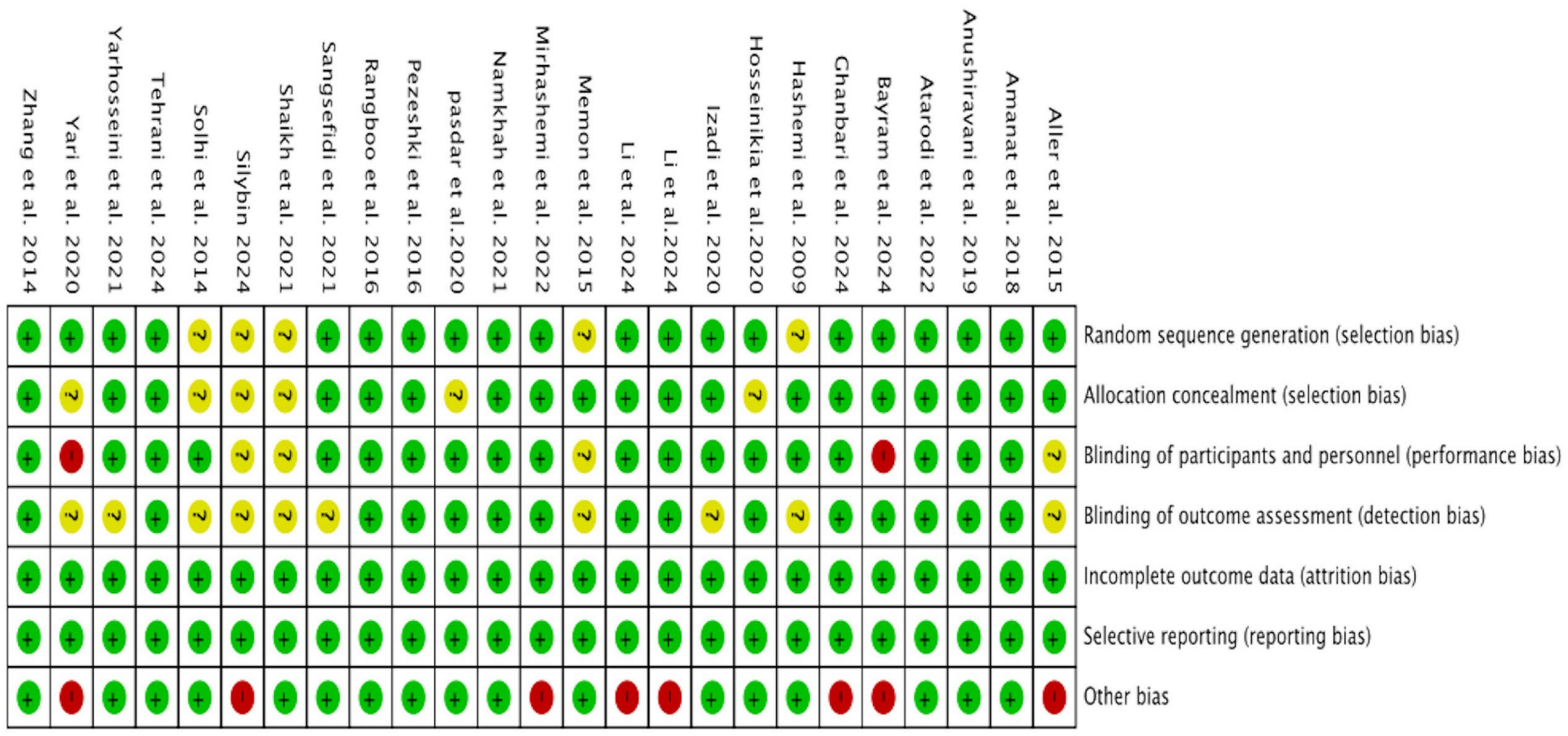
Risk of bias assessment of the included studies.

We assessed the risk of bias in all RCTs using the Review Manager, with a visual summary and graphical representation provided in Figures 2, 3. This systematic review included a total of 25 randomized controlled trials (RCTs). The overall risk of bias, as assessed by the Cochrane Risk of Bias tool, was low. Regarding specific domains: for randomization, 20 trials were rated as low risk and 5 as unclear; for allocation concealment, 19 were low risk and 6 were unclear. Blinding of participants and personnel was low risk in 19, high risk in 2, and unclear in 4 trials. Blinding of outcome assessment was low risk in 15 and unclear in 10 trials. The risks of bias from incomplete outcome data and selective reporting were low for the majority of included studies. The risk of other biases was high in 8 and low in 17 studies.

### 3.4 Liver function effects of flavonoid supplementation

**Figure 4:**
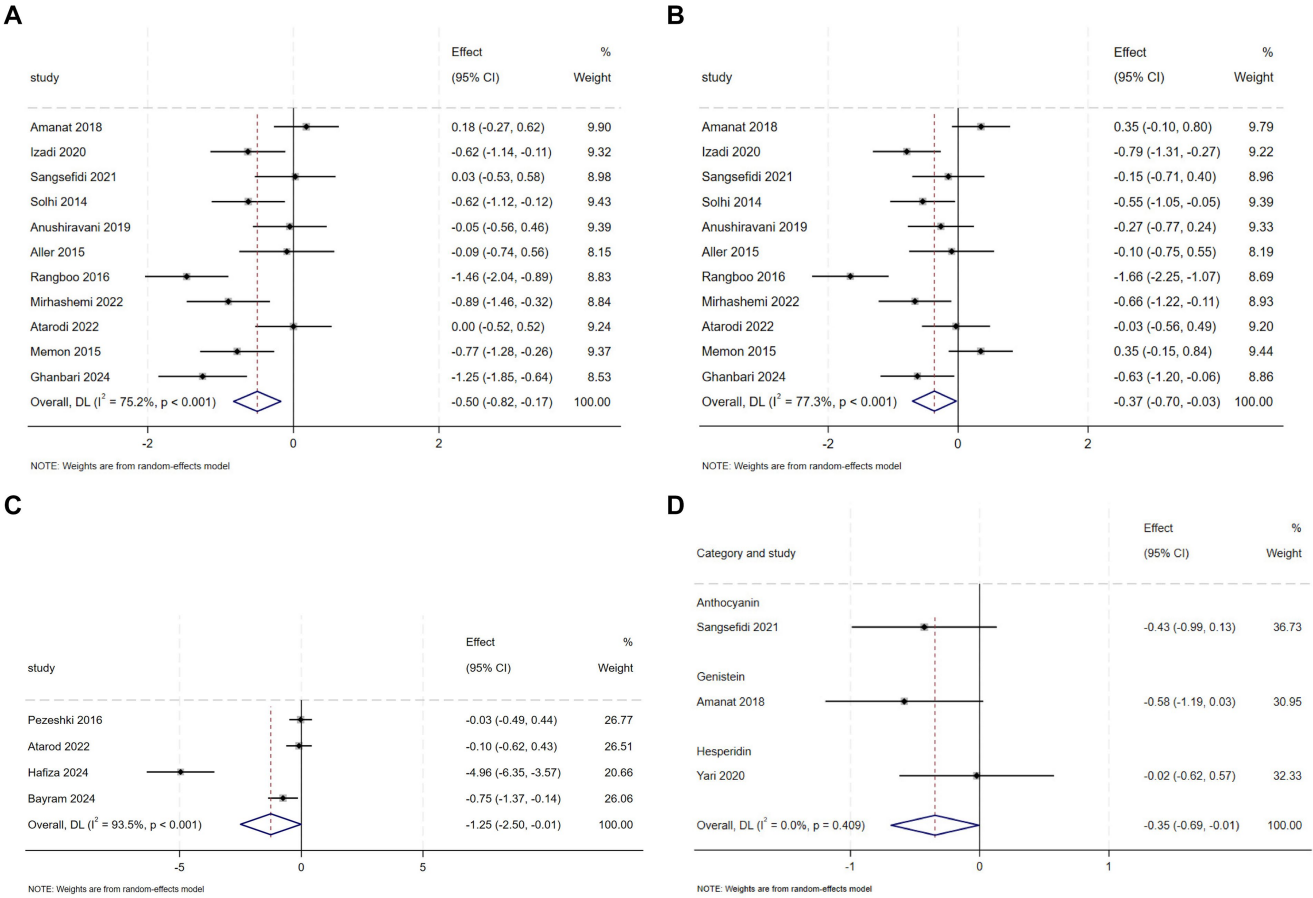
**(A)** Effect of flavonoid on ALT **(B)** Effect of flavonoid on AST **(C)** Effect of flavonoid on ALP **(D)** Effect of flavonoid on Steatosis score.

Totally, 11 RCTs evaluated flavonoid supplementation on ALT and AST in 631 NAFLD patients. Respectively, For ALT, pooled data from the trials demonstrated that flavonoids intervention reduced ALT levels in comparison with the control group (SMD = −0.50, 95% CI: −0.82 to −0.17, *p* = 0.003), with substantial heterogeneity (*I*^2^ = 75.20%, *p* < 0.001) (Figure 4A). In a subset analysis, silymarin significantly reduced ALT concentrations (Appendix A3, Figure 1.1) and silybin were effective in reducing ALT concentrations at doses of ≥2000 mg (Appendix A3, Figure 1.3). A pronounced reduction in ALT levels was observed with anthocyanin intervention within a period of fewer than 12 weeks (Appendix A3, Figure 1.2). For AST, the intervention group exhibited a decrease in levels compared to the control group (SMD = −0.37, 95% CI: −0.70 to −0.03, *p* = 0.034), with substantial heterogeneity (*I*^2^ = 77.3%, *p* < 0.001) (Figure 4B). The efficacy of anthocyanin in reducing AST levels was noted in subgroup analysis (Appendix A3, Figure 2.1). Similarly, silymarin reduced AST levels at higher dosage thresholds of ≥2000 mg (Appendix A3, Figure 2.2).

Meta-analysis of 4 RCTs showed that flavonoid intervention markedly reduced ALP levels compared to placebo (SMD = −1.25, 95% CI: −2.50 to −0.01, *p* = 0.048), however, there was considerable heterogeneity between the studies (*I*^2^ = 93.5%, *p* < 0.001) (Figure 4C). Subgroup analyses uncovered that specific flavonoid subtype—namely anthocyanin was effective in lowering ALP levels (Appendix A3, Figure 3.1). Geographical stratification showed significant reductions in studies conducted in Pakistan and Turkey compared to other regions (Appendix A3, Figure 3.2).

**Figure 5:**
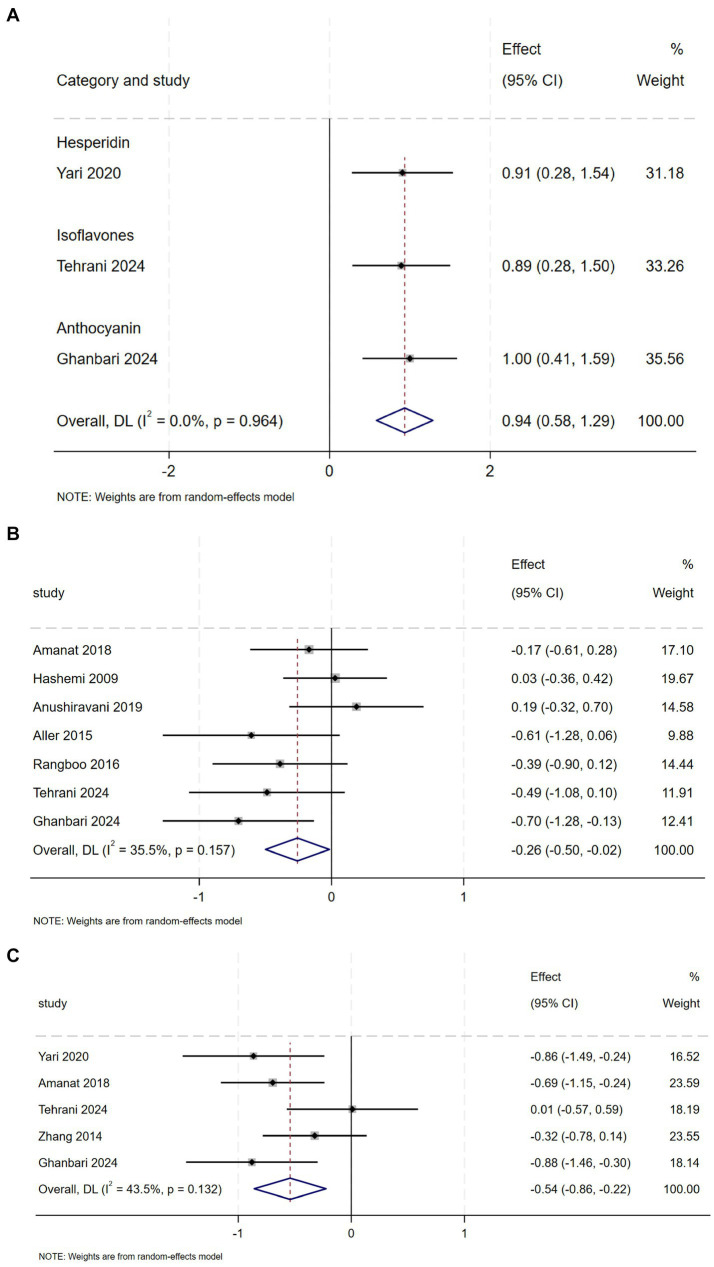
**(A)** Effect of flavonoid on QUICKI **(B)** Effect of flavonoid on FBS **(C)** Effect of flavonoid on Insulin.

Pooled RCT analysis revealed no statistically meaningful reduction in GGT levels following flavonoids intervention compared to controls (SMD = −0.37, 95% CI: −0.89 to 0.16, *p* = 0.173), with high heterogeneity between studies (*I*^2^ = 60.7%, *p* = 0.079) (Appendix A4, Figure 1). Finally, when pooled data from these studies were analysed, there were no significant differences in hepatic steatosis and fibrosis between placebo and intervention groups (Appendix A4, Figures 2–5), with the exception of the Steatosis score (Figure 4D).

### 3.5 Effects on FBS and insulin resistance with flavonoid supplementation

Compared to the placebo group, the intervention group showed a perceptible decline in FBS, as indicated by comprehensive analysis (SMD = −0.26, 95% CI: −0.50 to −0.02, *p* = 0.037; 1^2^ = 35.5%, *p* = 0.157) (Figure 5B) and Insulin (SMD = −0.54, 95% CI: −0.86 to −0.22, *p* = 0.001; 1^2^ = 43.5%, *p* = 0.132) (Figure 5C), with moderate heterogeneity observed in both analyses. Subgroup analyses demonstrated that anthocyanin supplementation exerted a more pronounced effect on FBS reduction, whereas hesperidin, genistein, and anthocyanins decreased insulin concentrations (Appendix A3, Figures 9, 10).

**Figure 6:**
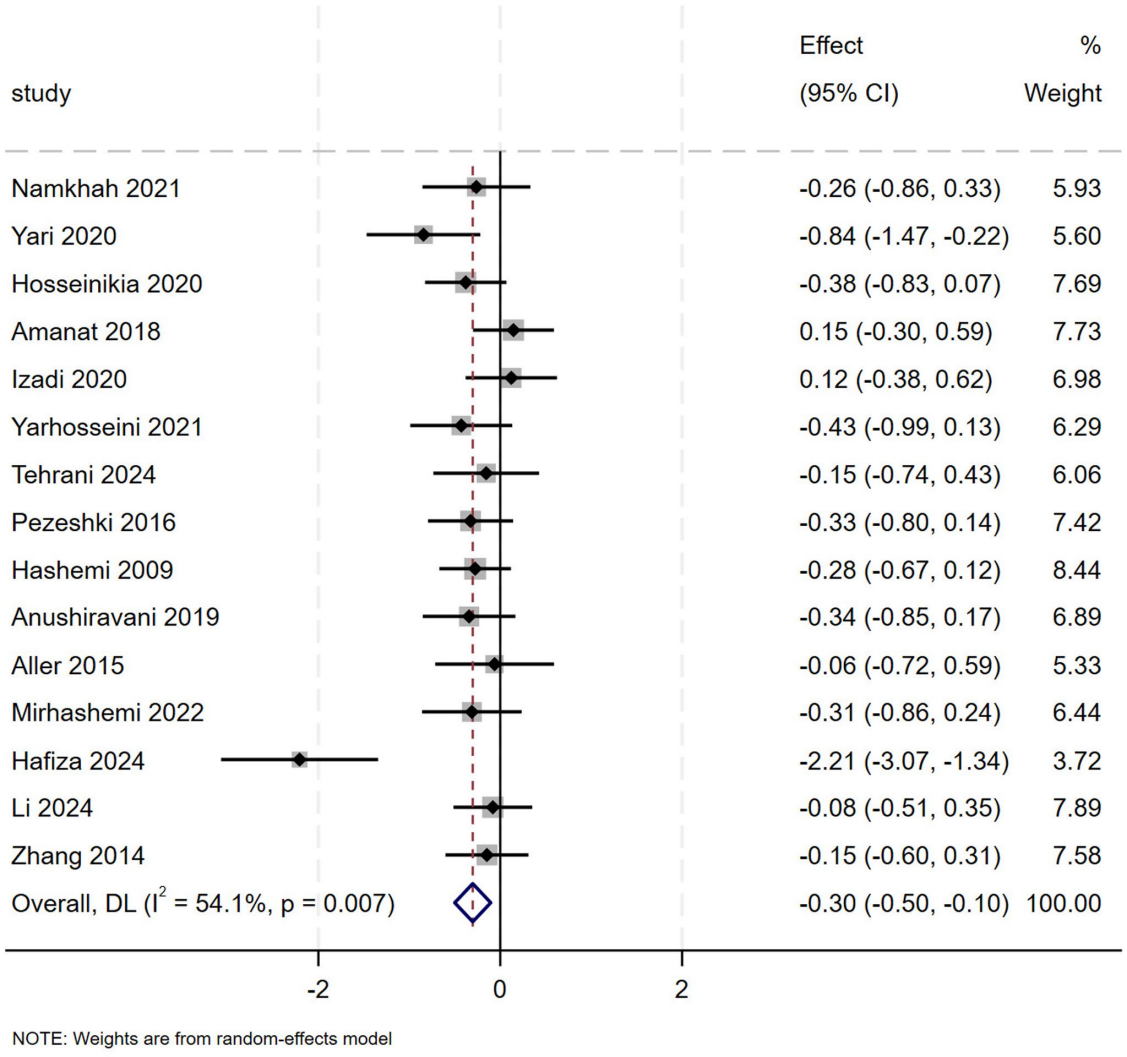
Effect of flavonoid on BMI.

And compared with the control group, QUICKI values were higher in the intervention group (SMD = 0.94, 95% CI: 0.58 to 1.29, *p* = 0.00), with Mild heterogeneity (1^2^ = 0.0%, *p* = 0.964) (Figure 5A). However, pooled analysis of RCTs revealed no statistically significant reduction in HOMA-IR level following flavonoids intervention compared to control (Appendix A4, Figure 6).

### 3.6 Effects on anthropometric indices with flavonoid supplementation

**Figure 7:**
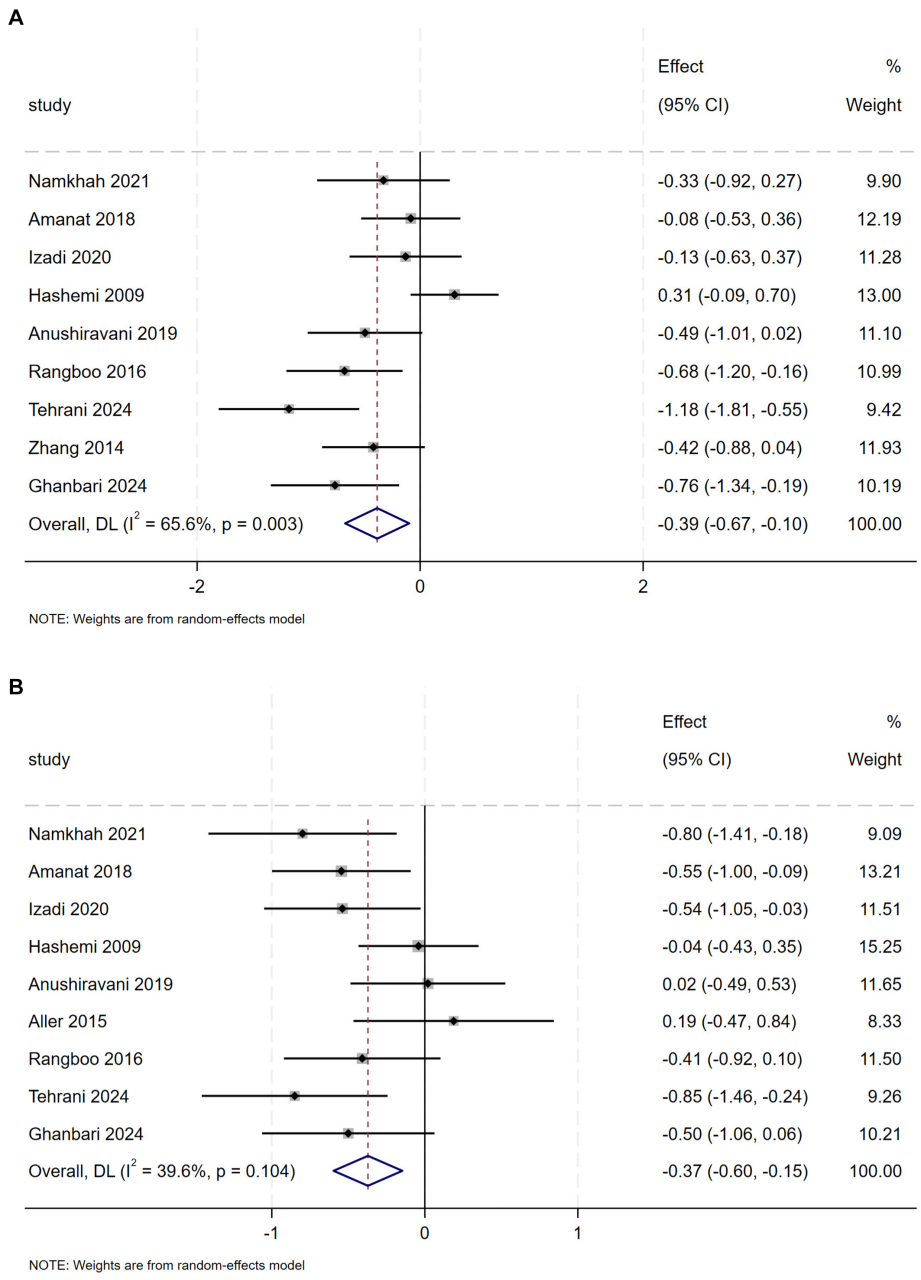
**(A)** Effect of flavonoid on TC **(B)** Effect of flavonoid on TG.

Pooled data from 15 clinical trials (909 NAFLD patients) showed that flavonoids led to a more appreciable decrease in BMI compared to controls (SMD = −0.30, 95% CI: −0.50 to −0.10, *p* = 0.003), with moderate heterogeneity (*I*^2^ = 54.1%, *p* = 0.007) (Figure 6). Hesperidin was associated with reduced BMI in subgroup analyses (Appendix A3, Figure 5.1). Geographical stratification showed significant reductions in studies conducted in Pakistan and Iran compared to Spain (Appendix A3, Figure 5.2). In the evaluation of HC (3 studies, *n* = 178), combined results indicated no statistically significant effect of flavonoids versus placebo (SMD = −0.22, 95% CI: −0.58 to 0.13, *p* = 0.218), with substantial heterogeneity (*I*^2^ = 28.8%, *p* = 0.245) (Appendix A4, Figure 7).

Comparing flavonoids supplementation with placebo in 10 studies revealed no meaningful change in WC (SMD = −0.06, 95% CI: −0.22 to 0.10, *p* = 0.471), with mild heterogeneity (*I*^2^ = 0.0%, *p* = 0.515) (Appendix A4, Figure 8). However, subgroup analysis revealed that hesperidin reduced WC concentration (Appendix A3, Figure 6). Meta-analysis of available trials (5 for WHtR, 10 for WT) demonstrated that flavonoid intervention had no statistically significant impact on WHtR (Appendix A4, Figure 9) and WT (Appendix A4, Figure 10). And subgroup analyses showed no significant differences based on type or dose categories. (Appendix A3, Figures 7, 8).

### 3.7 Effects on inflammatory markers with flavonoid supplementation

Totally, 2, 1, 2, and 1 studies evaluated the efficacy of flavonoids on Hs-CRP, IL-6, TNF-a, and CK-18, respectively. Pooling data revealed that flavonoids supplementation showed no significant change in inflammatory markers (Appendix A4, Figure 11). However, subgroup analysis revealed that genistein significantly reduced inflammatory markers (Appendix A3, Figure 11.1). And flavonoids supplementation showed significant change in IL-6 (Appendix A3, Figure 11.2).

### 3.8 Effects on lipid profile with flavonoid supplementation

Comparing flavonoid supplementation with placebo in 9 studies indicated no substantial change in LDL (SMD = −0.28, 95% CI: −0.57 to 0.01, *p* = 0.059), with substantial heterogeneity (*I*^2^ = 65.9%, *p* = 0.003) (Appendix A4, Figure 12). However, subgroup analysis revealed that anthocyanin and isoflavones and decrease LDL concentrations (Appendix A3, Figure 15.1). In our meta-analysis of 7 trials (*n* = 463 NAFLD patients), flavonoid intervention demonstrated no significant effect on HDL levels (Appendix A4, Figure 13). And subgroup analyses showed no significant differences based on type or dose categories (Appendix A3, Figure 14).

TC was evaluated in 9 studies with 573 NAFLD patients. The meta-analysis revealed a statistically significant reduction in TC (SMD = 0.39, 95% CI: −0.67 to −0.10, *p* = 0.009), though substantial heterogeneity was observed (*I*^2^ = 65.6%, *p* = 0.003) (Figure 7A). Subgroup analysis demonstrated that anthocyanin and isoflavones complementation contributed to a marked decrease in TC levels (Appendix A3, Figure 12.1) and silybin supplementation for <12 weeks was associated with a more marked decrease in TC levels (Appendix A3, Figure 12.2).

Nine trials investigated the effectiveness of flavonoids intervention on TG in 535 patients with NAFLD. Meta-analysis revealed a relevant effect of flavonoids intervention on TG (SMD = −0.37, 95% CI: −0.60 to −0.15, *p* = 0.001), with moderate heterogeneity (*I*^2^ = 39.6%, *p* = 0.104) (Figure 7B). Regarding flavonoids classes, subgroup analysis demonstrated that naringenin, genistein, anthocyanin and isoflavones significantly decreased TG levels (Appendix A3, Figure 13).

## 4 Discussion

NAFLD represents a major worldwide health burden, yet current pharmacological treatment options remain inadequate and effective pharmacological treatments are still limited. Flavonoids mitigate NAFLD through gut-liver axis modulation and multi-target mechanisms. They enrich beneficial bacteria (e.g., Lactobacillus, Bifidobacterium), inhibit pathogens, and enhance gut barrier function, reducing endotoxemia and systemic inflammation. Concurrently, flavonoids activate AMPK/Nrf2 pathways—suppressing SREBP-1c-mediated lipogenesis and boosting antioxidant defenses (SOD, CAT)—while inhibiting NF-κB to attenuate inflammation (TNF-*α*, IL-6). PPAR-α activation promotes fatty acid *β*-oxidation. Improved insulin sensitivity further alleviates steatosis and oxidative stress. Synergistic effects with conventional drugs enhance therapeutic efficacy against NAFLD (19). Specifically, quercetin downregulates SREBP-1c and FAS through the AMPK/SIRT1 signaling pathway to inhibit lipogenesis (20). EGCG enhances its affinity for PPARα and CPT-1 via its galloyl group, thereby promoting mitochondrial fatty acid β-oxidationx (21)_._ Meanwhile, proanthocyanidins increase the abundance of Akkermansia, restore the intestinal barrier, and alleviate LPS-TLR4-mediated hepatic inflammation (22)_._

A comprehensive insight into the efficacy of flavonoids in the treatment of NAFLD and their potential as adjunctive therapies is provided by our updated systematic review and meta-analysis.

The meta-analysis encompassing 25 controlled trials found that flavonoid treatment could significantly reduce ALT, AST, ALP, BMI, TC, TG, FBS, insulin levels, Steatosis score and there was significant increase in QUICKI. To assess the robustness of these core findings against potential biases, we conducted pre-specified sensitivity analyses (by sequentially excluding each study). Reassuringly, the direction and statistical significance of the pooled estimates for these objective biochemical outcomes (e.g., ALT, AST, TG, TC, Insulin, QUICKI) remained stable, indicating that these beneficial effects are robust and unlikely to be driven solely by performance or detection bias present in some included studies. However, there was no significant change in GGT, LDL, HDL, WT, HC, WHtR, HOMA-IR, markers of inflammation, levels of hepatic steatosis and fibrosis.

When significant heterogeneity was observed among the included studies (*I*^2^ > 50%), subgroup analyses were conducted to explore potential sources of variation. Variables such as flavonoid subtype, intervention dosage, duration of treatment, country, age, and sex were examined iteratively to identify specific factors contributing to heterogeneity. The full analytical procedures and detailed results are provided in Appendix A3.

Our analysis revealed that flavonoids supplementation significantly reduced ALT, AST and ALP levels, indicating improved liver function. These findings align with prior meta-analysis examining flavonoid interventions in NAFLD patients (13, 23). Specifically, silymarin demonstrated notable efficacy in reducing these liver enzymes. High-dose silybin (≥2000 mg) produces the greatest reduction in ALT and AST levels. These findings are consistent with prior meta-analytic evidence confirming silymarin’s hepatoprotective effects in NAFLD, including significant improvements in ALT and AST levels, reductions in hepatic steatosis, and decreased liver stiffness (24). While anthocyanin significantly lowers ALT, AST, and ALP in the short term (<12 weeks), making it suitable for patients requiring rapid improvement in liver enzymes. However, the impact on GGT was less pronounced, suggesting that while flavonoids can improve certain aspects of liver function, their effects may be more limited in other areas. In addition, Flavonoid intervention showed no significant improvement in non-invasive fibrosis scores (NFS, FIB-4), likely due to insufficient intervention duration, the inherently slow progression of liver fibrosis, and the limited sensitivity of serum biomarkers for early-stage detection.

Flavonoids supplementation significantly reduce FBS and insulin levels, and increase QUICKI, anthocyanins demonstrated a more pronounced effect in reducing FBS, while improvements in insulin levels were associated with various flavonoids such as hesperidin, genistein, and anthocyanins. Similarly, a meta-analysis reported that interventions rich in flavanols significantly reduced insulin levels and HOMA-IR, with consistent results across different subgroups (25). However, a meta-analysis showed that consumption of flavonoids did not lead to significant changes in insulin, HOMA-IR, and FBS levels in patients with NAFLD (23). These discrepancies may stem from differences in baseline metabolic profiles, as well as variations in study design (e.g., flavonoids dosage, intervention duration, or participant characteristics).

Flavonoids showed a significant reduction in BMI, particularly with hesperidin supplementation, hesperidin monotherapy can simultaneously reduce BMI and waist circumference, with particularly notable efficacy in addressing central obesity, in line with a prior meta-analysis investigating the impact of hesperidin in patients with obesity (26). Notably, hesperidin stimulates the secretion of cholecystokinin (CCK)—a key appetite-regulating hormone—in enteroendocrine STC-1 cells. This CCK-mediated mechanism contributes to its anti-obesity effects by promoting satiety and suppressing appetite (27). However, no significant effects were observed on WC, WHtR, or WT. This suggests that while flavonoids may help in weight management to some extent, their impact on overall body composition may be limited.

Despite no significant overall change in inflammatory markers, subgroup analysis revealed that genistein significantly reduced inflammatory markers. This finding contrasts with a previous meta-analysis, which reported significant reductions in TNF-*α* and NF-κB levels following flavonoids supplementation in NAFLD patients (23). The discrepancy between these results may be attributed to differences in study inclusion - our analysis incorporated 13 additional studies that were not considered in the prior meta-analysis. Genistein, the major soy isoflavone, possesses anticancer, antioxidant, and anti-inflammatory properties, along with tyrosine kinase inhibition (28, 29). Ji et al. demonstrated that genistein alleviates NASH in HFD-fed rats by improving liver function, reduced thiobarbituric acid-reactive substances (TBARS), TNF-α, and IL-6 levels, and inhibiting IκB-α/NF-κB and JNK pathways (30)_._ Although genistein showed a potential reduction in IL-6 in a subgroup analysis, the sample size was too small to support conclusive findings. Flavonoid intervention demonstrates no significant overall effect on inflammatory mediators such as Hs-CRP, IL-6, and TNF-α. This may be attributed to their upstream antioxidant and metabolic roles within the inflammatory cascade, combined with the low baseline inflammation levels in the studied populations, which left limited room for improvement. Furthermore, methodological limitations, including the small number of studies reporting each outcome, inconsistent assay platforms, and single time-point measurement, further reduce statistical power and increase heterogeneity.

Flavonoids significantly reduced TC, TG levels, with naringenin, genistein, anthocyanin, and isoflavones showing the most significant effects. However, the levels of LDL and HDL were not significantly changed. Tsuda et al. reported that anthocyanins upregulated the expression of key adipogenic and metabolic regulators—including PPARγ, uncoupling protein 2 (UCP2), adipocyte fatty acid-binding protein (aP2), and lipoprotein lipase (LPL) —while also stimulating the secretion of adipocytokines (adiponectin and leptin) in isolated rat adipocytes (31). Naringenin attenuates lipid accumulation through AMPK pathway activation, modulating lipid metabolism genes to reduce TC, TG, and non-HDL-C levels. This dual action alleviates hepatic steatosis and decreases adipocyte size in both HFD-fed Sprague–Dawley rats and 3 T3-L1 adipocytes (32). The absence of significant improvements in lipid markers (LDL-C, HDL-C) and anthropometric measures (WT, HC, WHtR) may reflect either interindividual variability in flavonoid response or the necessity of combined dietary/lifestyle modifications to elicit measurable effects.

Nevertheless, this review has several limitations. First, the overall evidence base remains limited by the number and sample size of available RCTs. Significant heterogeneity was observed across several outcomes, which, despite our subgroup analyses, could not be fully resolved due to the variability in flavonoid subtypes, dosages, and patient characteristics. Second, some included studies had a high or unclear risk of bias, particularly in the domains of blinding (performance and detection bias), which could potentially overestimate the intervention effects. However, as demonstrated by our sensitivity analyses, the exclusion of these studies did not alter the significance of our primary findings for key liver and metabolic parameters, suggesting that the core conclusions are resilient to these potential biases. Finally, the generalizability of findings may be constrained by the geographical concentration of studies (e.g., predominantly Iran). While *p*-values alone are insufficient to assess clinical efficacy, meaningful improvements in NAFLD management typically require:a BMI reduction of at least 5%, an ALT decrease of ≥17 U/L, a relative MRI-PDFF reduction of ≥30%, a wc reduction of at least 5 cm, and an overall weight loss of 5–10% (1, 33, 34). After converting our pooled SMDs to absolute units, only the ALT reduction (≈17.5 U/L) approximated its threshold; all other translated benefits—BMI, waist circumference, and lipid parameters fell short of these targets. Consequently, flavonoids remain clinically sub-therapeutic as monotherapy and should be employed solely as adjunctive agents, integrated with foundational diet and exercise interventions to achieve robust, guideline-level NAFLD outcomes.

No significant acute toxicity of flavonoids was observed in the studies included in this analysis. However, their long-term safety remains constrained by metabolic complexity (structural heterogeneity and variations in bioavailability) and the lack of robust methods for assessing *in vivo* oxidative damage (13). These limitations contribute to fragmented data on chronic exposure effects and hinder the objective quantification of metabolic endpoints.

To address these challenges, future research should prioritize: (1) Advanced analytical technologies: The development of high-resolution metabolomics and real-time oxidative stress biomarkers is imperative to elucidate flavonoid absorption, distribution, and excretion dynamics. (2) Large-scale RCTs: Large-scale multicenter randomized controlled trials are crucial to validate long-term efficacy, optimize dosing regimens, and establish safety profiles. Future trials should feature extended durations (≥18 months) and larger cohorts, specifically enrolling patients with liver fibrosis stages F2-F4. Primary endpoints must include objective measures such as histologic improvement (e.g., NASH resolution without fibrosis worsening) or imaging biomarkers (e.g., MRI-PDFF, VCTE-LSM) to generate higher-level evidence. (3) Synergistic therapies: Investigating combinatorial approaches with existing metabolic modulators (e.g., SGLT2 inhibitors or GLP-1 agonists) may enhance therapeutic outcomes in complex disease management.

## 5 Conclusion

Given the growing global use of phytotherapies, healthcare providers should maintain dual competency in both botanical and conventional treatments. Current evidence indicates flavonoid supplementation improves key NAFLD markers, including ALT, AST, ALP, BMI, TC, TG and FBS, Insulin, QUICKI. While flavonoids show therapeutic potential for NAFLD, critical knowledge gaps remain regarding their long-term safety and clinical efficacy. Additionally, structural heterogeneity among flavonoid subclasses, coupled with inherently poor aqueous solubility and low oral bioavailability, significantly limits their potential. Future NAFLD investigations should focus on three key areas: advanced metabolomic profiling of flavonoid pharmacokinetics, multicenter randomized controlled trials to validate clinical outcomes, and synergistic combination therapies with existing metabolic drugs.

## Data Availability

The original contributions presented in the study are included in the article/Supplementary material, further inquiries can be directed to the corresponding author.
