# Supplementary material for: Efficacy of flavonoids in non-alcoholic fatty liver disease: an updated systematic review and meta-analysis
Source: Front Nutr. 2025 Sep 15;12:1660065. doi: 10.3389/fnut.2025.1660065 (PMC12477019; doi:10.3389/fnut.2025.1660065)
Supplement: Supplementary file 1 [file Table_1.DOCX]

| **Flavonoids** | **Author** | **Design** | **Country** | **Population  (IG/CG)** | **Mean age** | | **Gender(M/F)** | | **Intervention** | | **Duration** | **Relevant outcomes** |
| --- | --- | --- | --- | --- | --- | --- | --- | --- | --- | --- | --- | --- |
|  |  |  |  |  | **IG** | **CG** | **IG** | **CG** | **IG** | **CG** |  |  |
| Quercetin | Li et al.2024^[19]^ | Double-Blind RCT, Crossover | China | 41/41 | 39.2± 11.7 | 40.1 ± 9.1 | I:27/14 | C:27/14 | Quercetin 500 mg | Placebo | 12 weeks | WT, HbA1c, ALT  TG, LDL-C |
|  | Hosseinikia et al.2020^[20]^ | Double-Blind RCT, Parallel | Iran | 39/39 | 43.4 ± 11.1 | 45.9 ± 9.2 | I:15/24 | C:13/26 | Quercetin 500 mg | Placebo | 12 weeks | TNF-α, GGT, TG, TC  LDL, HDL, BMI, Hs-CRP, WHR |
|  | Pasdar et al.2020^[21]^ | Double-Blind RCT, Pilot | Iran | 39/39 | 43.46 ± 11.13 | 45.89 ± 9.16 | I:15/24 | C:13/26 | Quercetin 500 mg | Placebo | 12 weeks | RBC, MCHC |
| Genistein | Amanat et al.2018^[22]^ | Double-Blind RCT, Parallel | Iran | 41/41 | 44.22 ± 11.80 | 42.94 ± 9.55 | I:30/11 | C:31/10 | Genistein 250mg | Placebo | 8 weeks | FBS, HOMA-IR, IL-6, TNF-α  WHR, TG, BMI, LDL-C, HDL-C, AST, ALT |
| Naringenin | Namkhah et al.2021^[23]^ | Double-Blind RCT, Parallel | Iran | 22/22 | 44.7 ± 10.7 | 47 ± 9.4 | I:12/10 | C:13/9 | Naringenin 200mg | Placebo | 4 weeks | NFS, WT, BMI, TG, TC, LDL, HDL, WC |
| Hesperidin | Yari et al.2020^[24]^ | Open-labeled  Parallel | Iran | 22/21 | 45.82 ±11.69 | 46.11±11.63 | I:11/11 | C:10/11 | Hesperidin1,000 mg+Lifestyle modification | Lifestyle modification | 12 weeks | HOMA-IR, CRP, TNF-α, NF-κB, Fibrosis score、Steatosis score FLI |
| Anthocyanin | Sangsefidi et al. 2021^[25]^ | Double-Blind RCT, Parallel | Iran | 25/25 | 41.48 ± 9.53 | 42.68 ± 9.96 | I:12/13 | C:11/14 | Anthocyanin 32 mg | Placebo | 12 weeks | ALT, AST, CK-18, Fibrosis score, Steatosis score |
|  | Yarhosseini et al. 2021^[26]^ | Double-Blind RCT, Parallel | Iran | 25/25 | 41.4 ± 9.5 | 42.6 ± 9.9 | I:12/13 | C:11/14 | Anthocyanin 32 mg | Placebo | 12 weeks | WC, HC, WHR |
|  | Izadi et al. 2020^[27]^ | Double-Blind RCT, Parallel | Iran | 30/31 | 43.3 ± 10.2 | 42.8 ± 10.6 | I:17/13 | C:19/12 | Anthocyanin 250 mg | Placebo | 8 weeks | TG, ALT, AST, TC, LDL-C, HDL-C, WT, BMI, WC |
|  | Zhang et al. 2014^[28]^ | Double-Blind RCT, Parallel | China | 37/37 | 44.9 ± 7.5 | 46.9 ± 7.7 | I:19/18 | C:20/17 | Anthocyanin 320 mg | Placebo | 12 weeks | ALT, AST, TG, TC, LDL, HDL-C, BMI, CK-18, HOMA-IR, WHR |
|  | Bayram et al. 2024^[29]^ | RCT, Parallel | Turkey | 22/22 | 43.90 ± 10.44 | 43.40 ± 12.46 | I:10/12 | C:10/12 | Anthocyanin 350 mg | Placebo | 8 weeks | ALT, AST, ALP, GGT, TG, TC, LDL, HDL, FBG, HbA1c, HOMA-IR, CRP, BMI, WC, HC |
|  | Ghanbari et al. 2024^[30]^ | Double-Blind RCT, Parallel | Iran | 25/25 | 43.52 ± 8.12 | 44.88 ± 10.14 | I:10/15 | C:14/11 | Grape Seed Extract 520 mg | Placebo | 8 weeks | ALT, AST, TG, TC, LDL-c, HDL-c, BMI, QUICK, HOMA-IR |
| Pueraria | Li et al. 2024^[31]^ | Triple-blind RCT, Parallel | China | 60/61 | 56.2 ± 10 | 58.1 ± 9.6 | I:30/30 | C:31/30 | Silybin 138.0 mg, Puerarin 68.4 mg, Salvianolic acid 65.4 mg | Placebo | 24 weeks | FIB-4, HOMA-IR, WT, BMI, WC, WHR, CRP, IL-6, FIB-4, APRI, NFS |
| **Flavonoids** | **Author** | **Design** | **Country** | **Population  (IG/CG)** | **Mean age** | | **Gender(M/F)** | | **Intervention** | | **Duration** | **Relevant outcomes** |
|  |  |  |  |  | **IG** | **CG** | **IG** | **CG** | **IG** | **CG** |  |  |
| Isoflavones | Tehrani et al. 2024^[32]^ | Double-Blind RCT, Parallel | Iran | 25/21 | F:51.93±11.15, M:47.60 ± 14.98 | F:52.09 ±5.73 M:46.0 ±14.10 | I:10/15 | C:10/11 | Soy isoflavones 100 mg | Placebo | 12 weeks | ALT, AST, Steatosis score,Fibrosis score, GGT, FGF-21, WT, BMI, WC, WHR |
| Catechin | Pezeshki et al. 2016^[33]^ | Double-Blind RCT, Parallel | Iran | 35/36 | None | None | I:16/19 | C:16/20 | Catechins 3.45 mg Caffeine 11.375 mg，GC：25.66 mg，EGC：15.38 mg， EC：29.305 mg，EGCG：157.145 mg，GCG：11.45 mg，ECG：13.235 mg | Placebo | 90 days | AST, ALT, ALP, WT, BMI |
| Silymarin | Hashemi et al. 2009^[34]^ | Double-Blind RCT, Parallel | Iran | 50/50 | 39.28 ± 11.117 | 39.0 ± 10.70 | I:28/22 | C:29/21 | Silymarin 280 mg | Placebo | 24 weeks | AST, ALT, FBS, TG, TC, LDL, HDL, BMI |
|  | Solhi et al.2014^[35]^ | RCT, Parallel | Iran | 33/31 | 43.6 ± 8.3 | 39.36 ± 10.5 | I:19/14 | C:19/12 | Silymarin 210 mg | Placebo | 8 weeks | AST, ALT |
|  | Anushiravani et al. 2019^[36]^ | Double-Blind RCT, Parallel | Iran | 30/30 | None | None | None | None | Silymarin 140 mg | Placebo | 12 weeks | BMI, WC, TG, TC, LDL-C, HDL-C, FBS |
|  | Aller et al. 2015^[37]^ | RCT, Parallel | Spain | 18/18 | None | None | None | None | Silymarin 1080.6 mg+ Vitamin E 72 mg+Lifestyle modification | Lifestyle modification | 12 weeks | BMI, WC, GGT, FLI, NFS, ALT, AST, TG, FBS, HOMA-IR, WT |
|  | Memon et al. 2015^[38]^ | Double-Blind RCT, Parallel | Pakistan | 31/33 | 49.0 ± 9.70 | 48 ± 8.9 | I:21/12 | C:21/10 | Silymarin 280 mg | Placebo | 3 months | ALT, AST, TG, TC, LDL, HDL |
|  | Rangboo et al. 2016^[39]^ | Double-Blind RCT, Parallel | Iran | 30/30 | 47.27 ± 8.12 | 49.83 ± 12.79 | I:21/9 | C:21/9 | 2700mg Cynara scolymus extract | Placebo | 2 months | ALT, AST, TG, TC, LDL-C, HDL-C, FBS |
|  | Shaikh et al. 2021^[40]^ | RCT, Parallel | Pakistan | 100/100 | None | None | None | None | Silymarin 400mg | Placebo | 12 weeks | AST,ALT |
|  | Mirhashemi et al.2022^[41]^ | Double-Blind RCT, Parallel | Iran | 27/25 | 37.81 ± 9.93 | 38.08 ±10.01 | None | None | 560 mg Silymarin+Lifestyle modification | Placebo +Lifestyle modification | 8 weeks | AST, ALT, AST/ALT, BMI, Fib-4, NFS |
|  | Atarodi et al. 2022^[42]^ | Double-Blind RCT, Parallel | Iran | 27/29 | 36.46±10.00 | 37.52±8.94 | I:9/18 | C:8/21 | Silymarin 140mg | Placebo | 4 weeks | ALT, AST, ALP, TB, DB, TG, TC, LDL-C, HDL-C, BMI |
|  | Hafiza et al. 2024^[43]^ | RCT, Parallel | Pakistan | I1:16 I2:16 I3:16  C:16 | None | None | I1:7/9   I2:6/10 I3:8/8 | C:8/8 | I1:Silymarin 200mg I2:Silymarin 300mg I3:Silymarin 400mg | Placebo | 3 months | ALT, AST, ALP, CRP, ESR, BMI |
| AST:Aspartate Aminotransferase; ALT:Alanine Aminotransferase; ALP:Alkaline Phosphatase; GGT:G-Glutamyl-Transferase, BMI:Body Mass Index; WT:Weight; HC:Hip Circumference; WC:Waist Circumference; WHR:Waist-to-Hip Ratio; CRP;C-Reactive Protein; TNF-α:tumor necrosis factor; IL-6:Interleukin-6; CK-18:Cytokeratin-18; LDL-C:Low-Density lipo-Protein Cholesterol; TC:Total Cholesterol; HDL-C: High-Density Lipoprotein Cholesterol; TG: Triglycerides; FBS:Fasting Blood Sugar; HOMA-IR:Homeostatic Model Assessment of Insulin Resistance; QUICKI:Quantitative Insulin Sensitivity Check Index; IG:Intervention group; CG:Control group; I:Intervention; C:Control | | | | | | | | | | | | |
