# Supplementary material for: Efficacy of flavonoids in non-alcoholic fatty liver disease: an updated systematic review and meta-analysis
Source: Front Nutr. 2025 Sep 15;12:1660065. doi: 10.3389/fnut.2025.1660065 (PMC12477019; doi:10.3389/fnut.2025.1660065)
Supplement: Supplementary file 2 [file Table_2.DOCX]

**Appendix A1**

| Query | Source | Results | Date |
| --- | --- | --- | --- |
| (Isoflavones[MH] OR quercetin[MH] OR naringenin[MH] OR catechin[MH] OR puerarin[MH] OR rutin[MH] OR luteolin[MH] OR baicalein[MH] OR OR silymarin[MH] OR anthocyanins[MH] OR 7-Rhamnoglucoside, Hesperetin[MH] OR soy[TIAB] OR soya[TIAB]) | PubMed | 89058 | 12/19/24 |
| (Non-alcoholic Fatty Liver Disease[MH] OR Nonalcoholic Steatohepatiti*[TIAB)OR Nonalcoholic Fatty Liver*[TIAB] OR Nonalcoholic Fatty Liver Disease[TIAB] OR Non alcoholic Fatty Liver Disease[TIAB)OR NAFLD[TIAB] | PubMed | 46556 | 12/19/24 |
| "rimostil"[Title/Abstract] OR "promensil"[Title/Abstract] OR "novasoy"[Title/Abstract] OR "Isoflavone*"[Title/Abstract] OR "5 hydroxy 7 prenyloxyisoflavone"[Title/Abstract] OR "3 phenylchromone"[Title/Abstract] OR "3 Benzylidene 4 Chromanone*"[Title/Abstract] OR "sophoretin"[Title/Abstract] OR "quertine"[Title/Abstract] OR "quercitin"[Title/Abstract] OR "quercetol*"[Title/Abstract] OR "quercetin*"[Title/Abstract] OR "meltin"[Title/Abstract] OR "meletin"[Title/Abstract] OR "flavin"[Title/Abstract] OR "dikvertin"[Title/Abstract] OR "naringoside"[Title/Abstract] OR "naringin"[Title/Abstract] OR "Naringenin"[Title/Abstract] OR "isohesperidin"[Title/Abstract] OR "aurantium"[Title/Abstract] OR "auranthin*"[Title/Abstract] OR "tea"[Title/Abstract] OR "kb53"[Title/Abstract] OR "green tea"[Title/Abstract] OR "epicatechin"[Title/Abstract] OR "cianidanol"[Title/Abstract] OR "catergen"[Title/Abstract] OR "catechuic acid"[Title/Abstract] OR "pueraria"[Title/Abstract] OR "kudzu"[Title/Abstract] OR "kankonein"[Title/Abstract] OR "gegen"[Title/Abstract] OR "vitamin P"[Title/Abstract] OR "violaquercitrin"[Title/Abstract] OR "tanrutin"[Title/Abstract] OR "sophorin"[Title/Abstract] OR "sclerutin"[Title/Abstract] OR "rutoside"[Title/Abstract] OR "rutabion"[Title/Abstract] OR "osyritrin"[Title/Abstract] OR "myrticolorin"[Title/Abstract] OR "melin"[Title/Abstract] OR "citroflavone"[Title/Abstract] OR "birutan"[Title/Abstract] OR "luteoline"[Title/Abstract] OR "digitoflavone"[Title/Abstract] OR "cyanidenon"[Title/Abstract] OR "3',4',5,7 tetrahydroxy flavone"[Title/Abstract] OR "5, 6, 7 trihydroxy flavone"[Title/Abstract] OR "baicaleine"[Title/Abstract] OR "noroxylin"[Title/Abstract] OR "karsil"[Title/Abstract] OR "silimarin"[Title/Abstract] OR "simepar"[Title/Abstract] OR "silymarin*"[Title/Abstract] OR "silliver"[Title/Abstract] OR "silepar"[Title/Abstract] OR "legalon"[Title/Abstract] OR "flavobion"[Title/Abstract] OR "carsil"[Title/Abstract] OR "anthocyanidin*"[Title/Abstract] OR "anthocyanin*"[Title/Abstract] OR "leucoanthocyanidins"[Title/Abstract] OR "7-Rhamnoglucoside"[Title/Abstract] OR "hesperidin"[Title/Abstract] | PubMed | 127268 | 12/19/24 |
| (randomized controlled trial[PT] OR controlled clinical trial[PT] OR clinical trial[PT] OR clinical study[PT] OR  randomized[TIAB] OR placebo[TIAB] OR randomly[TIAB] OR trial[TIAB] OR blind[TIAB] OR groups[TIAB] OR controlled  clinical trials as topic[MH] OR randomized controlled trial[MH] OR placebos[MH] OR double-blind method[MH]) | PubMed | 4512691 | 12/19/24 |
| ((((((((((((((((Isoflavones[MeSH Terms])) OR (quercetin[MeSH Terms])) OR (naringenin[MeSH Terms])) OR (catechin[MeSH Terms])) OR (puerarin[MeSH Terms])) OR (rutin[MeSH Terms])) OR (luteolin[MeSH Terms])) OR (baicalein[MeSH Terms])) OR (silymarin[MeSH Terms])) OR (anthocyanins[MeSH Terms])) OR (7-Rhamnoglucoside, Hesperetin[MeSH Terms])) OR (soy[Title/Abstract])) OR (soya[Title/Abstract])) OR ("rimostil"[Title/Abstract] OR "promensil"[Title/Abstract] OR "novasoy"[Title/Abstract] OR "Isoflavone*"[Title/Abstract] OR "5 hydroxy 7 prenyloxyisoflavone"[Title/Abstract] OR "3 phenylchromone"[Title/Abstract] OR "3 Benzylidene 4 Chromanone*"[Title/Abstract] OR "sophoretin"[Title/Abstract] OR "quertine"[Title/Abstract] OR "quercitin"[Title/Abstract] OR "quercetol*"[Title/Abstract] OR "quercetin*"[Title/Abstract] OR "meltin"[Title/Abstract] OR "meletin"[Title/Abstract] OR "flavin"[Title/Abstract] OR "dikvertin"[Title/Abstract] OR "naringoside"[Title/Abstract] OR "naringin"[Title/Abstract] OR "Naringenin"[Title/Abstract] OR "isohesperidin"[Title/Abstract] OR "aurantium"[Title/Abstract] OR "auranthin*"[Title/Abstract] OR "tea"[Title/Abstract] OR "kb53"[Title/Abstract] OR "green tea"[Title/Abstract] OR "epicatechin"[Title/Abstract] OR "cianidanol"[Title/Abstract] OR "catergen"[Title/Abstract] OR "catechuic acid"[Title/Abstract] OR "pueraria"[Title/Abstract] OR "kudzu"[Title/Abstract] OR "kankonein"[Title/Abstract] OR "gegen"[Title/Abstract] OR "vitamin P"[Title/Abstract] OR "violaquercitrin"[Title/Abstract] OR "tanrutin"[Title/Abstract] OR "sophorin"[Title/Abstract] OR "sclerutin"[Title/Abstract] OR "rutoside"[Title/Abstract] OR "rutabion"[Title/Abstract] OR "osyritrin"[Title/Abstract] OR "myrticolorin"[Title/Abstract] OR "melin"[Title/Abstract] OR "citroflavone"[Title/Abstract] OR "birutan"[Title/Abstract] OR "luteoline"[Title/Abstract] OR "digitoflavone"[Title/Abstract] OR "cyanidenon"[Title/Abstract] OR "3',4',5,7 tetrahydroxy flavone"[Title/Abstract] OR "5, 6, 7 trihydroxy flavone"[Title/Abstract] OR "baicaleine"[Title/Abstract] OR "noroxylin"[Title/Abstract] OR "karsil"[Title/Abstract] OR "silimarin"[Title/Abstract] OR "simepar"[Title/Abstract] OR "silymarin*"[Title/Abstract] OR "silliver"[Title/Abstract] OR "silepar"[Title/Abstract] OR "legalon"[Title/Abstract] OR "flavobion"[Title/Abstract] OR "carsil"[Title/Abstract] OR "anthocyanidin*"[Title/Abstract] OR "anthocyanin*"[Title/Abstract] OR "leucoanthocyanidins"[Title/Abstract] OR "7-Rhamnoglucoside"[Title/Abstract] OR "hesperidin"[Title/Abstract])) AND ((Non-alcoholic Fatty Liver Disease[MeSH Terms]) OR ("Nonalcoholic Steatohepatiti*"[Title/Abstract] OR "Nonalcoholic Fatty Liver*"[Title/Abstract] OR "Non alcoholic Fatty Liver Disease"[Title/Abstract] OR "NAFLD"[Title/Abstract]))) AND ((randomized controlled trial[Publication Type]) OR (controlled clinical trial[Publication Type]) OR (clinical trial[Publication Type])OR (clinical study[Publication Type]) OR (randomized[Title/Abstract])OR (placebo[Title/Abstract])OR (randomly[Title/Abstract]) OR (trial[Title/Abstract]) OR (blind[Title/Abstract]) OR (groups[Title/Abstract]) OR (controlled clinical trials as topic[MeSH Terms]) OR (randomized controlled trial[MeSH Terms]) OR (placebos[MeSH Terms]) OR (double-blind method[MeSH Terms])) | PubMed | 263 | 12/19/24 |
| #1 MeSH descriptor: [Isoflavones] explode all trees | [Cochrane](https://www-cochranelibrary-com.uniroma.laoxuean.vip/) | 867 | 12/21/24 |
| #2 MeSH descriptor: [Quercetin] explode all trees | [Cochrane](https://www-cochranelibrary-com.uniroma.laoxuean.vip/) | 279 | 12/21/24 |
| #3 MeSH descriptor: [Catechin] explode all trees | [Cochrane](https://www-cochranelibrary-com.uniroma.laoxuean.vip/) | 459 | 12/21/24 |
| #4 MeSH descriptor: [Rutin] explode all trees | [Cochrane](https://www-cochranelibrary-com.uniroma.laoxuean.vip/) | 211 | 12/21/24 |
| #5 MeSH descriptor: [Luteolin] explode all trees | [Cochrane](https://www-cochranelibrary-com.uniroma.laoxuean.vip/) | 16 | 12/21/24 |
| #6 MeSH descriptor: [Silymarin] explode all trees | [Cochrane](https://www-cochranelibrary-com.uniroma.laoxuean.vip/) | 227 | 12/21/24 |
| #7 MeSH descriptor: [Anthocyanins] explode all trees | [Cochrane](https://www-cochranelibrary-com.uniroma.laoxuean.vip/) | 234 | 12/21/24 |
| #8 MeSH descriptor: [Hesperidin] explode all trees | [Cochrane](https://www-cochranelibrary-com.uniroma.laoxuean.vip/) | 110 | 12/21/24 |
| #9 (rimostil):ti,ab,kw OR (promensil):ti,ab,kw OR (novasoy):ti,ab,kw OR (Isoflavone*):ti,ab,kw OR (5 hydroxy 7 prenyloxyisoflavone):ti,ab,kw | [Cochrane](https://www-cochranelibrary-com.uniroma.laoxuean.vip/) | 1328 | 12/21/24 |
| #10 (3 phenylchromone):ti,ab,kw OR (3 Benzylidene 4 Chromanone*):ti,ab,kw OR (sophoretin):ti,ab,kw OR (quertine):ti,ab,kw OR (quercitin):ti,ab,kw | [Cochrane](https://www-cochranelibrary-com.uniroma.laoxuean.vip/) | 7 | 12/21/24 |
| #11 (quercetol*):ti,ab,kw OR (quercetin*):ti,ab,kw OR (meltin):ti,ab,kw OR (meletin):ti,ab,kw OR (flavin):ti,ab,kw | [Cochrane](https://www-cochranelibrary-com.uniroma.laoxuean.vip/) | 842 | 12/21/24 |
| #12 (dikvertin):ti,ab,kw OR (naringoside):ti,ab,kw OR (naringin):ti,ab,kw OR (Naringenin):ti,ab,kw OR (isohesperidin):ti,ab,kw | [Cochrane](https://www-cochranelibrary-com.uniroma.laoxuean.vip/) | 97 | 12/21/24 |
| #13 (aurantium):ti,ab,kw OR (auranthin*):ti,ab,kw OR (tea):ti,ab,kw OR (kb53):ti,ab,kw OR (green tea):ti,ab,kw | [Cochrane](https://www-cochranelibrary-com.uniroma.laoxuean.vip/) | 4766 | 12/21/24 |
| #14 (epicatechin):ti,ab,kw OR (cianidanol):ti,ab,kw OR (catergen):ti,ab,kw OR (catechuic acid):ti,ab,kw OR (pueraria):ti,ab,kw | [Cochrane](https://www-cochranelibrary-com.uniroma.laoxuean.vip/) | 356 | 12/21/24 |
| #15 (kankonein):ti,ab,kw OR (kudzu):ti,ab,kw OR (gegen):ti,ab,kw OR (vitamin P):ti,ab,kw OR (violaquercitrin):ti,ab,kw | [Cochrane](https://www-cochranelibrary-com.uniroma.laoxuean.vip/) | 15626 | 12/21/24 |
| #16 (tanrutin):ti,ab,kw OR (sophorin):ti,ab,kw OR (sclerutin):ti,ab,kw OR (rutoside):ti,ab,kw OR (rutabion):ti,ab,kw | [Cochrane](https://www-cochranelibrary-com.uniroma.laoxuean.vip/) | 80 | 12/21/24 |
| #17 (osyritrin):ti,ab,kw OR (myrticolorin):ti,ab,kw OR (melin):ti,ab,kw OR (citroflavone):ti,ab,kw OR (birutan):ti,ab,kw | [Cochrane](https://www-cochranelibrary-com.uniroma.laoxuean.vip/) | 5 | 12/21/24 |
| #18 (luteoline):ti,ab,kw OR (digitoflavone):ti,ab,kw OR (cyanidenon):ti,ab,kw OR (3,4,5,7 tetrahydroxy flavone):ti,ab,kw OR (5,6,7 trihydroxy flavone):ti,ab,kw | [Cochrane](https://www-cochranelibrary-com.uniroma.laoxuean.vip/) | 2 | 12/21/24 |
| #19 (baicaleine):ti,ab,kw OR (noroxylin):ti,ab,kw OR (karsil):ti,ab,kw OR (silimarin):ti,ab,kw OR (simepar):ti,ab,kw | [Cochrane](https://www-cochranelibrary-com.uniroma.laoxuean.vip/) | 8 | 12/21/24 |
| #20 (silymarin*):ti,ab,kw OR (silliver):ti,ab,kw OR (silepar):ti,ab,kw OR (legalon):ti,ab,kw AND (flavobion):ti,ab,kw | [Cochrane](https://www-cochranelibrary-com.uniroma.laoxuean.vip/) | 504 | 12/21/24 |
| #21 (carsil):ti,ab,kw OR (anthocyanidin*):ti,ab,kw OR (anthocyanin*):ti,ab,kw OR (leucoanthocyanidins):ti,ab,kw OR (hesperidin):ti,ab,kw | [Cochrane](https://www-cochranelibrary-com.uniroma.laoxuean.vip/) | 995 | 12/21/24 |
| #22 MeSH descriptor: [Non-alcoholic Fatty Liver Disease] explode all trees | [Cochrane](https://www-cochranelibrary-com.uniroma.laoxuean.vip/) | 1987 | 12/21/24 |
| #23 (Nonalcoholic Steatohepatiti*):ti,ab,kw OR (Nonalcoholic Fatty Liver*):ti,ab,kw OR (Nonalcoholic Fatty Liver Disease):ti,ab,kw OR (Non alcoholic Fatty Liver Disease):ti,ab,kw OR (NAFLD):ti,ab,kw | [Cochrane](https://www-cochranelibrary-com.uniroma.laoxuean.vip/) | 5506 | 12/21/24 |
| #24 MeSH descriptor: [Randomized Controlled Trial] explode all trees | [Cochrane](https://www-cochranelibrary-com.uniroma.laoxuean.vip/) | 37 | 12/21/24 |
| #25 (controlled clinical trials as topic):ti,ab,kw OR (clinical trial):ti,ab,kw OR (clinical study):ti,ab,kw OR (clinical study):ti,ab,kw OR (clinical study):ti,ab,kw | [Cochrane](https://www-cochranelibrary-com.uniroma.laoxuean.vip/) | 1005265 | 12/21/24 |
| #26 #22OR#23 | [Cochrane](https://www-cochranelibrary-com.uniroma.laoxuean.vip/) | 5506 | 12/21/24 |
| #27 #24OR#25 | [Cochrane](https://www-cochranelibrary-com.uniroma.laoxuean.vip/) | 1005292 | 12/21/24 |
| #28 #1OR#2OR#3OR#4OR#5OR#6OR#7OR#8OR#9OR#10OR#11OR#12OR#13OR#14OR#15OR#16OR#17OR#18OR#19OR#20OR#21 | [Cochrane](https://www-cochranelibrary-com.uniroma.laoxuean.vip/) | 24487 | 12/21/24 |
| #29 #26AND#27AND#28 | [Cochrane](https://www-cochranelibrary-com.uniroma.laoxuean.vip/) | 248 | 12/21/24 |
| #1TS=(Isoflavones OR quercetin OR naringenin OR catechin OR puerarin OR rutin OR luteolin OR baicalein OR silymarin OR anthocyanins OR 7-Rhamnoglucoside, #2Hesperetin) and Preprint Citation Index (Exclude – Database) | Web of Science | 236249 | 12/22/24 |
| #2AB=(rimostil OR promensil OR novasoy OR Isoflavone* OR 5 hydroxy 7 prenyloxyisoflavone OR 3 phenylchromone OR 3 Benzylidene 4 Chromanone* OR soph OR etin OR quertine OR quercitin OR quercetol* OR quercetin* OR meltin OR meletin OR flavin OR dikvertin OR naringoside OR naringin OR Naringenin OR isohesperidin OR aurantium OR auranthin* OR tea OR kb53 OR green tea OR epicatechin OR cianidanol OR catergen OR catechuic acid OR pueraria OR kudzu OR kankonein OR gegen OR vitamin P OR violaquercitrin OR tanrutin OR soph OR in OR sclerutin OR rutoside OR rutabion OR osyritrin OR myrticol OR in OR melin OR citroflavone OR birutan OR luteoline OR digitoflavone OR cyanidenon OR 3,4,5,7 tetrahydroxy flavone OR 5,6,7 trihydroxy flavone OR baicaleine OR n OR oxylin OR karsil OR silimarin OR simepar OR silymarin* OR silliver OR silepar OR legalon OR flavobion OR carsil OR anthocyanidin* OR anthocyanin* OR leucoanthocyanidins OR hesperidin) and Preprint Citation Index (Exclude – Database) | Web of Science | 120230431 | 12/22/24 |
| #3TS=(Non-alcoholic Fatty Liver Disease)) OR AB=(Nonalcoholic Steatohepatiti* OR Nonalcoholic Fatty Liver* OR Nonalcoholic Fatty Liver Disease OR Non alcoholic Fatty Liver Disease OR NAFLD) and Preprint Citation Index (Exclude – Database) | Web of Science | 83587 | 12/22/24 |
| #4TS=(Randomized Controlled Trial)) OR AB=(controlled clinical trials as topic or clinical trial or clinical study or placebos or double-blind method) and Preprint Citation Index (Exclude – Database) | Web of Science | 4434654 | 12/22/24 |
| #5 #1OR#2 and Preprint Citation Index (Exclude – Database) | Web of Science | 120249027 | 12/22/24 |
| #6 #3 AND #4 AND #5 and Preprint Citation Index (Exclude – Database) | Web of Science | 11974 | 12/22/24 |
| #7 #3 AND #4 AND #5 and Preprint Citation Index (Exclude – Database) and Clinical Trial (Document Types) | Web of Science | 993 | 12/22/24 |
|  |  |  |  |
| #1 'isoflavone derivative'/exp | Embase | 8311 | 12/21/24 |
| #2 'quercetin'/exp | Embase | 49759 | 12/21/24 |
| #3 'naringenin'/exp | Embase | 8061 | 12/21/24 |
| #4 'catechin'/exp | Embase | 19544 | 12/21/24 |
| #5 'diabetic retinopathy'/exp | Embase | 61895 | 12/21/24 |
| #6 'puerarin'/exp | Embase | 3374 | 12/21/24 |
| #7 'diabetic retinopathy'/exp | Embase | 61895 | 12/21/24 |
| #8 'rutoside'/exp | Embase | 16354 | 12/21/24 |
| #9 'diabetic retinopathy'/exp | Embase | 61895 | 12/21/24 |
| #10 'luteolin'/exp | Embase | 12504 | 12/21/24 |
| #11 'baicalein'/exp | Embase | 5394 | 12/21/24 |
| #12 'silymarin'/exp | Embase | 8611 | 12/21/24 |
| #13 'anthocyanin'/exp | Embase | 19178 | 12/21/24 |
| #14 'hesperidin'/exp | Embase | 5636 | 12/21/24 |
| #15 'rimostil':ti,kw,ab OR 'promensil':ti,kw,ab OR 'novasoy':ti,kw,ab OR 'isoflavone*':ti,kw,ab OR '5 hydroxy 7 prenyloxyisoflavone':ti,kw,ab OR '3 phenylchromone':ti,kw,ab OR '3 benzylidene 4 chromanone*':ti,kw,ab OR 'sophoretin':ti,kw,ab OR 'quertine':ti,kw,ab OR 'quercitin':ti,kw,ab OR 'quercetol*':ti,kw,ab OR 'quercetin*':ti,kw,ab OR 'meltin':ti,kw,ab OR 'meletin':ti,kw,ab OR 'flavin':ti,kw,ab OR 'dikvertin':ti,kw,ab OR 'naringoside':ti,kw,ab OR 'naringin':ti,kw,ab OR 'naringenin':ti,kw,ab OR 'isohesperidin':ti,kw,ab OR 'aurantium':ti,kw,ab OR 'auranthin*':ti,kw,ab OR 'tea':ti,kw,ab OR 'kb53':ti,kw,ab OR 'green tea':ti,kw,ab OR 'epicatechin':ti,kw,ab OR 'cianidanol':ti,kw,ab OR 'catergen':ti,kw,ab OR 'catechuic acid':ti,kw,ab OR 'pueraria':ti,kw,ab OR 'kudzu':ti,kw,ab OR 'kankonein':ti,kw,ab OR 'gegen':ti,kw,ab OR 'vitamin p':ti,kw,ab OR 'violaquercitrin':ti,kw,ab OR 'tanrutin':ti,kw,ab OR 'sophorin':ti,kw,ab OR 'sclerutin':ti,kw,ab OR 'rutoside':ti,kw,ab OR 'rutabion':ti,kw,ab OR 'osyritrin':ti,kw,ab OR 'myrticolorin':ti,kw,ab OR 'melin':ti,kw,ab OR 'citroflavone':ti,kw,ab OR 'birutan':ti,kw,ab OR 'luteoline':ti,kw,ab OR 'digitoflavone':ti,kw,ab OR 'cyanidenon':ti,kw,ab OR '3,4,5,7 tetrahydroxy flavone':ti,kw,ab OR '5,6,7 trihydroxy flavone':ti,kw,ab OR 'baicaleine':ti,kw,ab OR 'noroxylin':ti,kw,ab OR 'karsil':ti,kw,ab OR 'silimarin':ti,kw,ab OR 'simepar':ti,kw,ab OR 'silymarin*':ti,kw,ab OR 'silliver':ti,kw,ab OR 'silepar':ti,kw,ab OR 'legalon':ti,kw,ab OR 'flavobion':ti,kw,ab OR 'carsil':ti,kw,ab OR 'anthocyanidin*':ti,kw,ab OR 'anthocyanin*':ti,kw,ab OR 'leucoanthocyanidins':ti,kw,ab OR 'hesperidin':ti,kw,ab | Embase | 151064 | 12/21/24 |
| #16 'non-alcoholic fatty liver disease'/exp | Embase | 78715 | 12/21/24 |
| #17 'nonalcoholic steatohepatiti*':ti,kw,ab OR 'nonalcoholic fatty liver*':ti,kw,ab OR 'nonalcoholic fatty liver disease':ti,kw,ab OR  'non alcoholic fatty liver disease':ti,kw,ab OR 'nafld':ti,kw,ab | Embase | 68742 | 12/21/24 |
| #18 'randomized controlled trial'/exp | Embase | 859530 | 12/21/24 |
| #19 'controlled clinical trials as topic':ti,ab,kw OR 'clinical trial or clinical study':ti,ab,kw OR 'placebos':ti,ab,kw OR 'double-blind method':ti,ab,kw | Embase | 4855 | 12/21/24 |
| #20 #1 OR #2 OR #3 OR #4 OR #5 OR #6 OR #7 OR #8 OR #9 OR #10 OR #11 OR #12 OR #13 OR #14 | Embase | 184027 | 12/21/24 |
| #21 #15 OR #20 | Embase | 265933 | 12/21/24 |
| #22 #16 OR #17 | Embase | 89074 | 12/21/24 |
| #23 #18 OR #19 | Embase | 862942 | 12/21/24 |
| #24 #21 AND #22 AND #23 | Embase | 74 | 12/21/24 |
|  |  |  |  |
| 1 exp Isoflavones/ | Ovid | 21269 | 12/23/24 |
| 2 exp Quercetin/ | Ovid | 13268 | 12/23/24 |
| 3 exp Rutin/ | Ovid | 4278 | 12/23/24 |
| 4 exp Luteolin/ | Ovid | 2523 | 12/23/24 |
| 5 exp Silymarin/ | Ovid | 3158 | 12/23/24 |
| 6 exp Anthocyanins/ | Ovid | 11357 | 12/23/24 |
| 7 exp Hesperidin/ | Ovid | 2118 | 12/23/24 |
| 8 (rimostil or promensil or novasoy or Isoflavone* or soph or etin or quertine or quercitin or quercetol* or quercetin* or meltin or meletin or flavin or dikvertin or naringoside or naringin or Naringenin or isohesperidin or aurantium or auranthin* or epicatechin or cianidanol or catergen or catechuic acid or pueraria or kudzu or kankonein or gegen or vitamin P or violaquercitrin or tanrutin or soph or in or sclerutin or rutoside or rutabion or osyritrin or myrticol or in or melin or citroflavone or birutan or luteoline or digitoflavone or cyanidenon or baicaleine or karsil or silimarin or simepar or silymarin* or silliver or silepar or legalon or flavobion or carsil or anthocyanidin* or anthocyanin* or leucoanthocyanidins or hesperidin).ti,kw,ab. | Ovid | 29761426 | 12/23/24 |
| 9 exp Non-alcoholic Fatty Liver Disease/ | Ovid | 27998 | 12/23/24 |
| 10 (Nonalcoholic Steatohepatiti* or Nonalcoholic Fatty Liver* or Nonalcoholic Fatty Liver Disease or Non alcoholic Fatty Liver Disease or NAFLD).ti,kw,ab. | Ovid | 42473 | 12/23/24 |
| 11 exp Randomized Controlled Trial/ | Ovid | 630304 | 12/23/24 |
| 12(controlled clinical trials as topic or clinical trial or clinical study or placebos or double-blind method).ti,kw,ab. | Ovid | 290700 | 12/23/24 |
| 1 or 2 or 3 or 4 or 5 or 6 or 7 or 8 | Ovid | 29763790 | 12/23/24 |
| 9 or 10 | Ovid | 46454 | 12/23/24 |
| 11 or 12 | Ovid | 832745 | 12/23/24 |
| 13 and 14 and 15 | Ovid | 1541 | 12/23/24 |

Appendix A2

**1 Alanine Aminotransferase（ALT)**


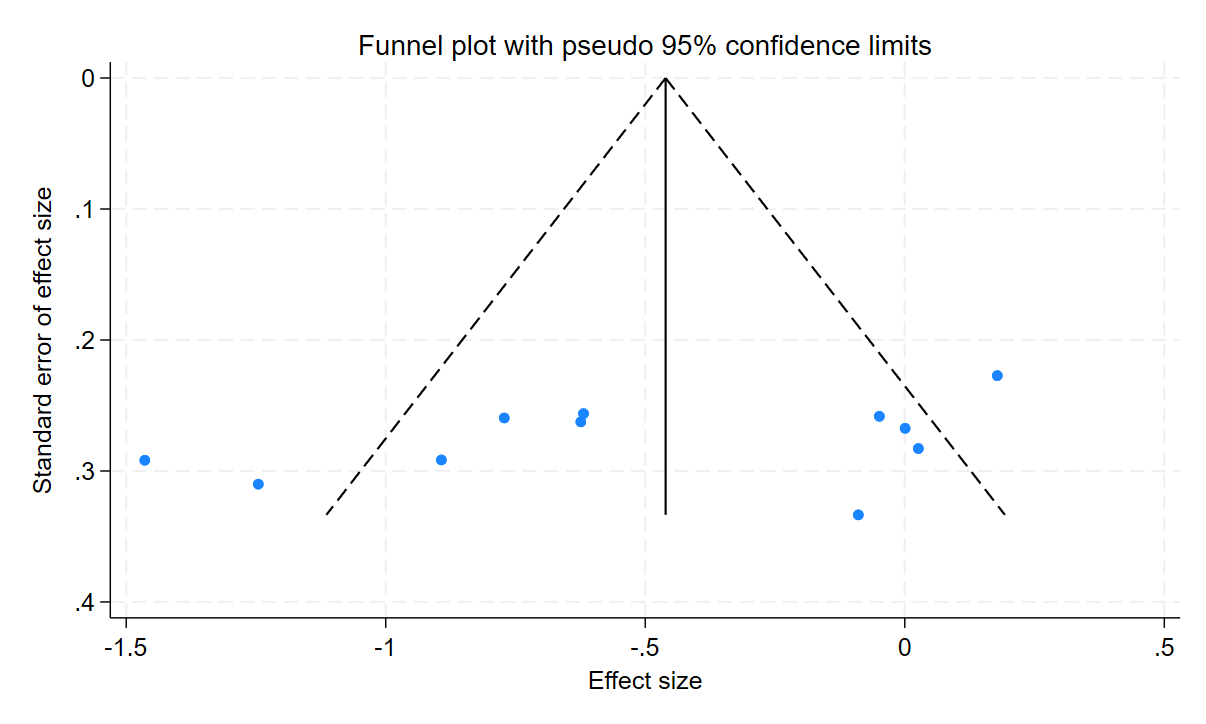


**Fig 1.1** ALT-Funnel plot analysis


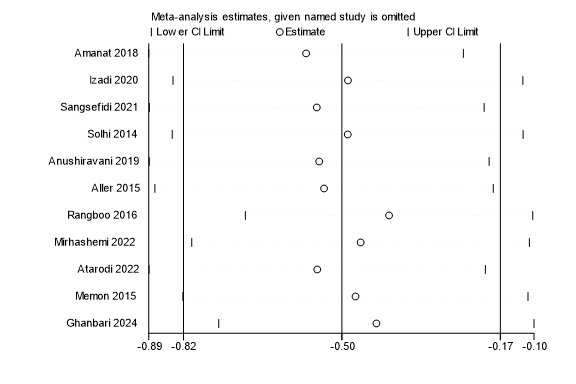


**Fig 1.2** ALT-Sensitive analysis

**2 Aspartate Aminotransferase(AST)**


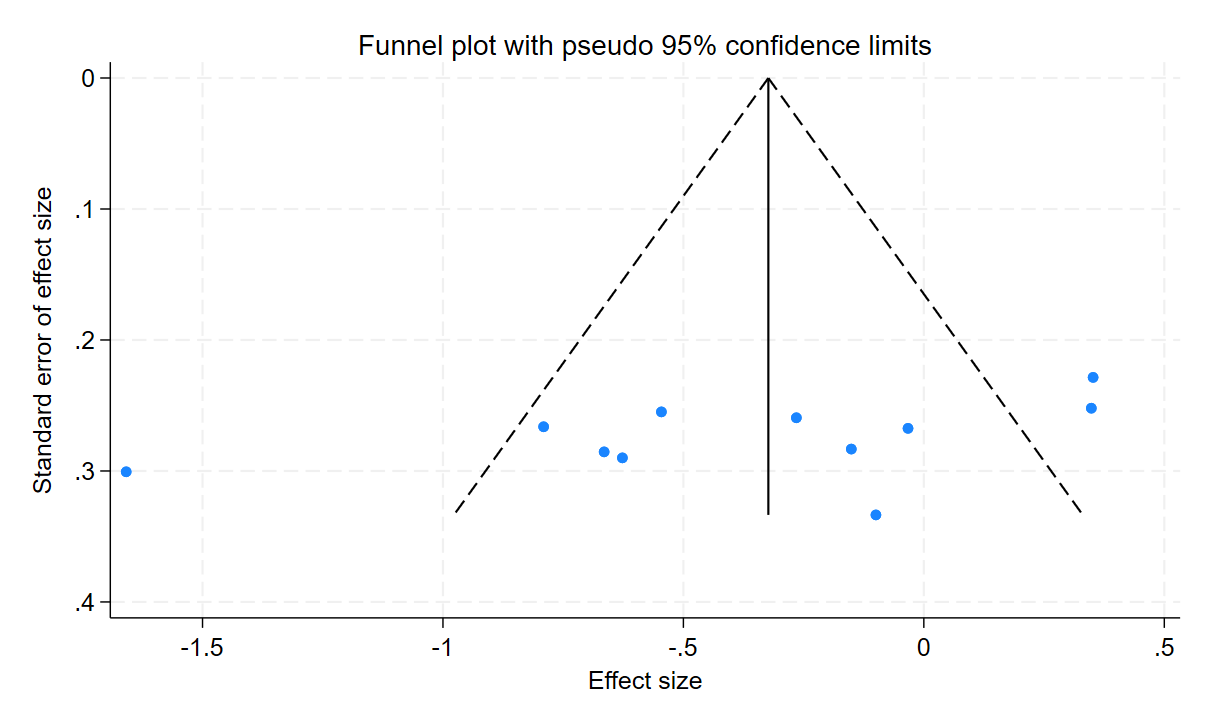


**Fig 2.1 AST-Funnel plot analysis**

**
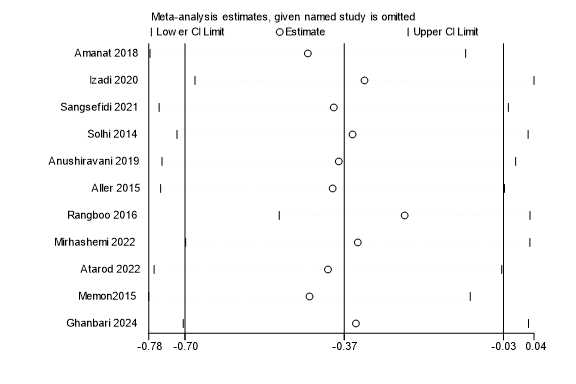
**

**Fig 2.2 AST-Sensitive analysis**

**3 Alkaline Phosphatase(ALP)**

**
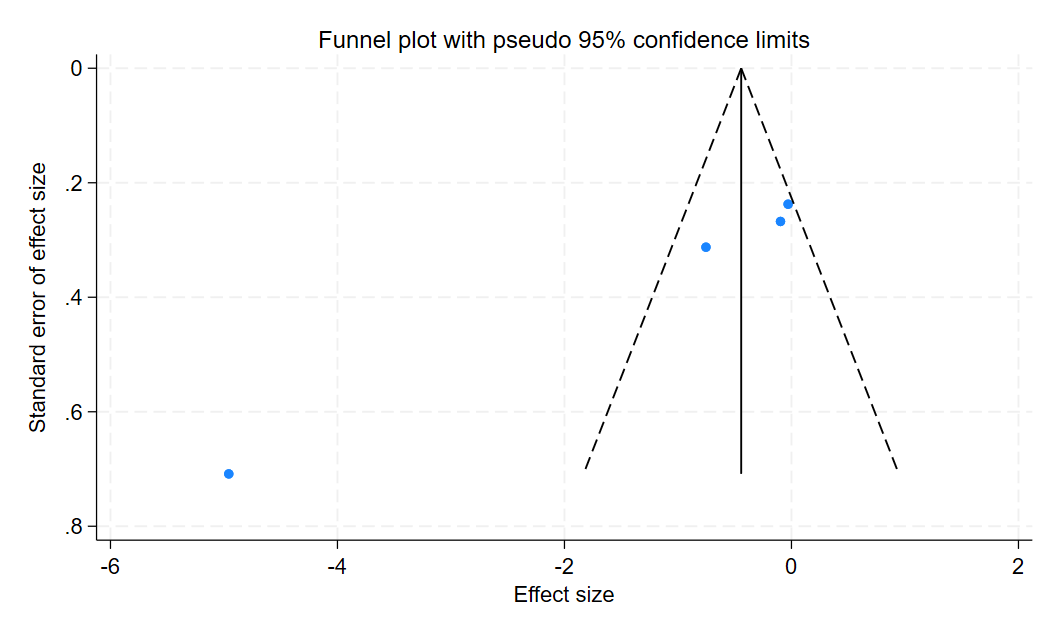
**

**Fig 3.1 ALP-Funnel plot analysis**

**
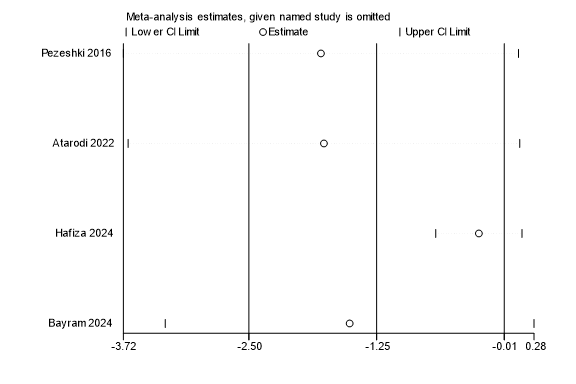
**

**Fig 3.2 ALP-Sensitive analysis**

**4 G-Glutamyl-Transferase(GGT)**

**
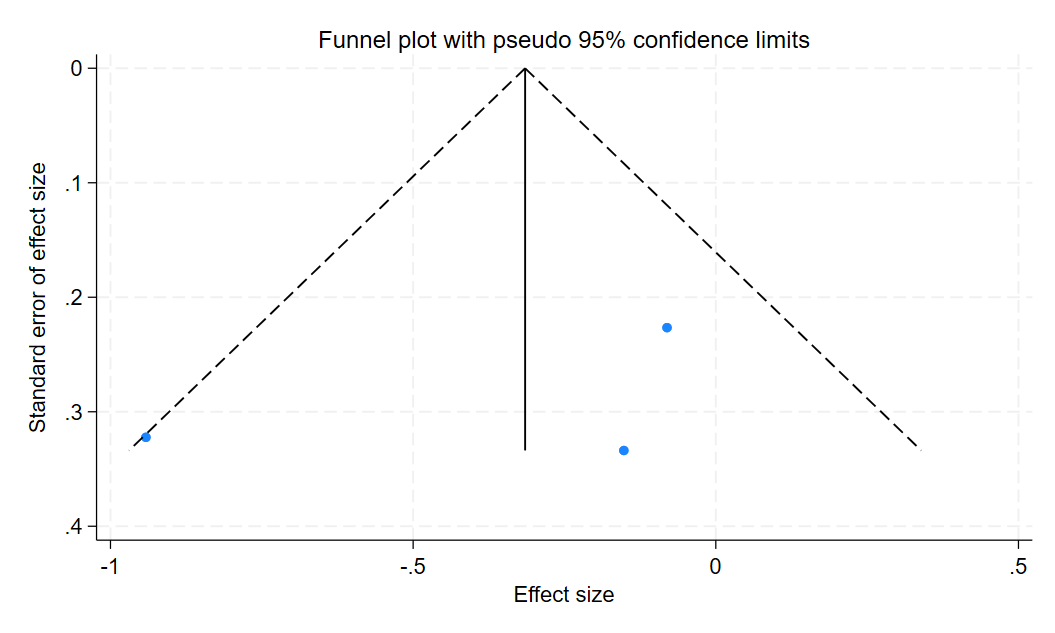
**

**Fig 4.1 GGT-Funnel plot analysis**

**
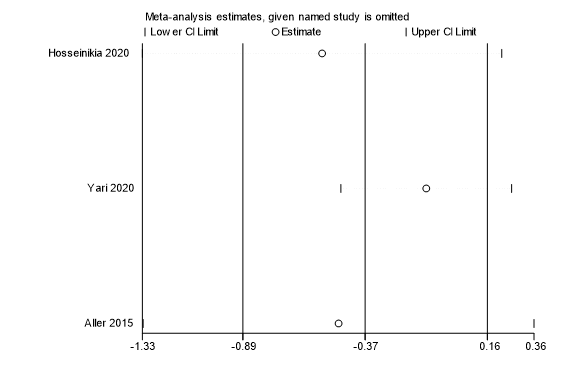
**

**Fig 4.2 GGT-Sensitive analysis**

**5 Steatosis score**

**
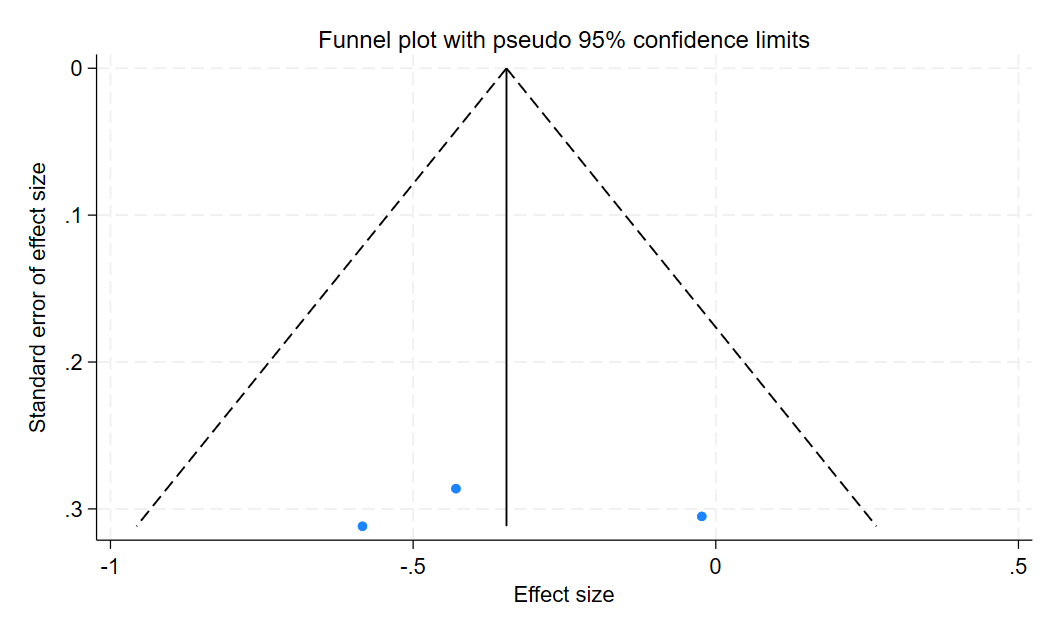
**

**Fig 5.1 Steatosis Score-Funnel plot analysis**

**
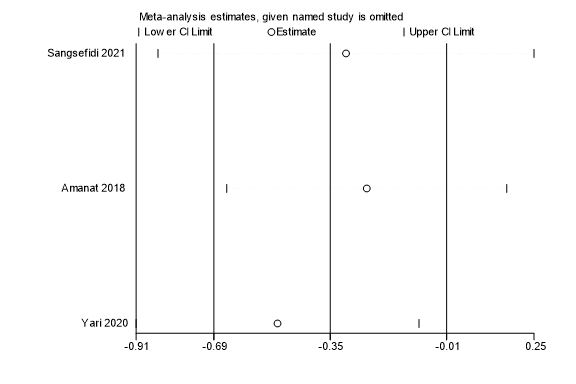
**

**Fig 5.2 Steatosis Score-Sensitive analysis**

**6 Fibrosis score**

**
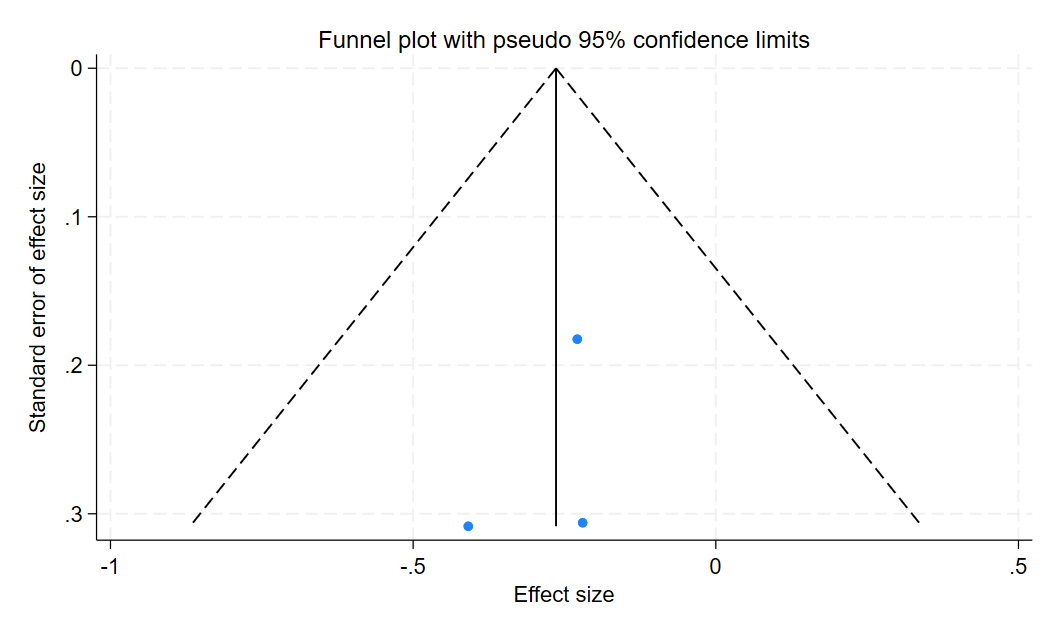
**

**Fig 6.1 Fibrosis Score-Funnel plot analysis**

**
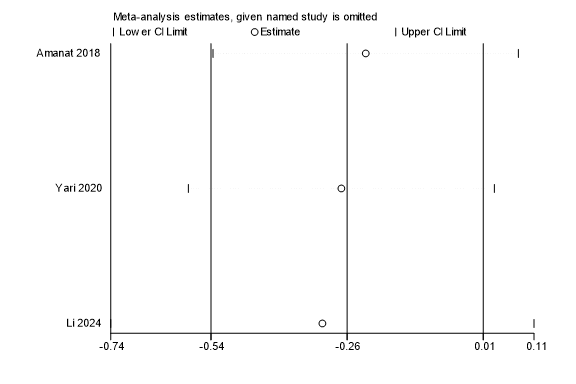
**

**Fig 6.2 Fibrosis Score-Sensitive analysis**

**7 Body Mass Index(BMI)**

**
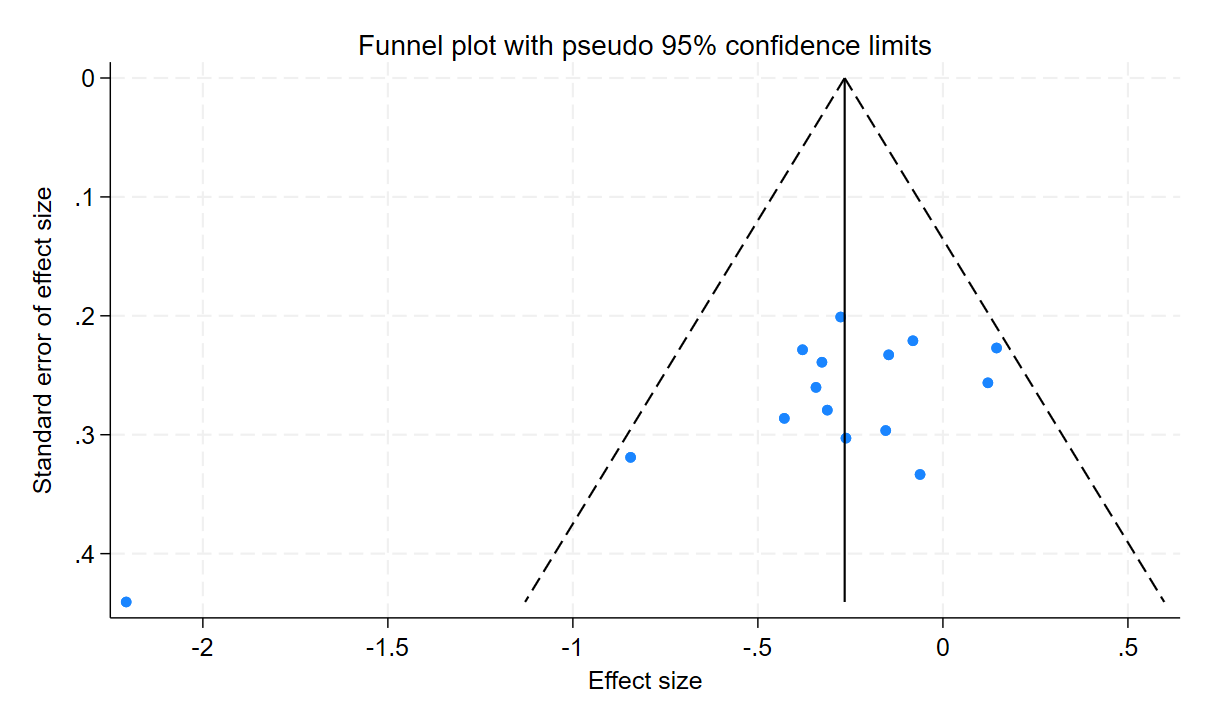
**

**Fig 7.1 BMI-Funnel plot analysis**


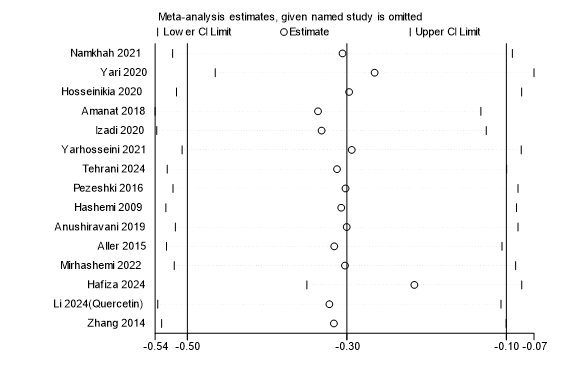


**Fig 7.2 BMI-Sensitive analysis**

8 Waist Circumference(WC)


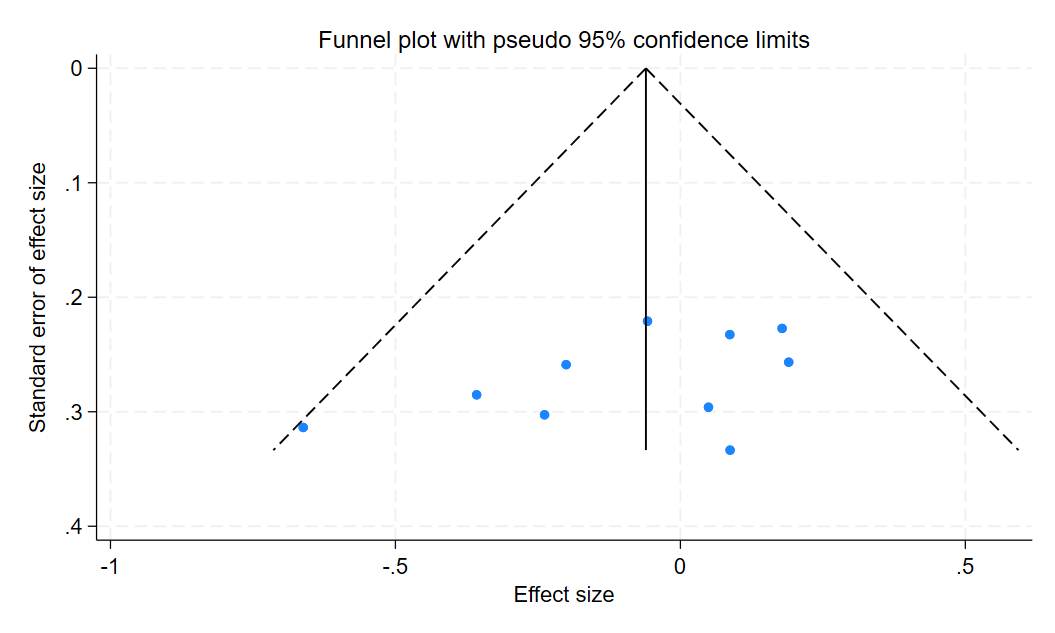


**Fig 8.1** WC-Funnel plot analysis


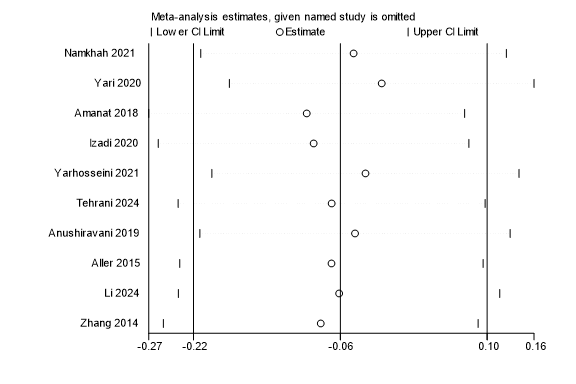


**Fig** 8.2 WC-Sensitive analysis

9 Hip Circumference(HC)


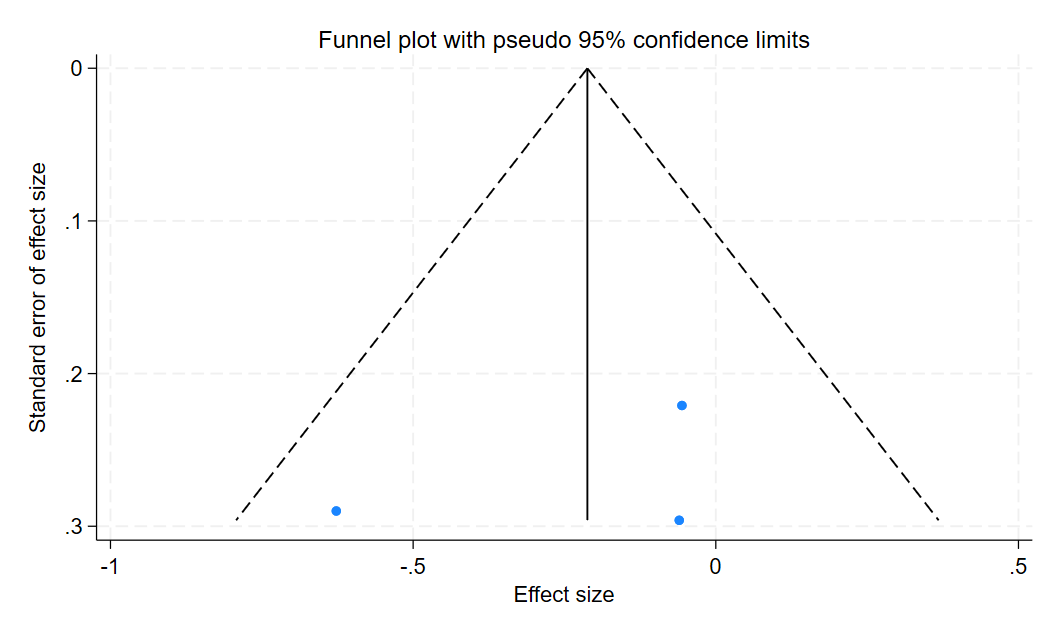


**Fig** 9.1 HC-Funnel plot analysis


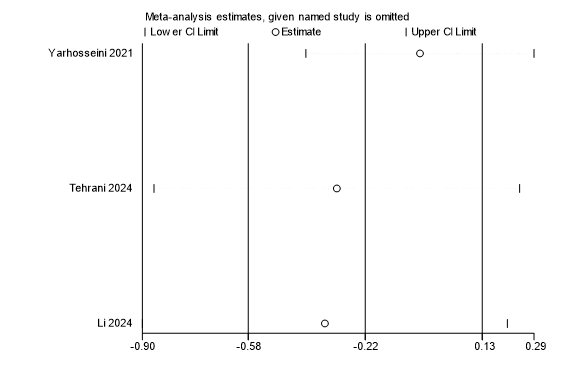


**Fig** 9.2 HC-Sensitive analysis

10 Waist-to-Hip Ratio(WHR)


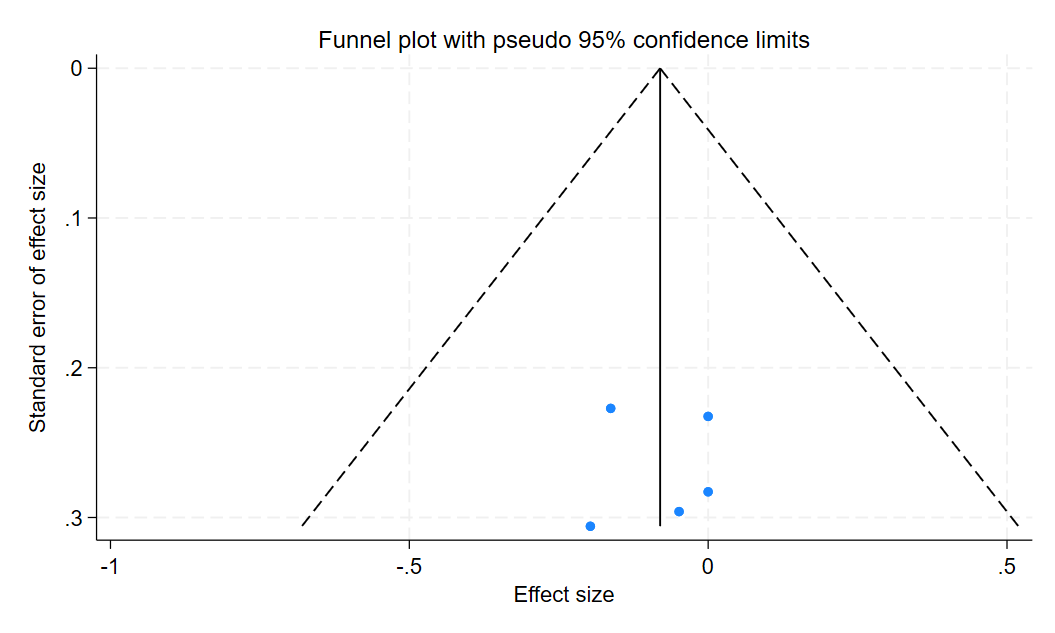


**Fig** 10.1 WHR (Waist-to-Hip Ratio)-Funnel plot analysis


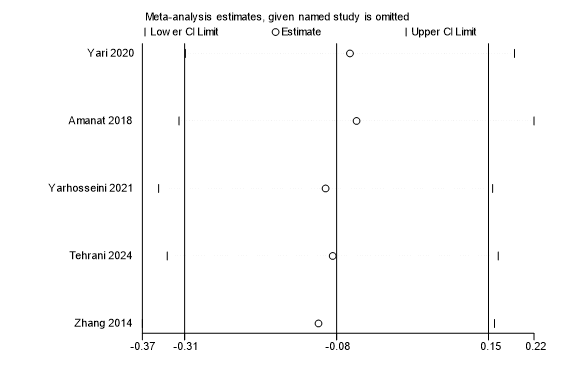


**Fig** 10.2 WHR (Waist-to-Hip Ratio)-Sensitive analysis

11 Weight(WT)


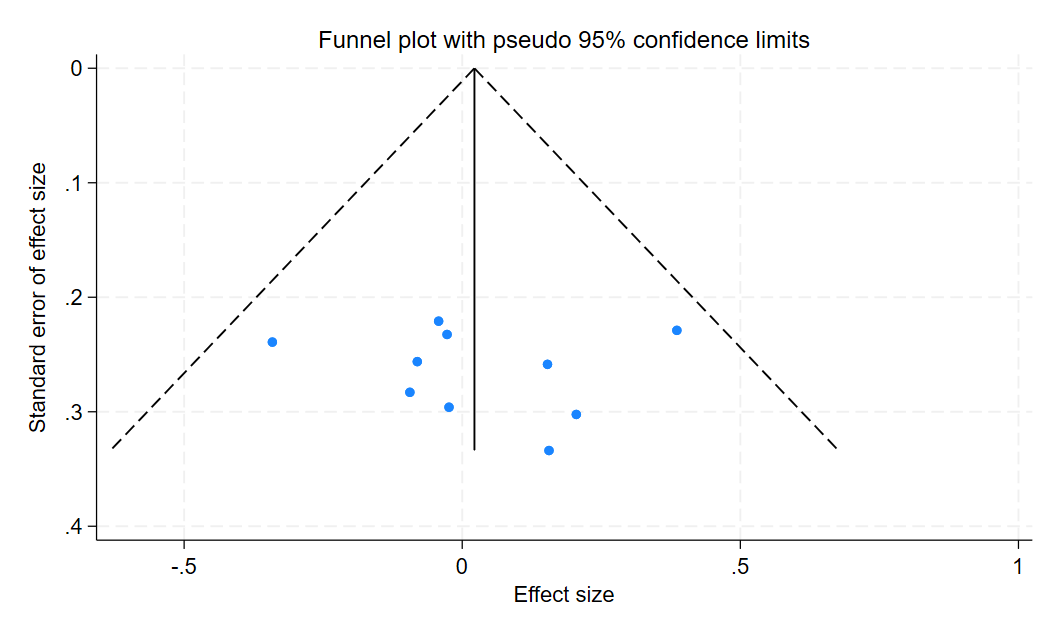


**Fig** 11.1 Weight-Funnel plot analysis


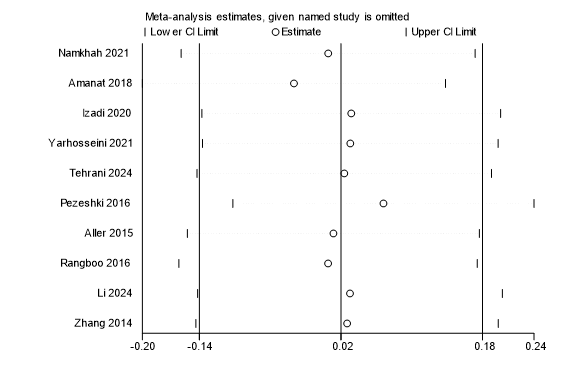


**Fig** 11.2 Weight-Sensitive analysis

12 Fasting Blood Sugar(FBS)


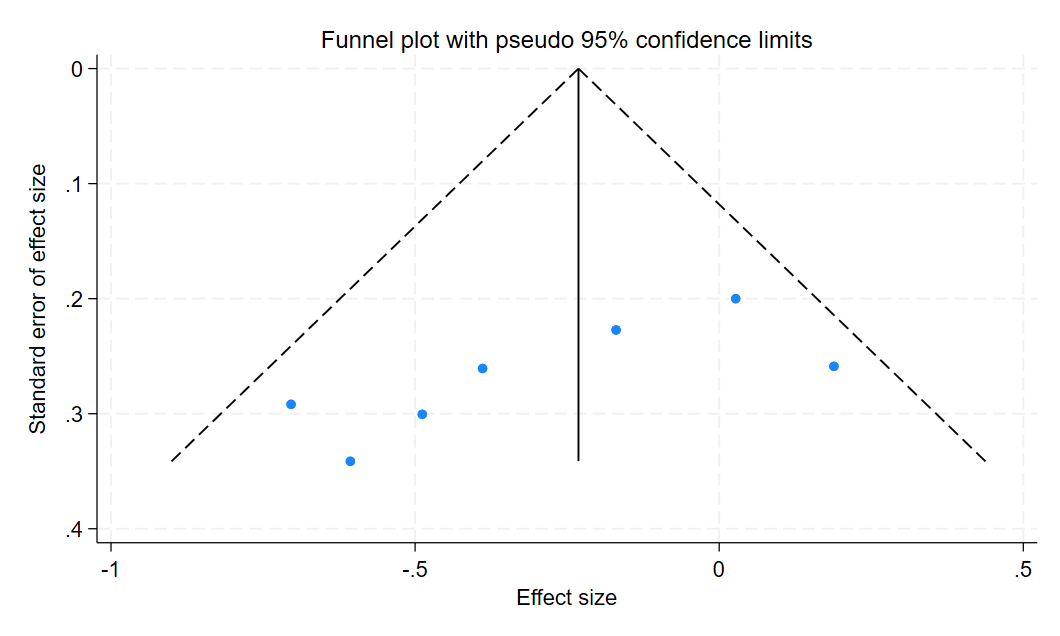
**Fig** 12.1 FBS-Funnel plot analysis


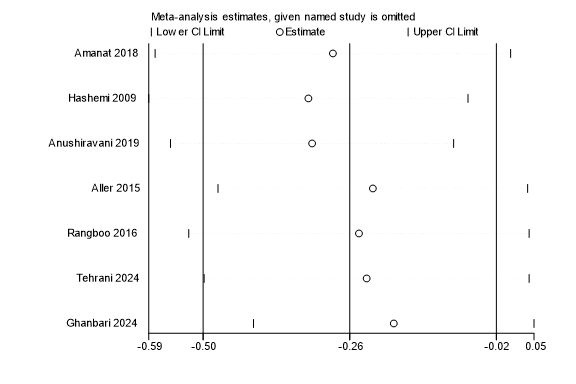


**Fig** 12.2 FBS-Sensitive analysis

13 Homeostatic Model Assessment of Insulin Resistance(HOMA-IR)


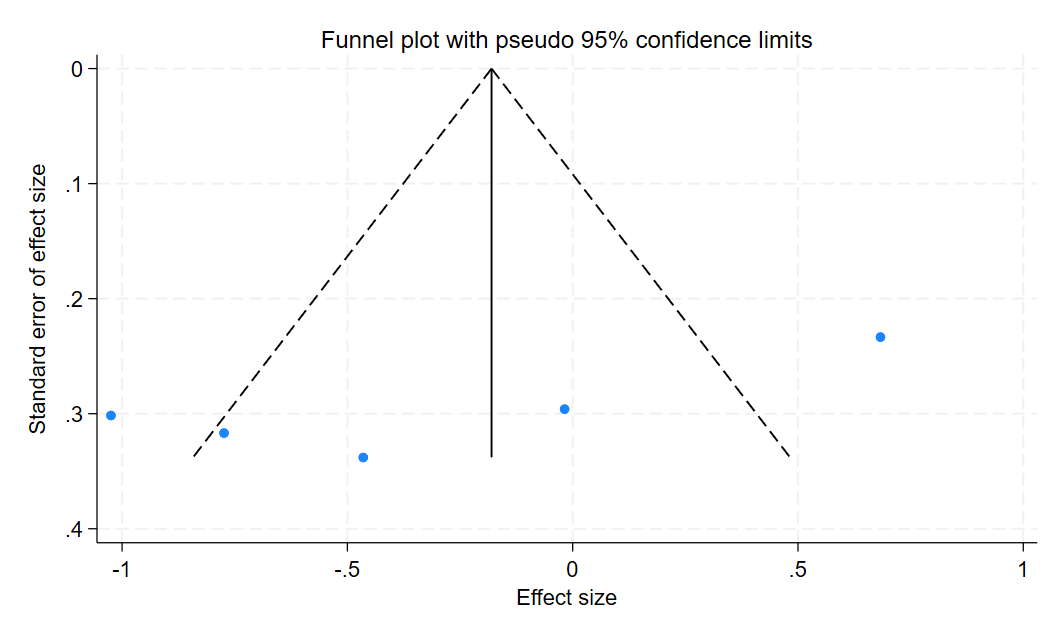


**Fig** 13.1 HOMA-IR-plot analysis


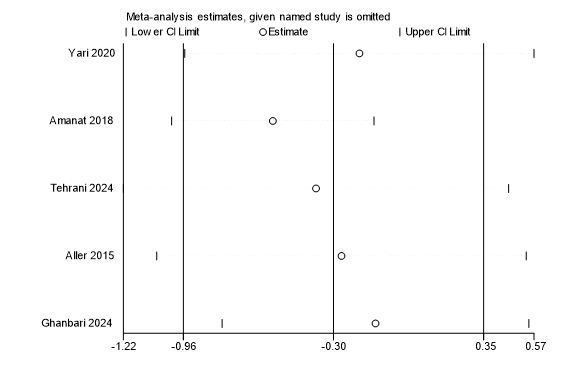


**Fig** 13.2 HOMA-IR-Sensitive analysis

14 Quantitative Insulin Sensitivity Check Index(QUICKI)


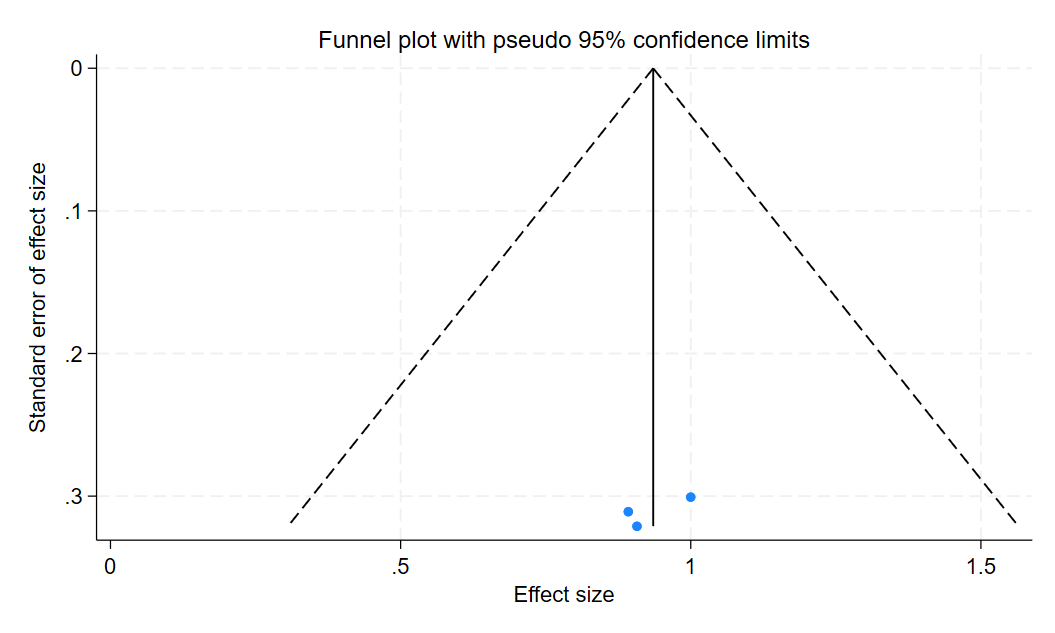


**Fig** 14.1 QUICKI-Funnel plot analysis


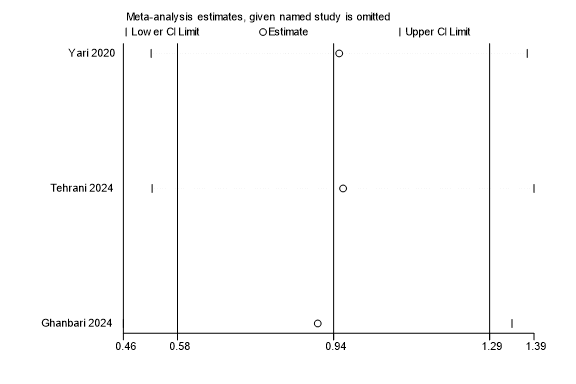


**Fig** 14.2 QUICKI-Sensitive analysis

15 Insulin


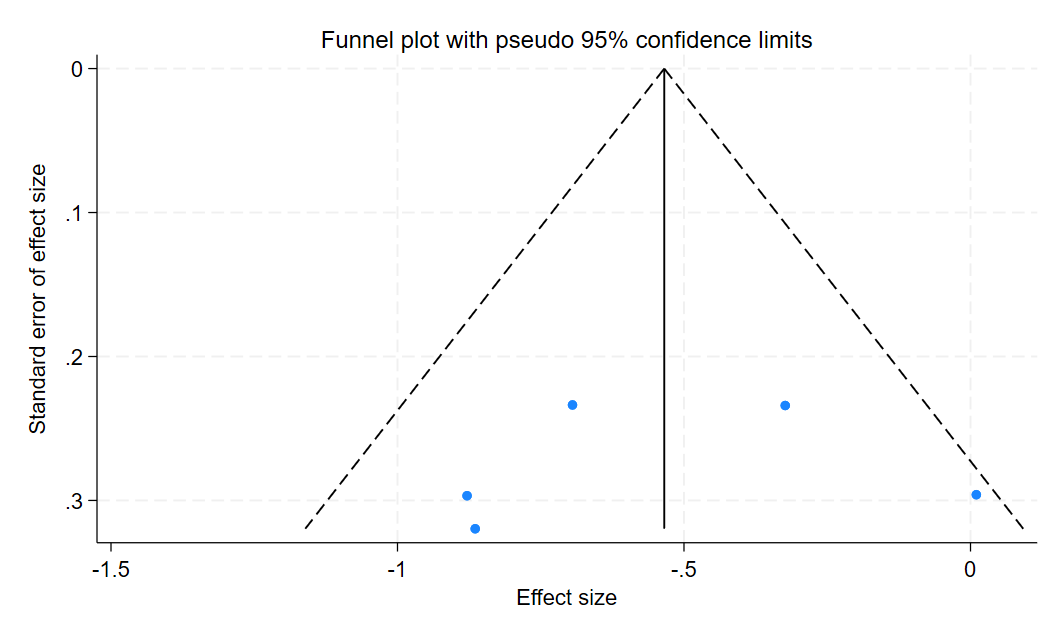


**Fig** 15.1 Insulin-Funnel plot analysis


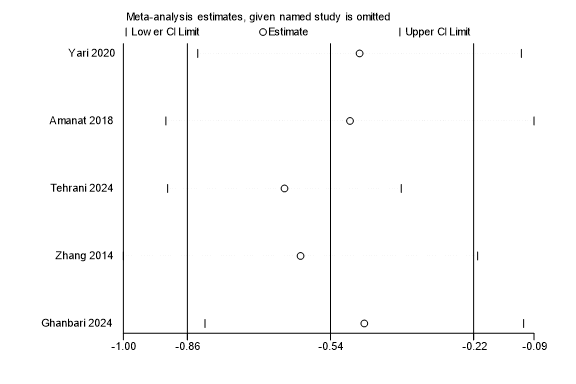


**Fig** 15.2 Insulin-Sensitive analysis

16 **Inflammatory markers**

**
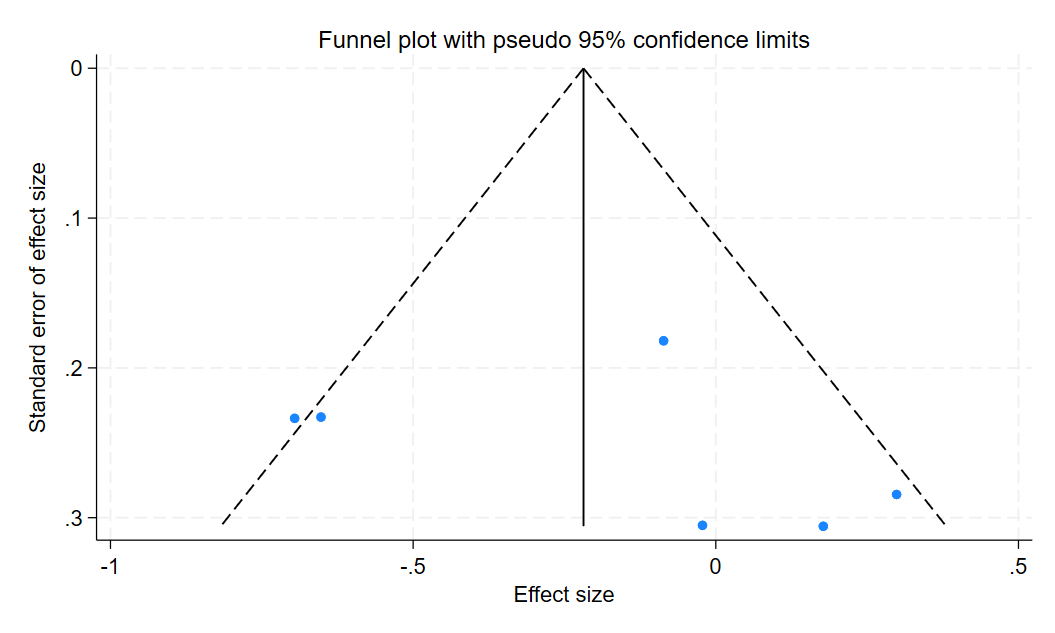
**

**Fig** 16.1 Inflammatory markers-Funnel plot analysis


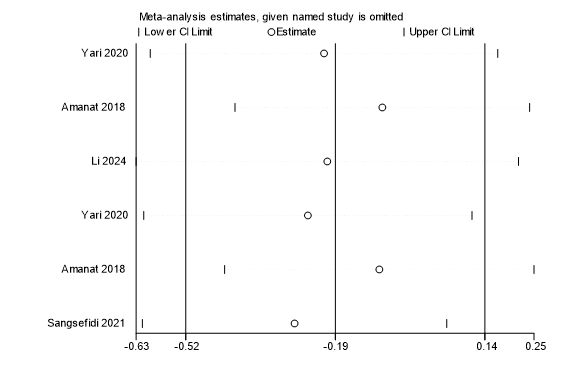


**Fig** 16.2 Inflammatory markers-Sensitive analysis

17 Total Cholesterol(TC)


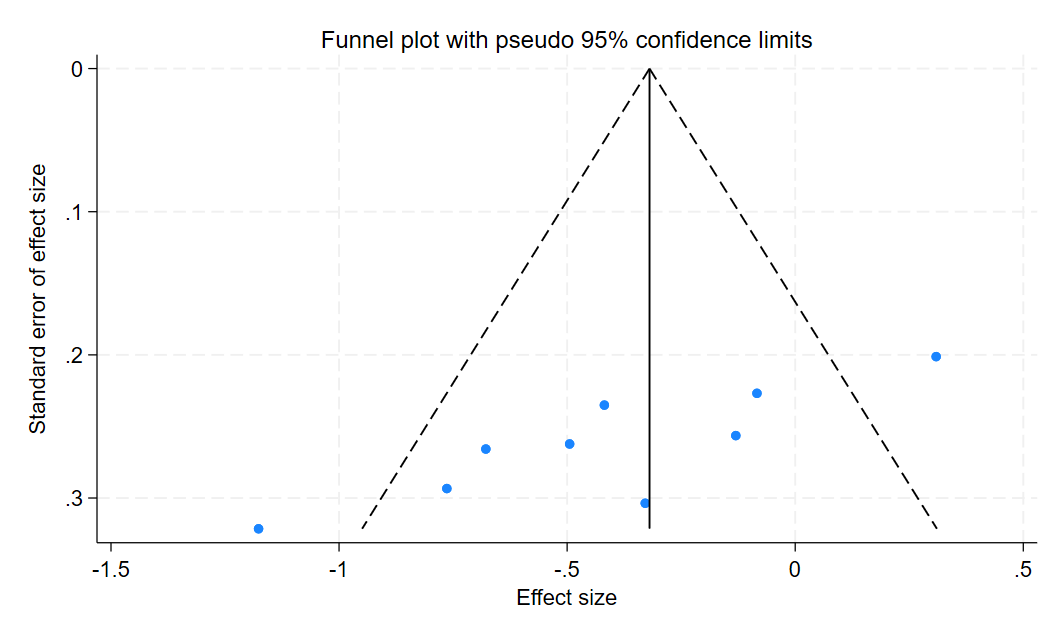


**Fig** 17.1 TC-Funnel plot analysis


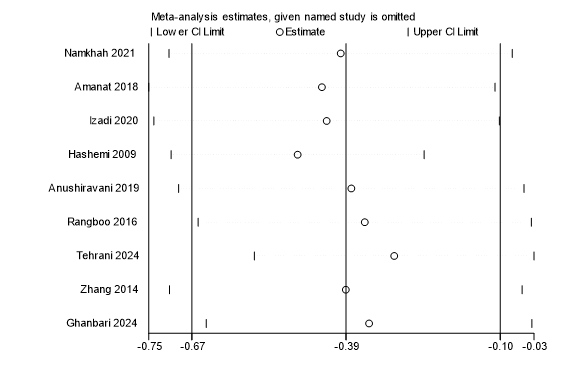


**Fig** 17.2 TC-Sensitive analysis

18 Triglycerides(TG)


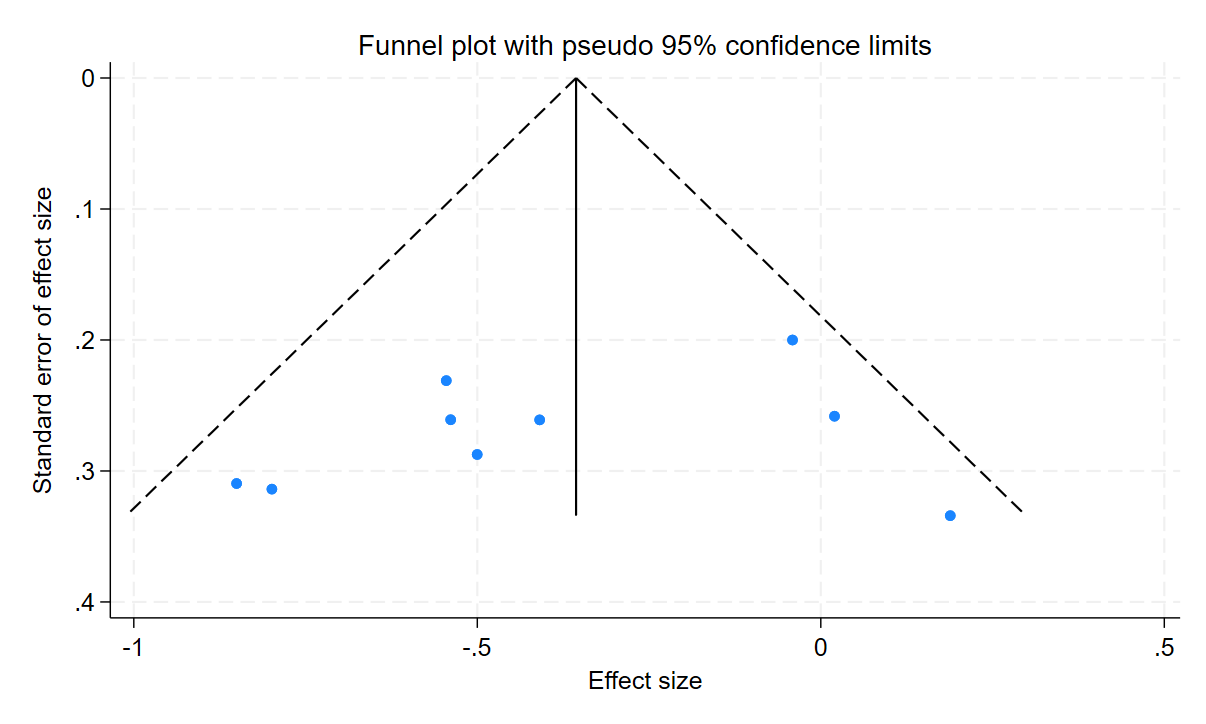


**Fig** 18.1 TG-Funnel plot analysis


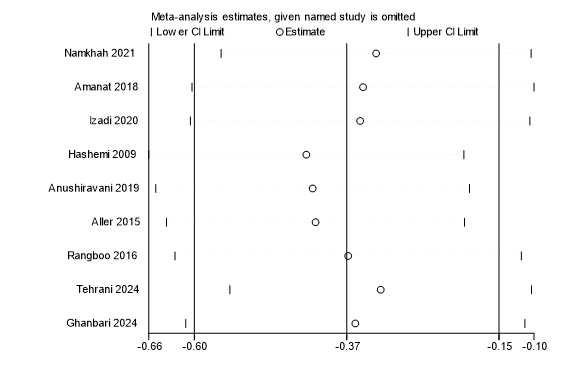


**Fig** 18.2 TG-Sensitive analysis

19 High-Density Lipoprotein Cholesterol(HDL-C)


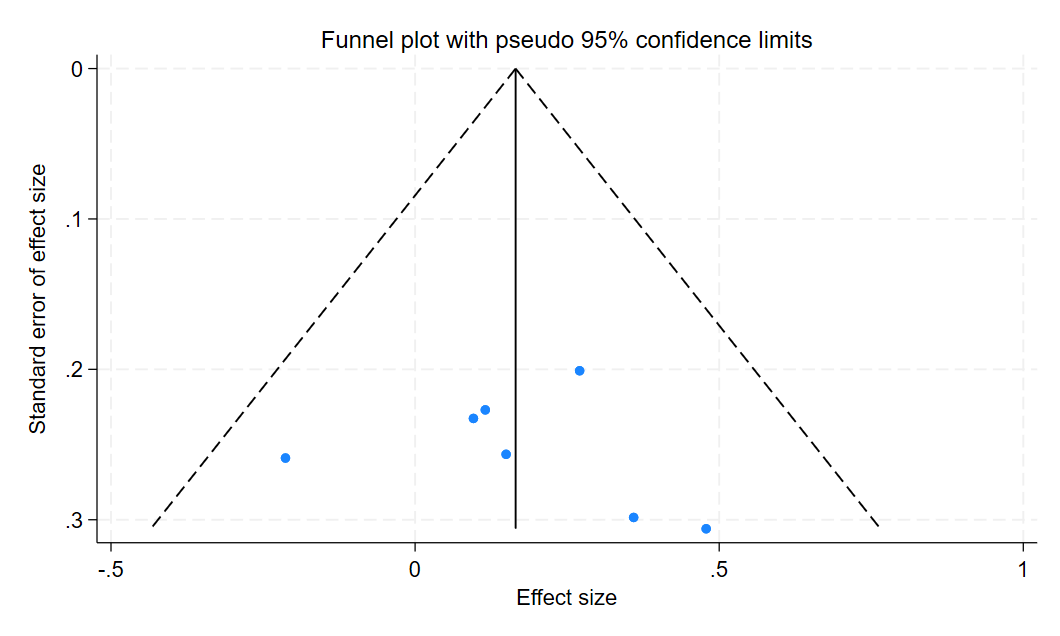


**Fig** 19.1 HDL-C-Funnel plot analysis


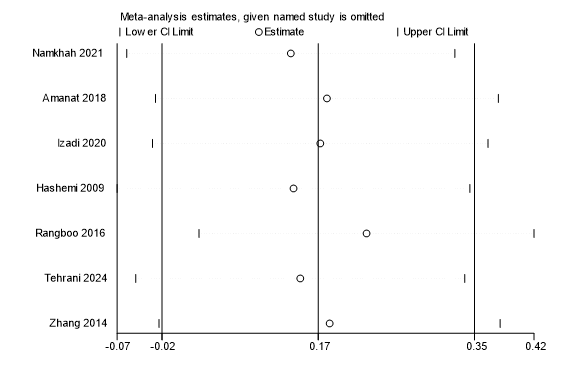


**Fig** 19.2 HDL-C-Sensitive analysis

20 Low-Density lipo-Protein Cholesterol(LDL-C)


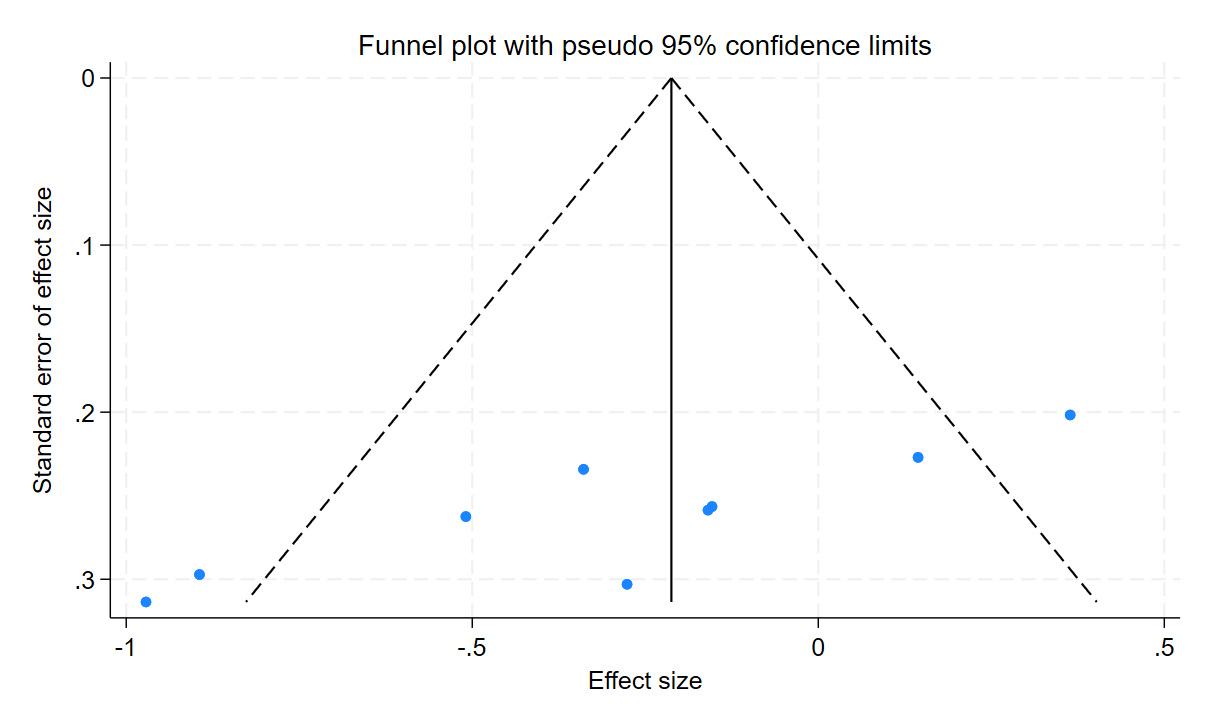


**Fig** 20.1 LDL-C-Funnel plot analysis


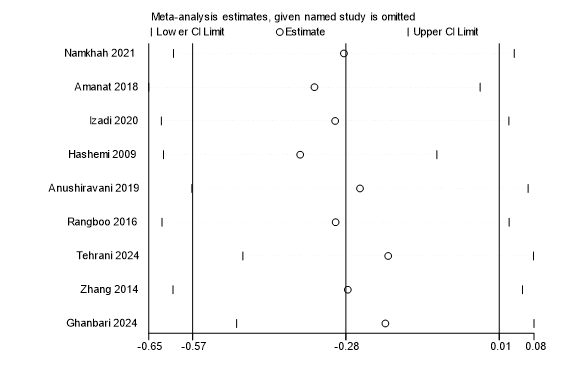


**Fig** 20.2 LDL-C-Sensitive analysis

Appendix A3

1 Alanine Aminotransferase（ALT)


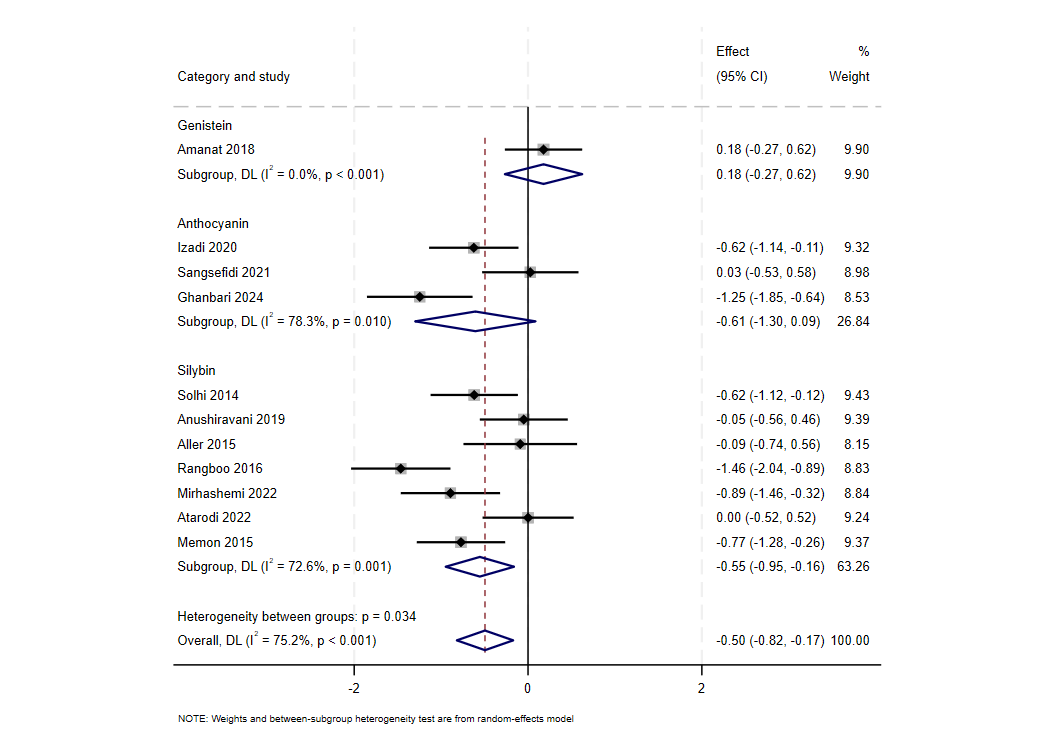


**Fig 1.1** ALT-Category-subgroup analysis


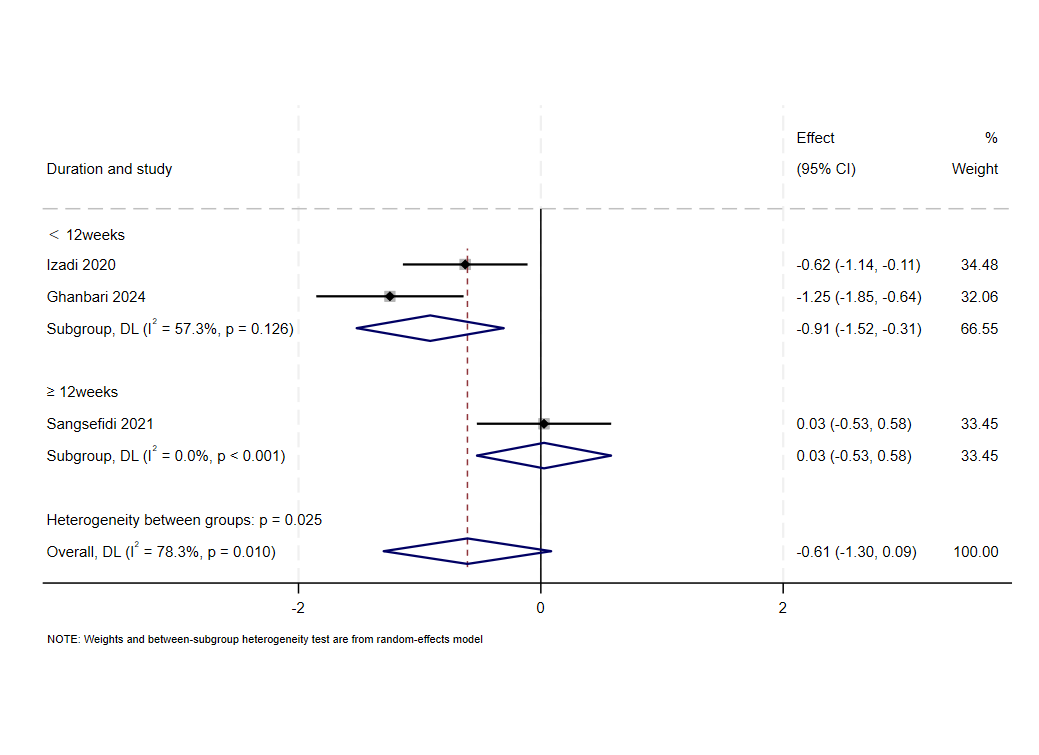


**Fig 1.2** ALT-Anthocyanin-Duration-subgroup


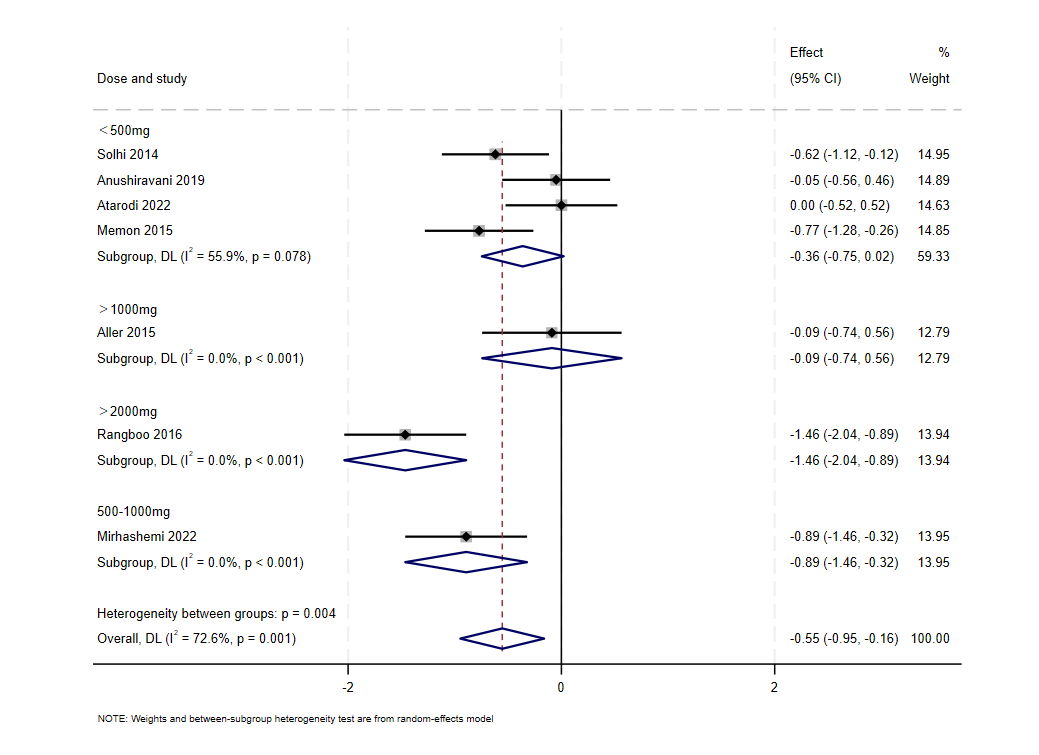


**Fig 1.3** ALT-Silybin-Dose-subgroup analysis

**2 Aspartate Aminotransferase(AST)**


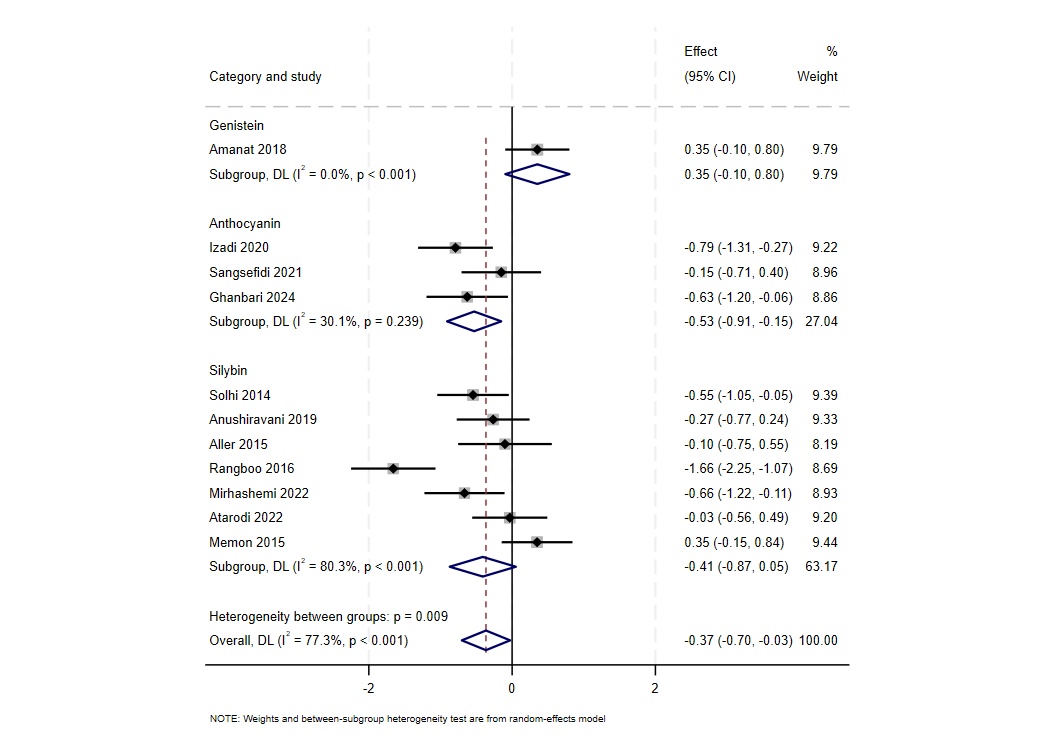


**Fig 2.1** AST-Category-subgroup analysis


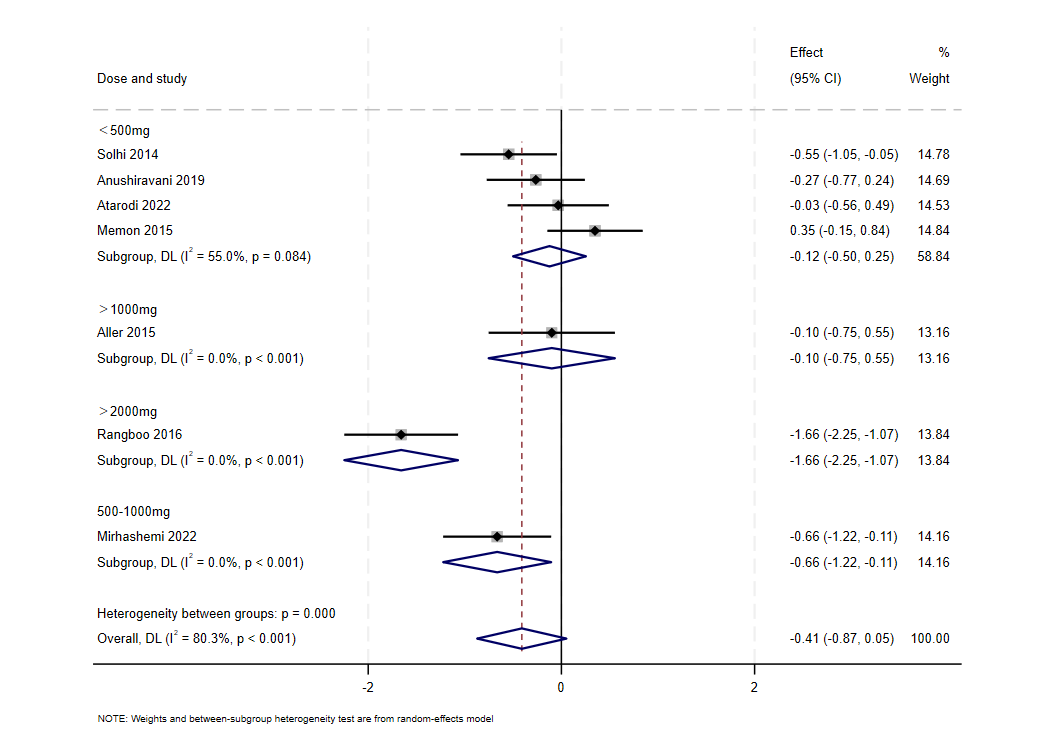


**Fig 2.2** AST-silymarin-Dose-subgroup analysis

**3 Alkaline Phosphatase(ALP)**


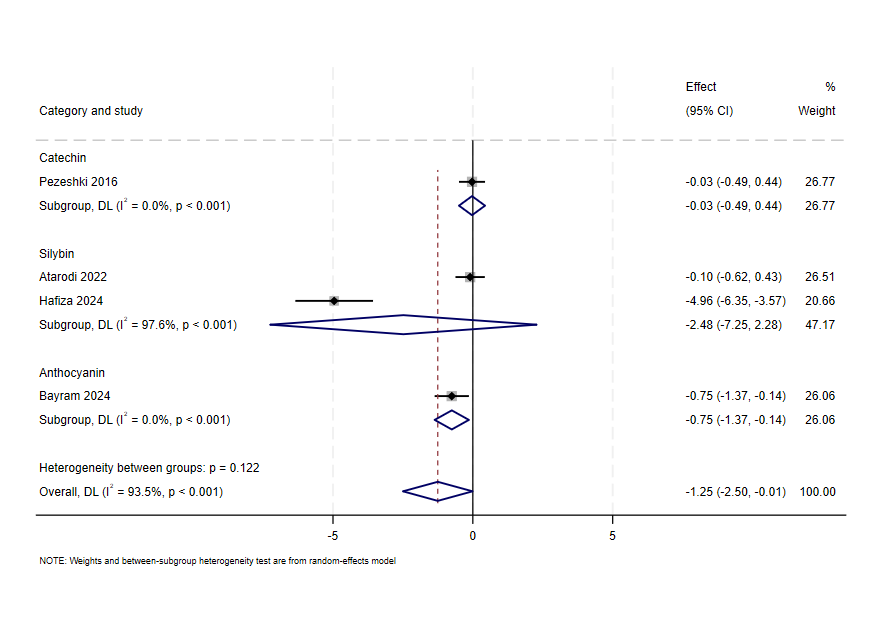


**Fig3.1** ALP-Category-subgroup analysis


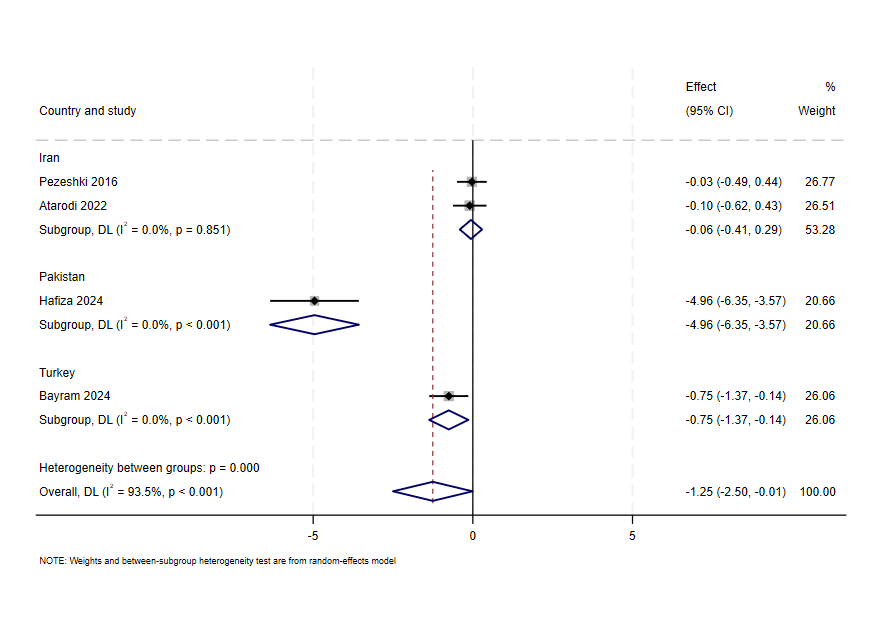


**Fig 3.2 ALP-Country-subgroup analysis**

**4 G-Glutamyl-Transferase(GGT)**

**
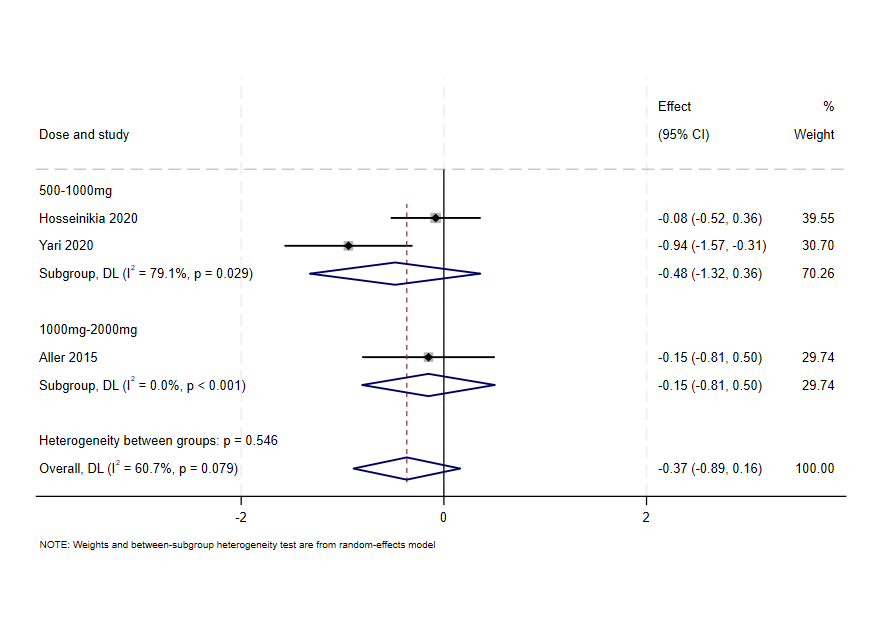
**

**Fig 4 GGT-Dose-subgroup analysis**

**5 Body Mass Index(BMI)**

**
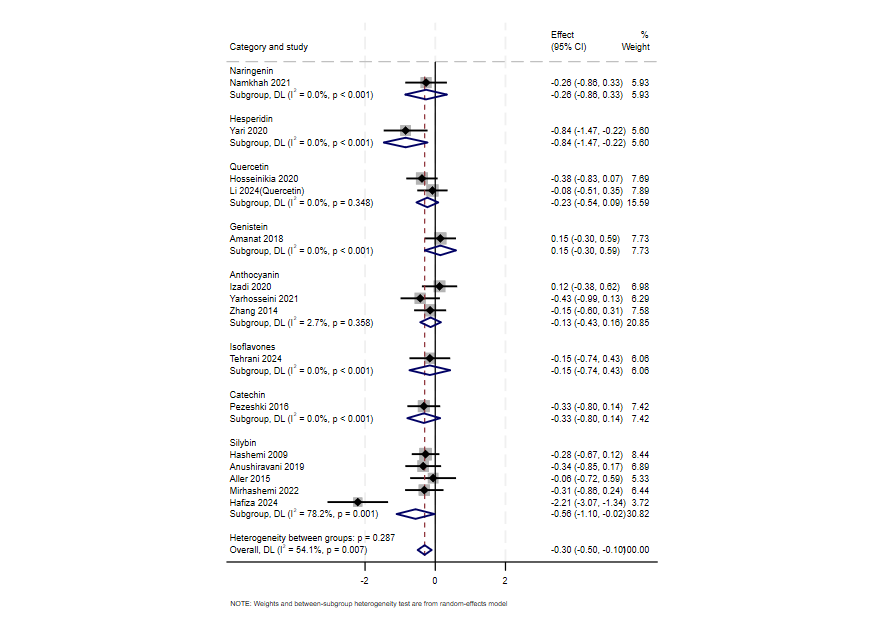
**

**Fig 5.1 BMI-Category-subgroup analysis**

**
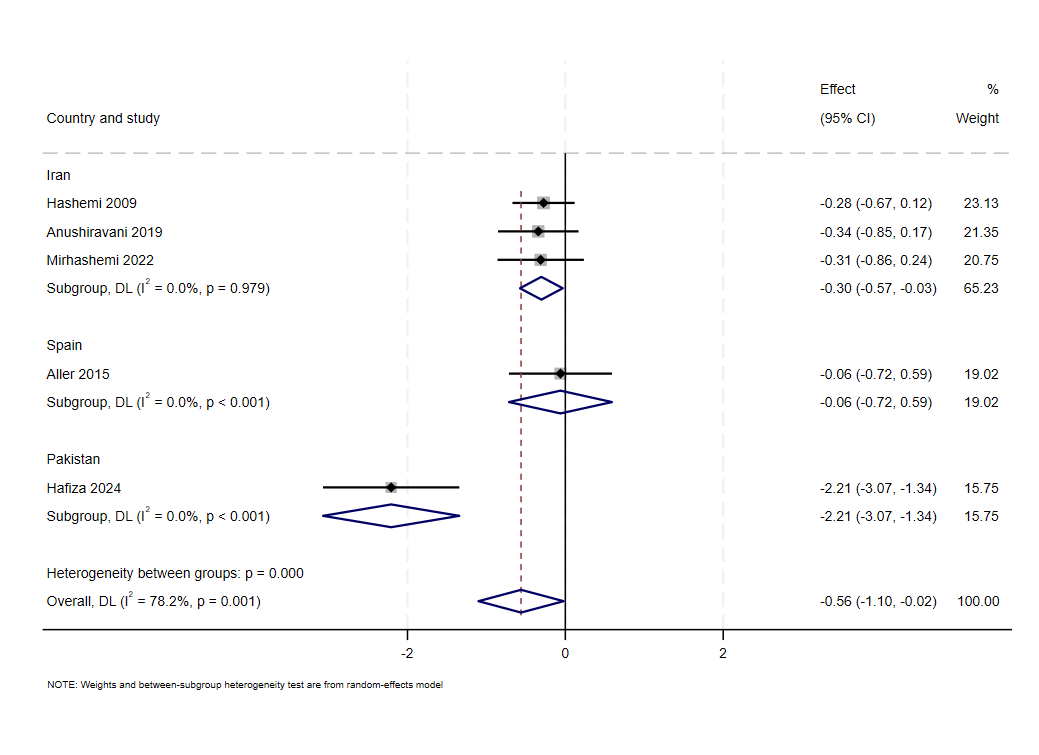
**

**Fig 5.2 BMI-Silybin-Country-subgroup analysis**

6 Waist Circumference(WC)


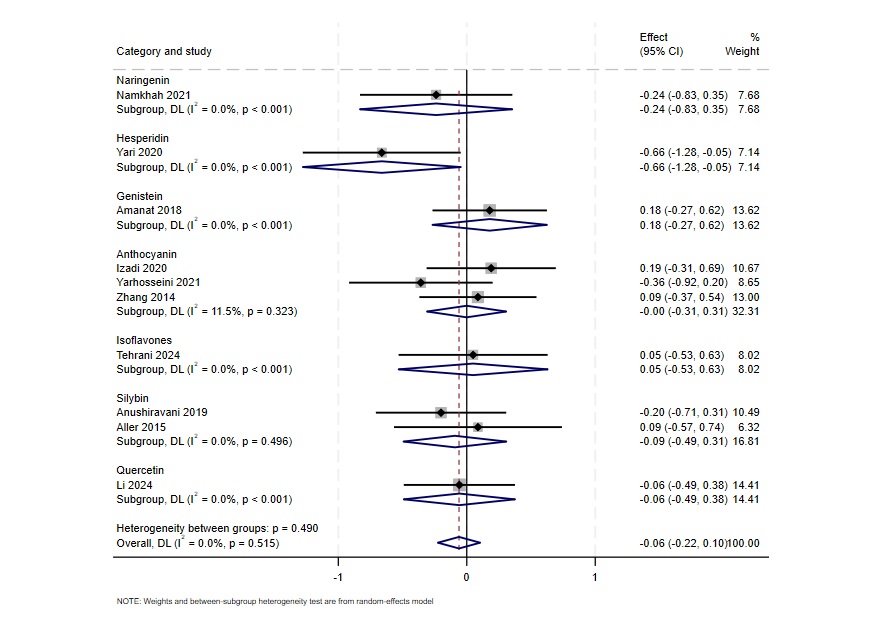


**Fig 6** WC-Category-subgroup analysis

7 Waist-to-Hip Ratio(WHR)


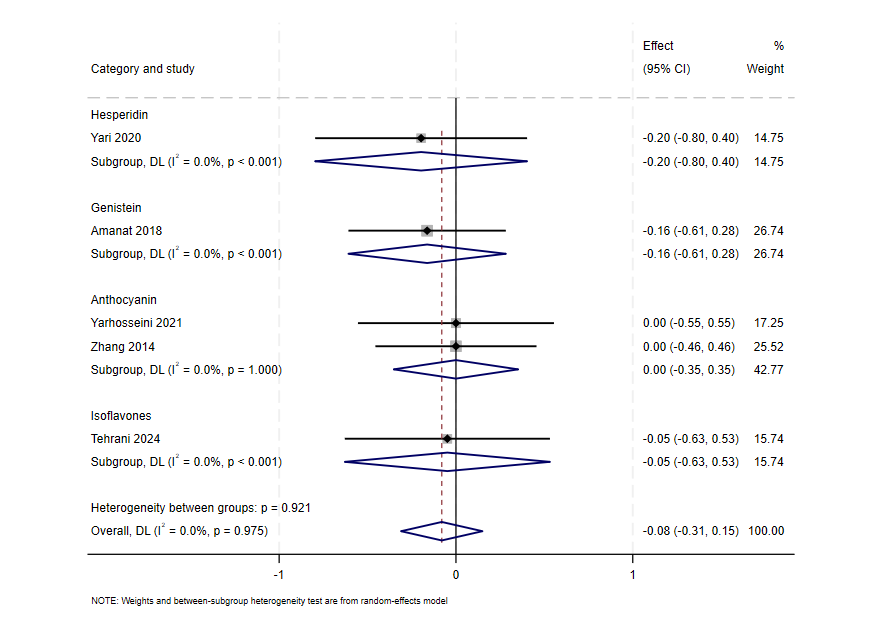


**Fig 7** WHR (Waist-to-Hip Ratio)-Category-subgroup analysis

8 Weight(WT)


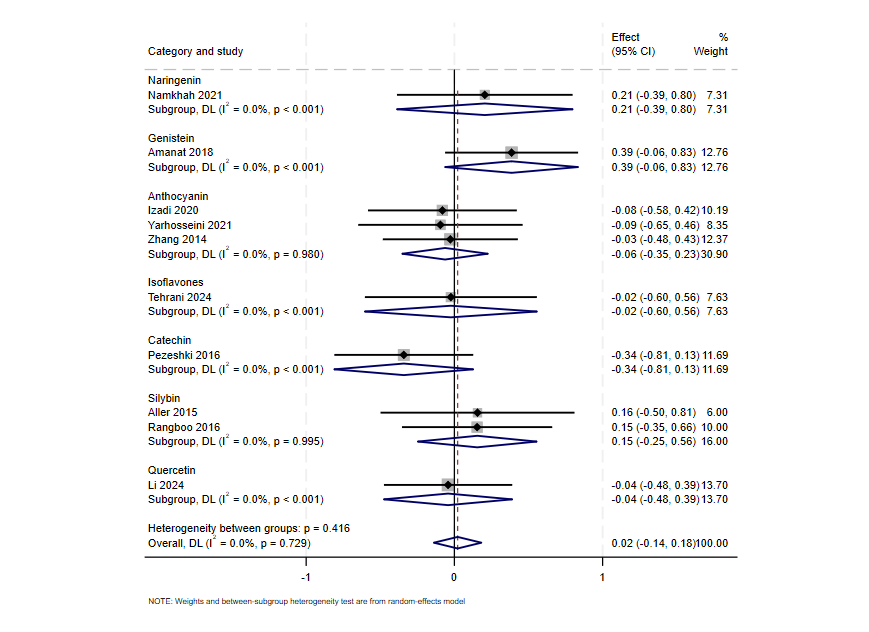


**Fig 8 Weight-Category-subgroup analysis**

**9** Fasting Blood Sugar(FBS)


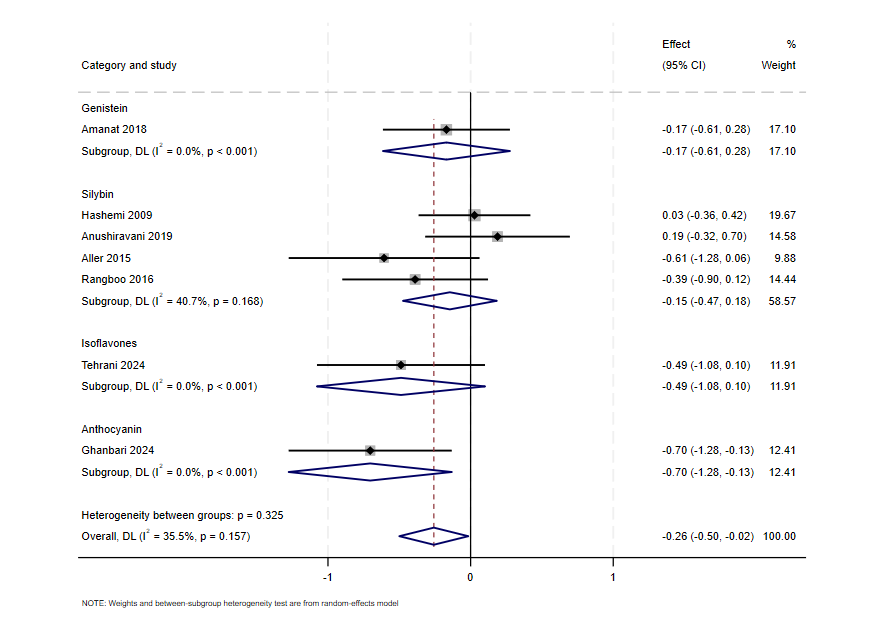


**Fig 9 FBS-Category-subgroup analysis**

10 Insulin


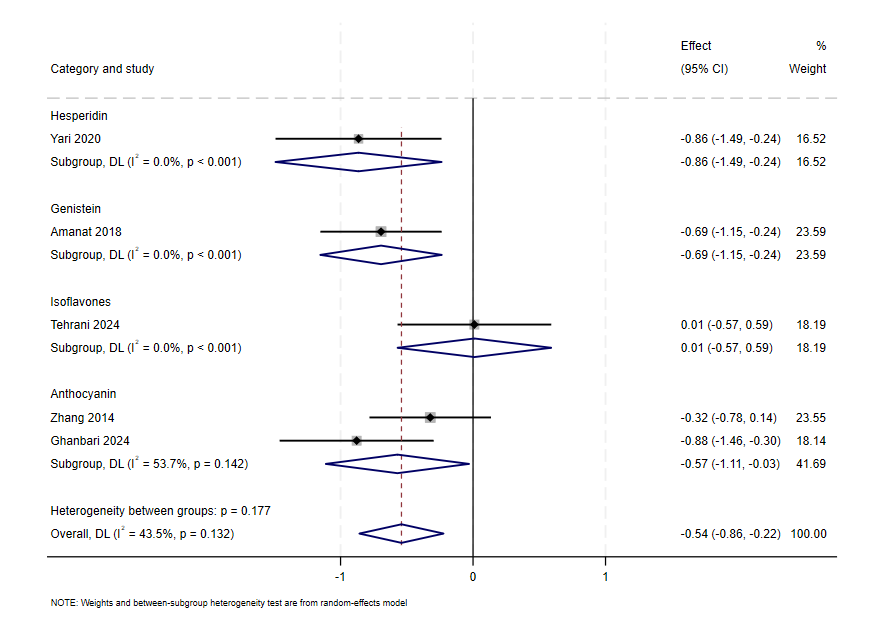


**Fig**10 Insulin-Category-subgroup analysis

**11 Inflammatory markers**


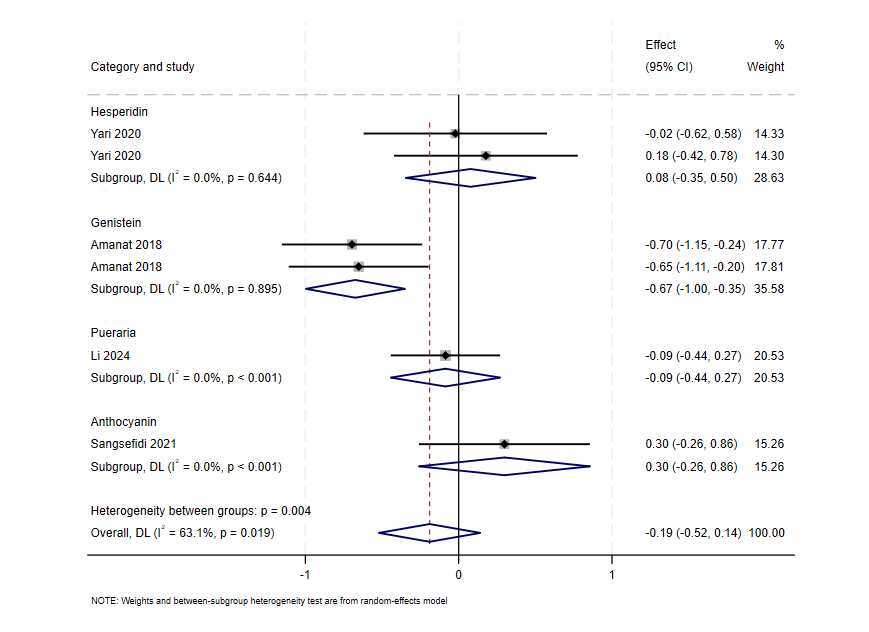


**Fig** 11.1 Inflammatory markers-Category-subgroup analysis


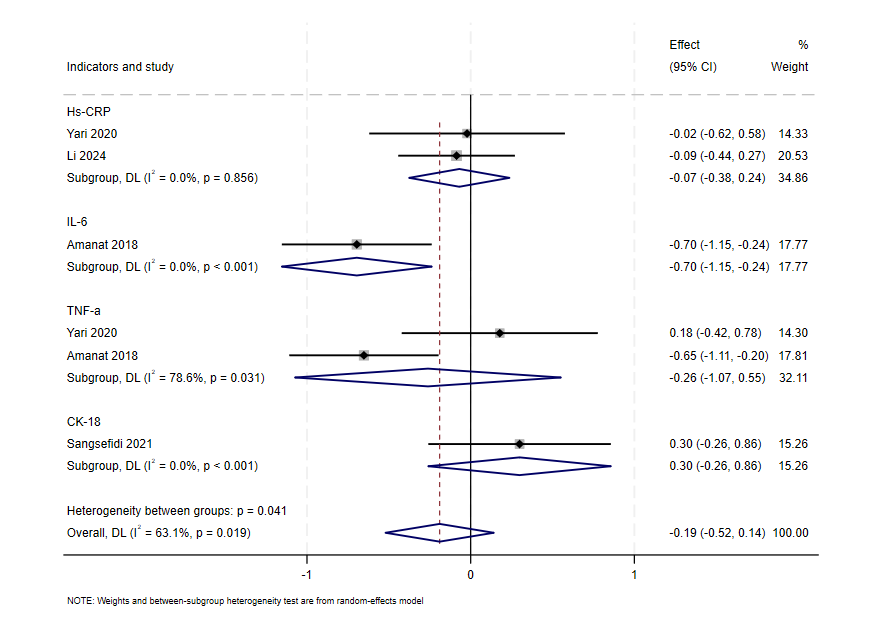


**Fig**11.2 Inflammatory markers-Indicators-subgroup analysis

12 Total Cholesterol(TC)


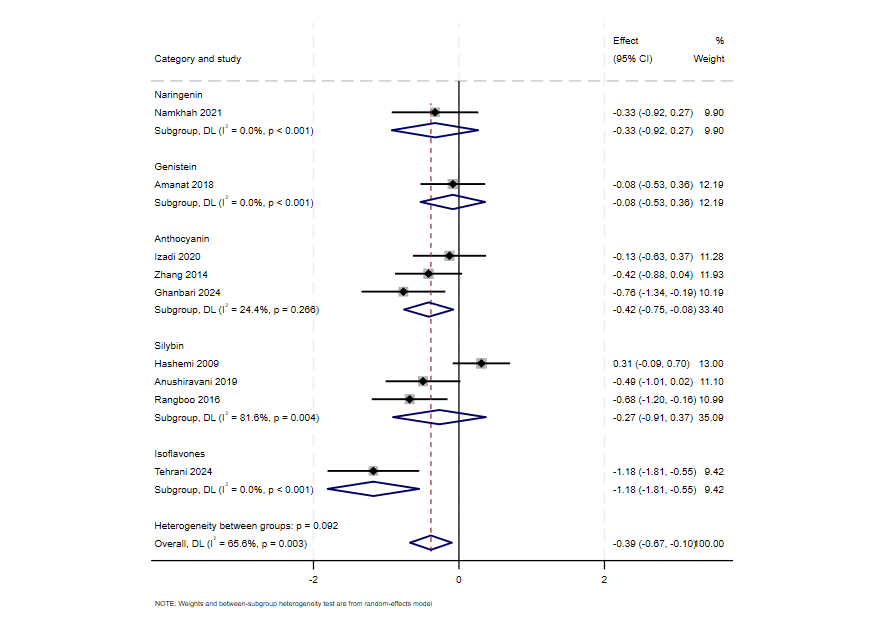


**Fig**12.1 TC-Category-subgroup analysis


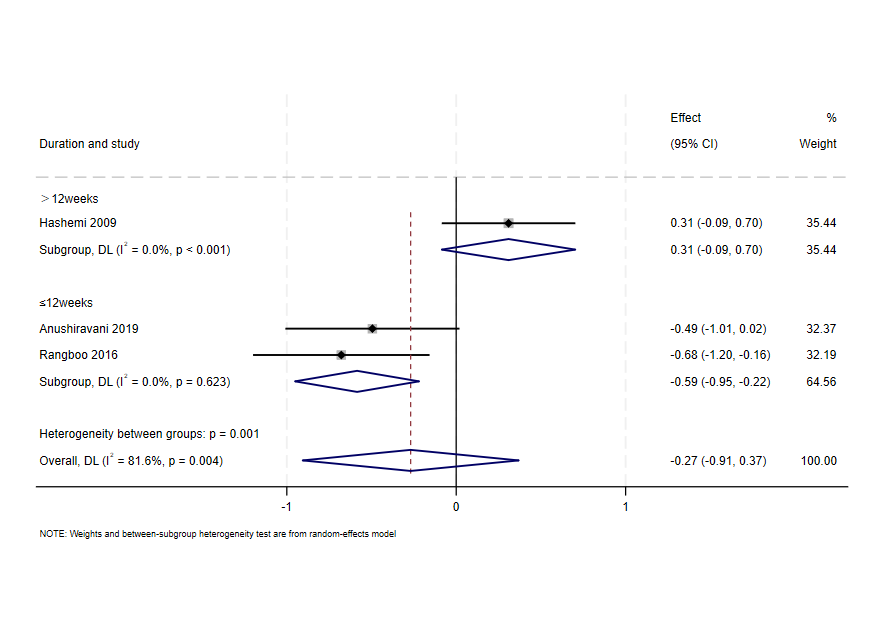


**Fig**12.2 TC-Silybin-Duration-subgroup analysis

13 Triglycerides(TG)


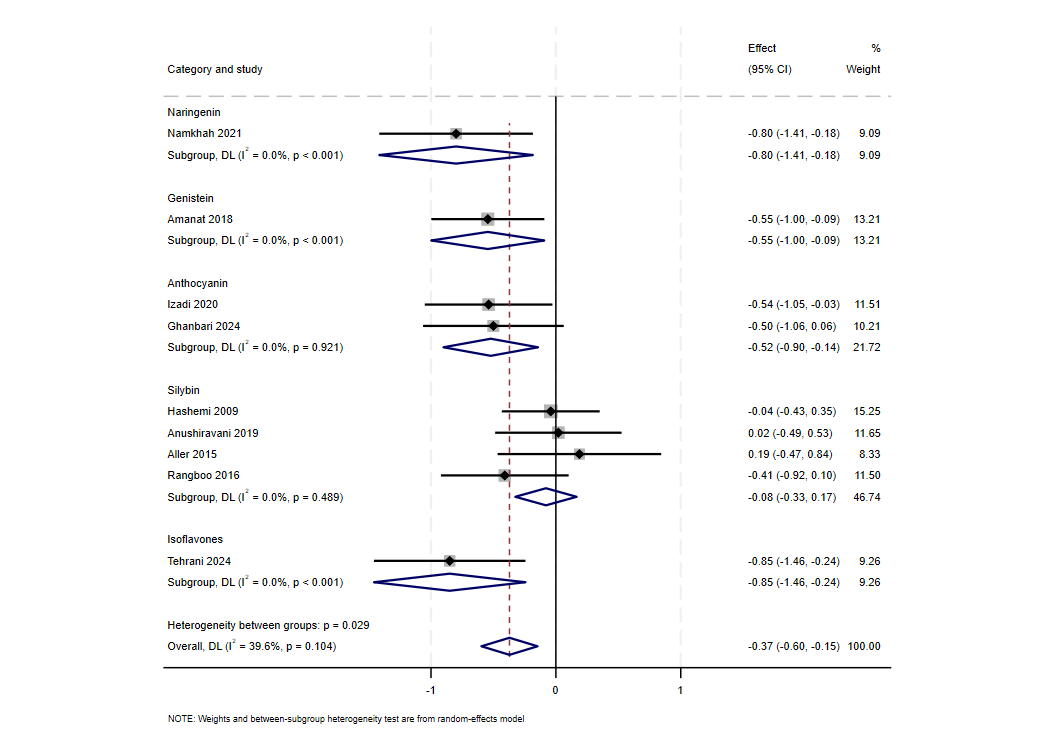


**Fig**13 TG-Category-subgroup analysis

14 High-Density Lipoprotein Cholesterol(HDL-C)


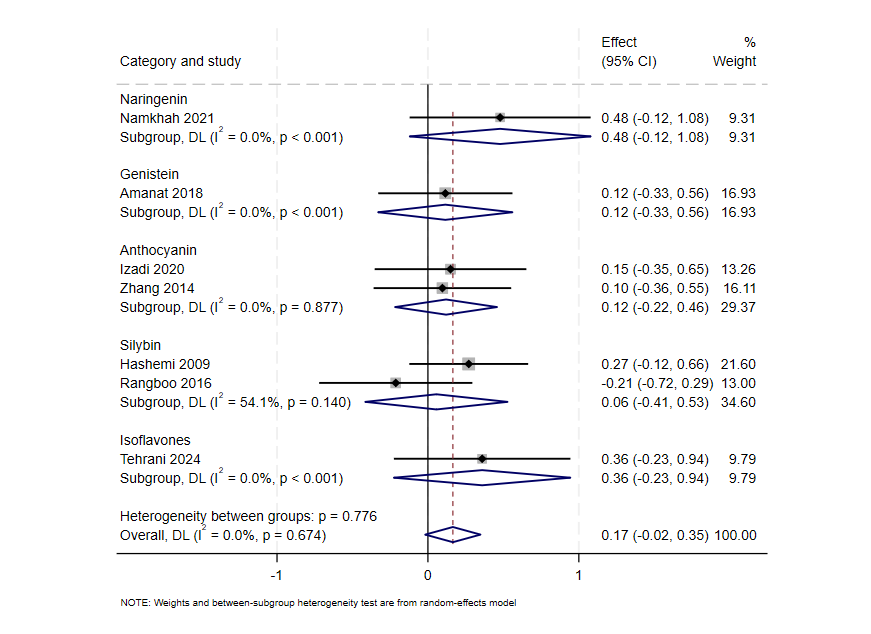


**Fig**14 HDL-C Category-subgroup analysis

15 Low-Density lipo-Protein Cholesterol(LDL-C)


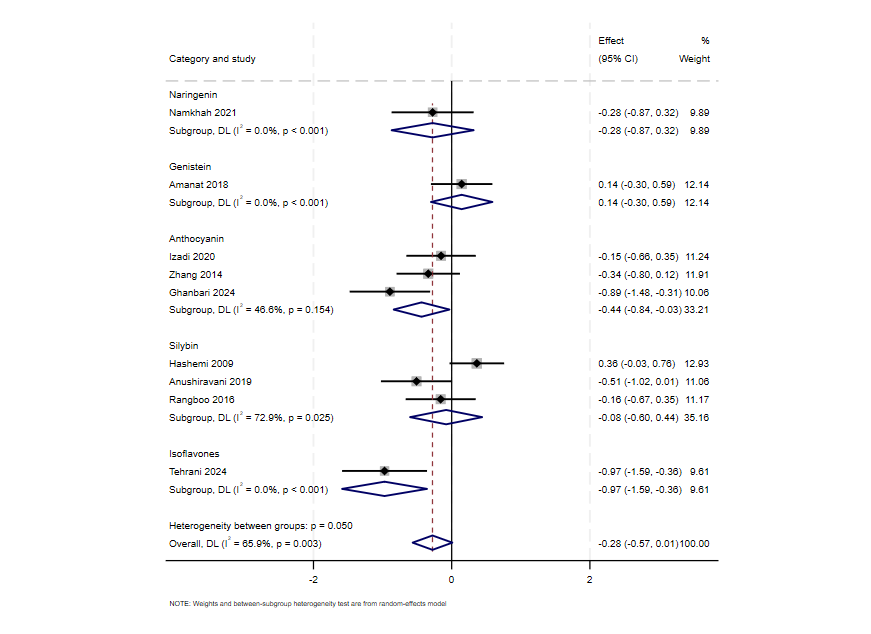


**Fig**15.1 LDL-Category-subgroup analysis


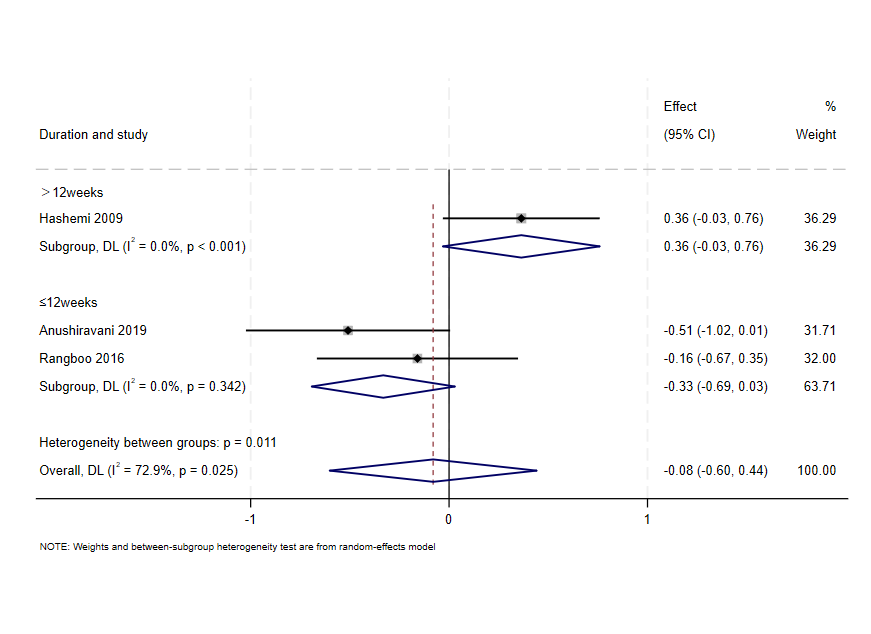


**Fig**15.2 LDL-Silybin-Duration-subgroup analysis

Appendix A4

1 G-Glutamyl-Transferase(GGT)


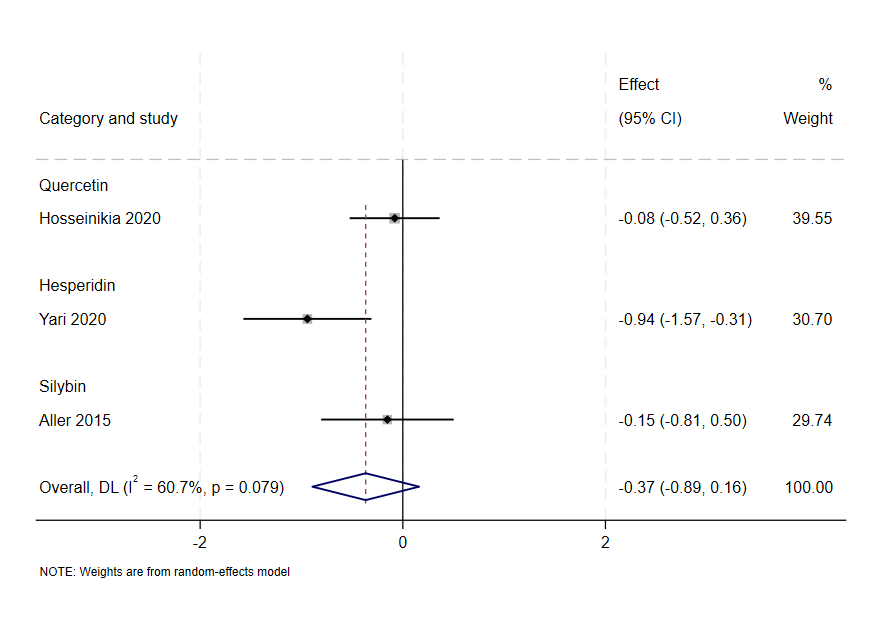


**Fig1:Effect of flavonoid on GGT**

2 Fibrosis score


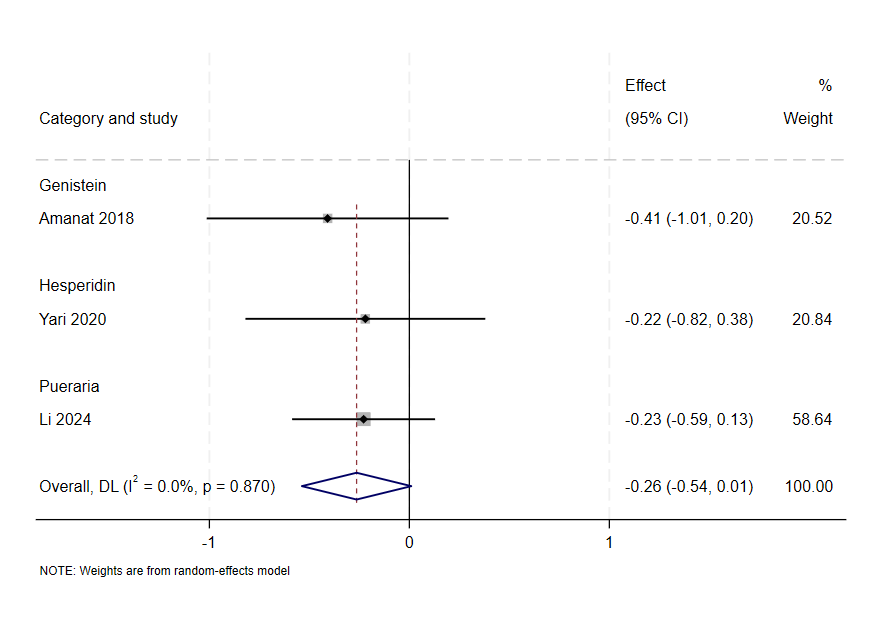


**Fig2:Effect of flavonoid on Fibrosis score**

3 Fatty Liver Index（FLI）


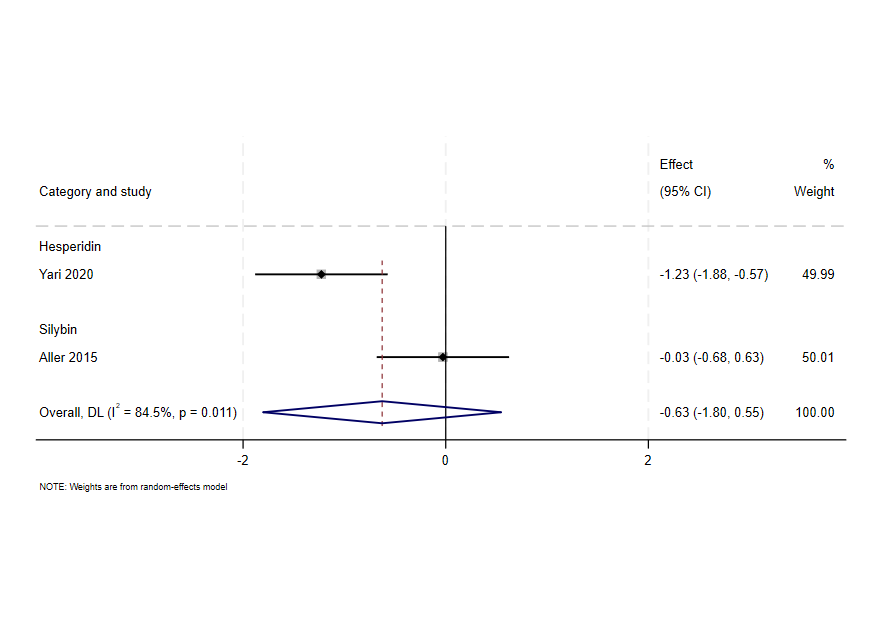


**Fig3 Effect of flavonoid on FLI**

4 Fibrosis-4（FIB-4）


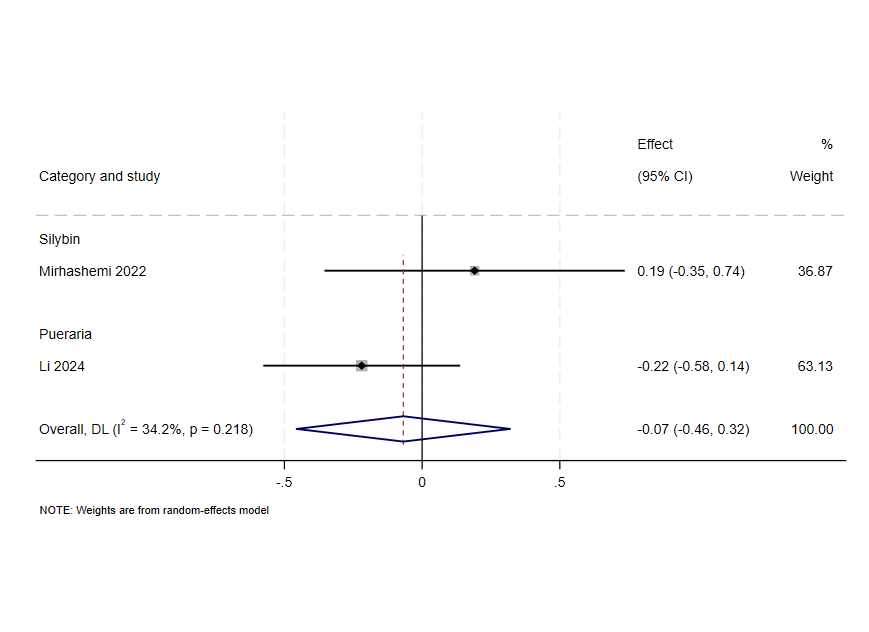


**Fig4 Effect of flavonoid on FIB-4**

5 NAFLD Fibrosis Score（NFS）


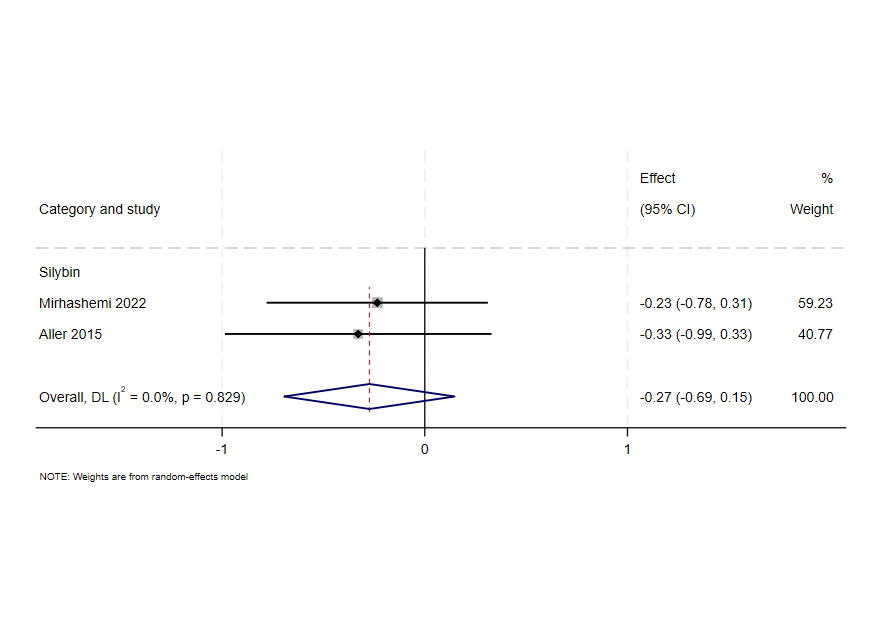


**Fig5 Effect of flavonoid on NFS**

6 Homeostatic Model Assessment of Insulin Resistance(HOMA-IR)


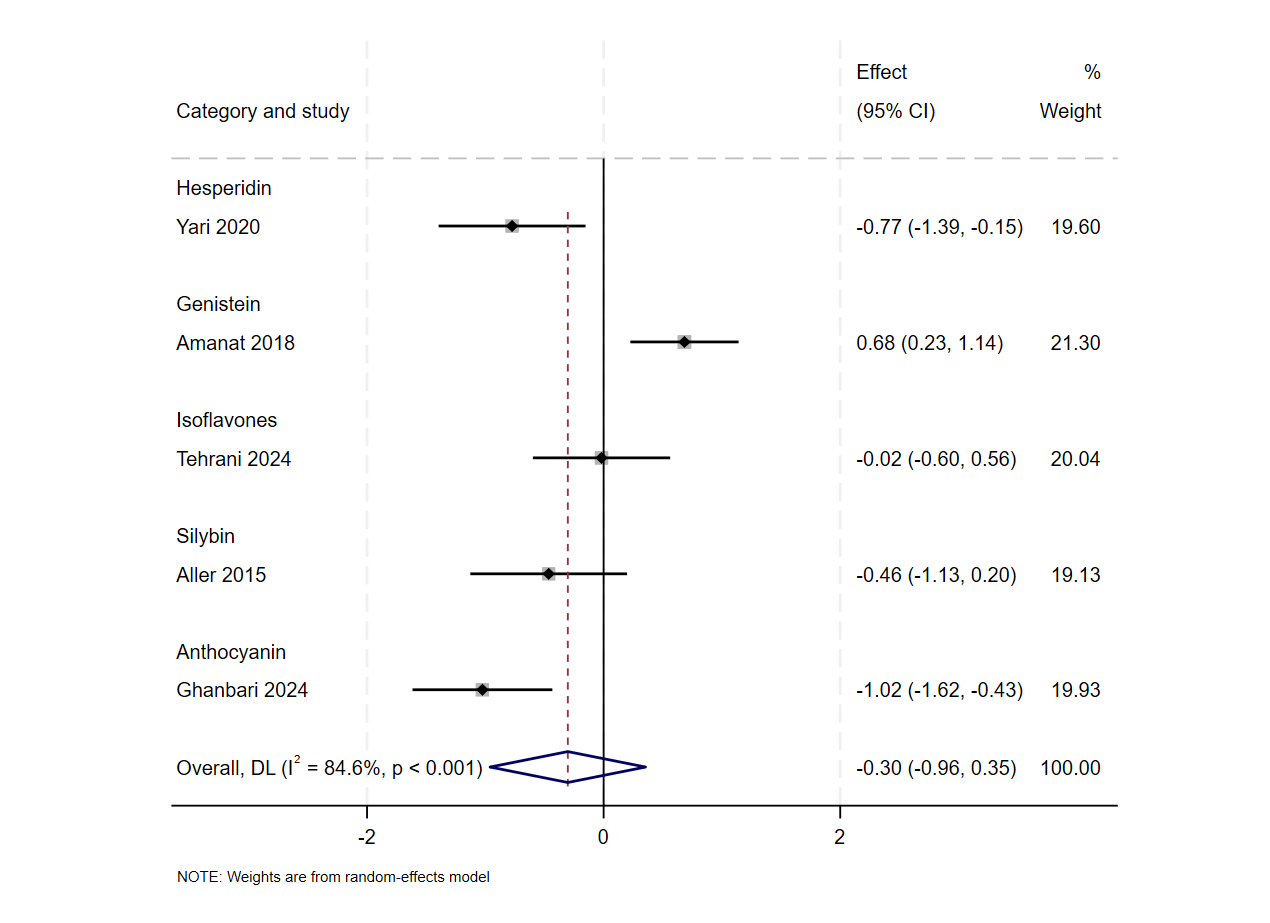


**Fig6 Effect of flavonoid on HOMA-IR**

7 Hip Circumference(HC)


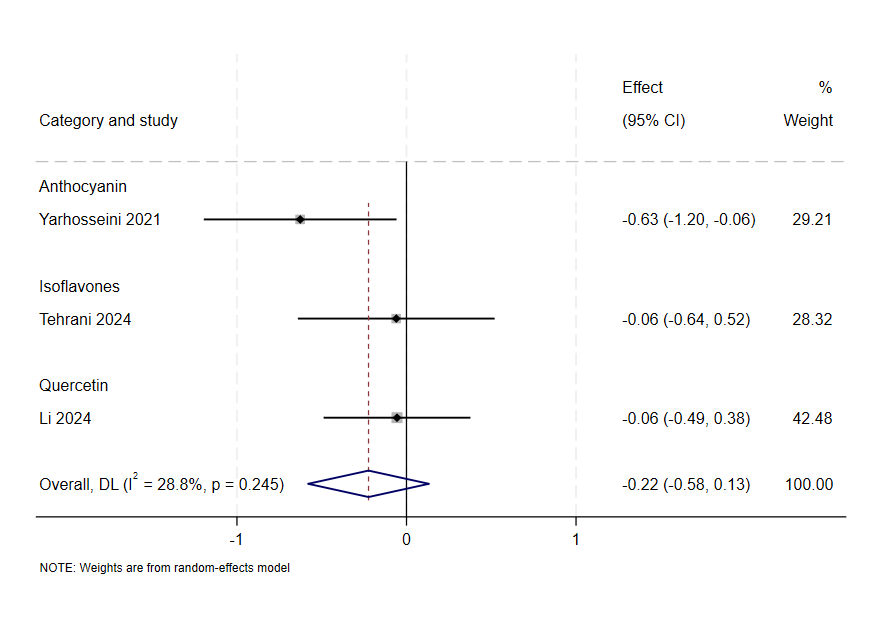


**Fig7 Effect of flavonoid on HC**

**8 Waist Circumference**(WC)

**
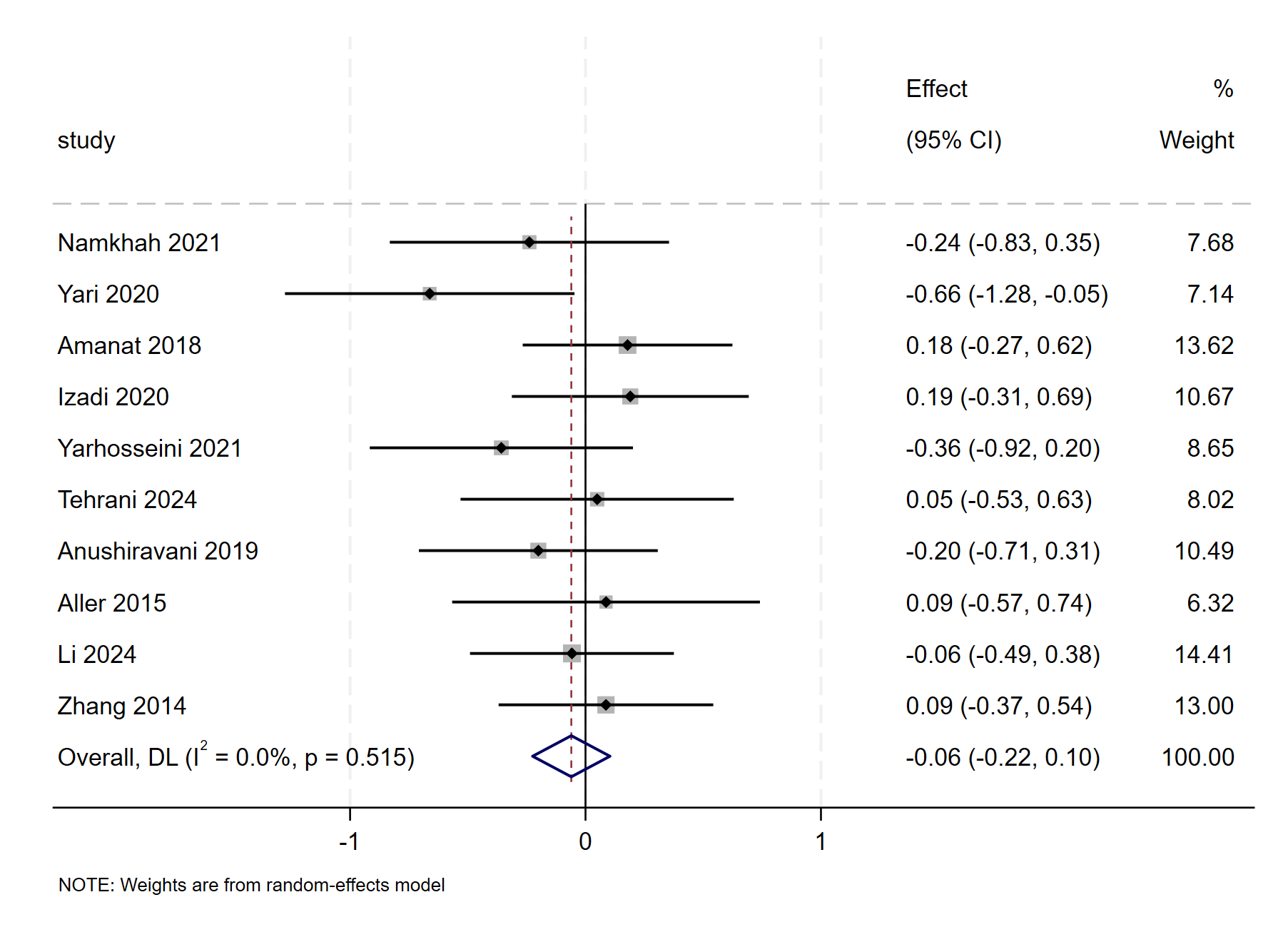
**

**Fig8 Effect of flavonoid on WC**

**9 waist-to-height ratio (WHtR)**

**
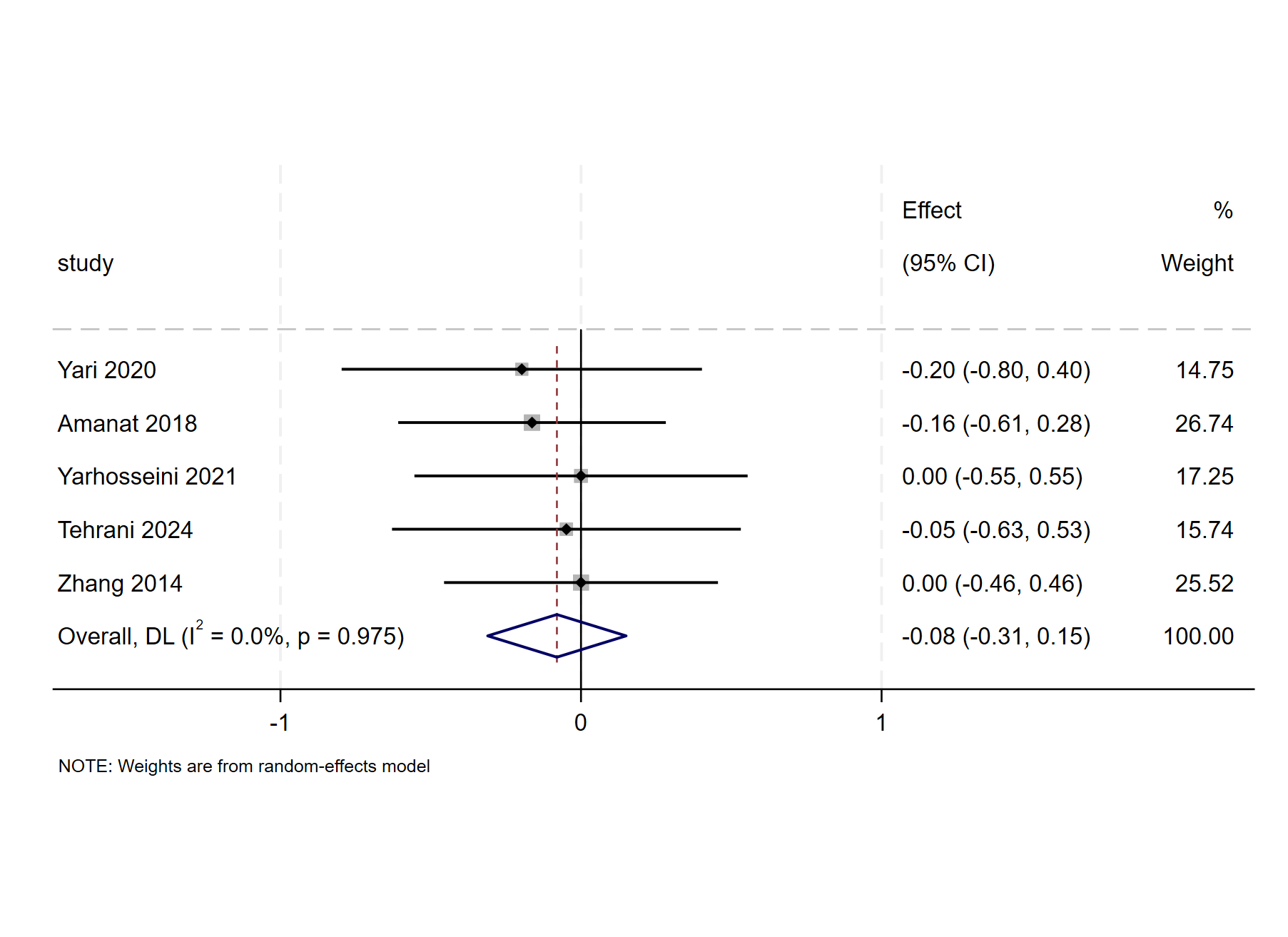
**

**Fig9 Effect of flavonoid on WHtR**

10 body weight (WT)


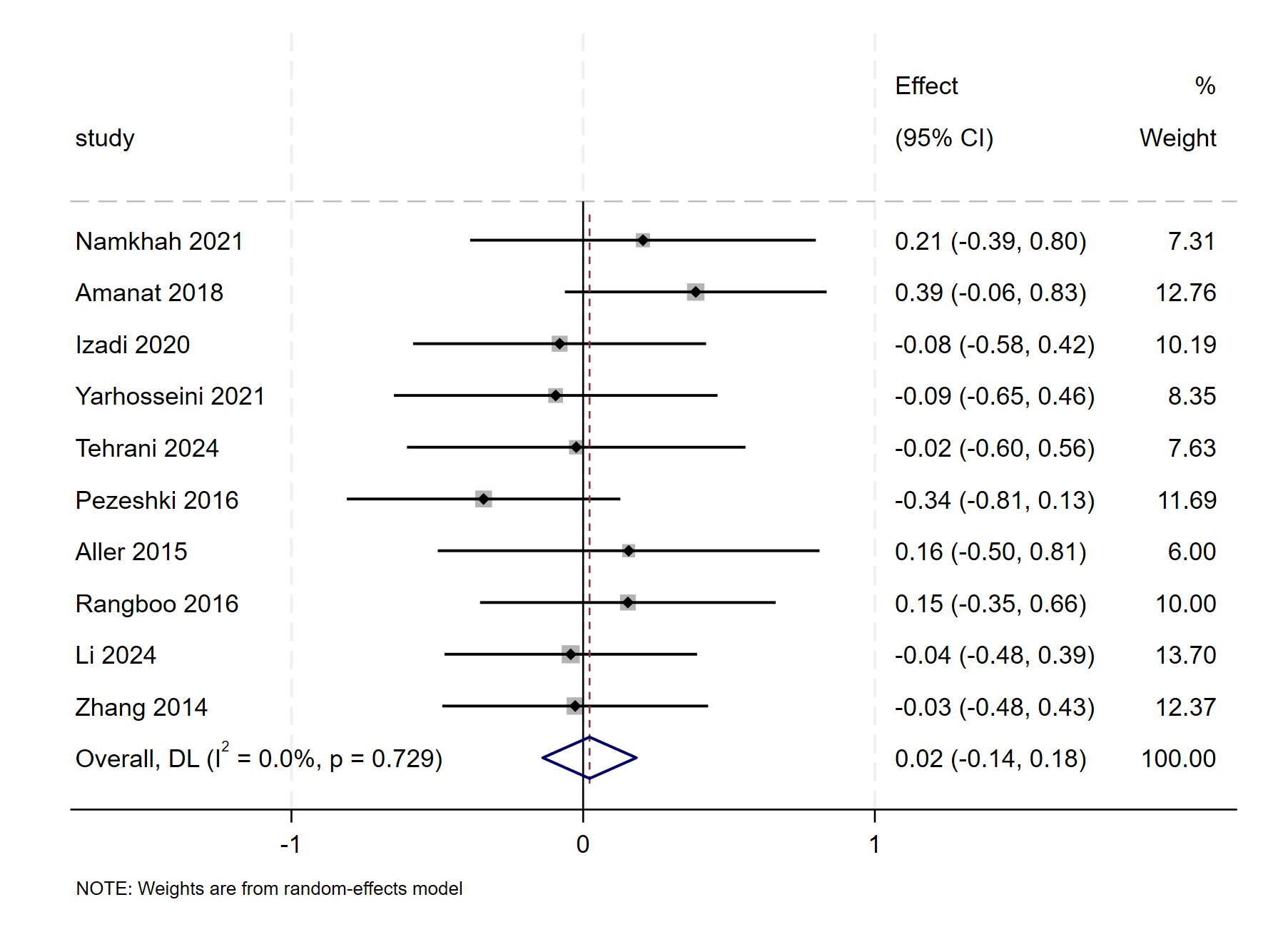


**Fig10 Effect of flavonoid on WT**

11 inflammatory markers


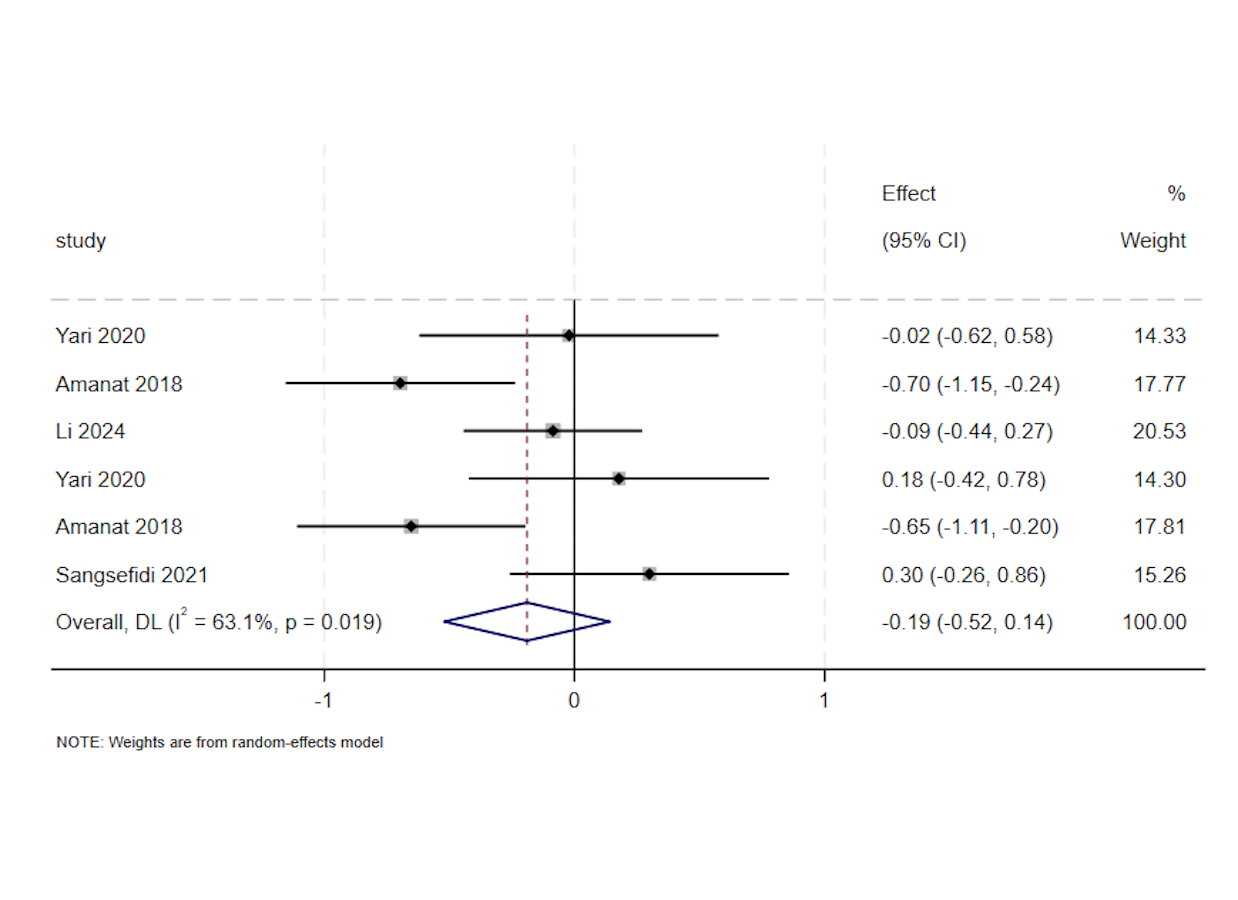


**Fig11 Effect of flavonoid on** inflammatory markers

12 low-density lipoprotein cholesterol (LDL-C)


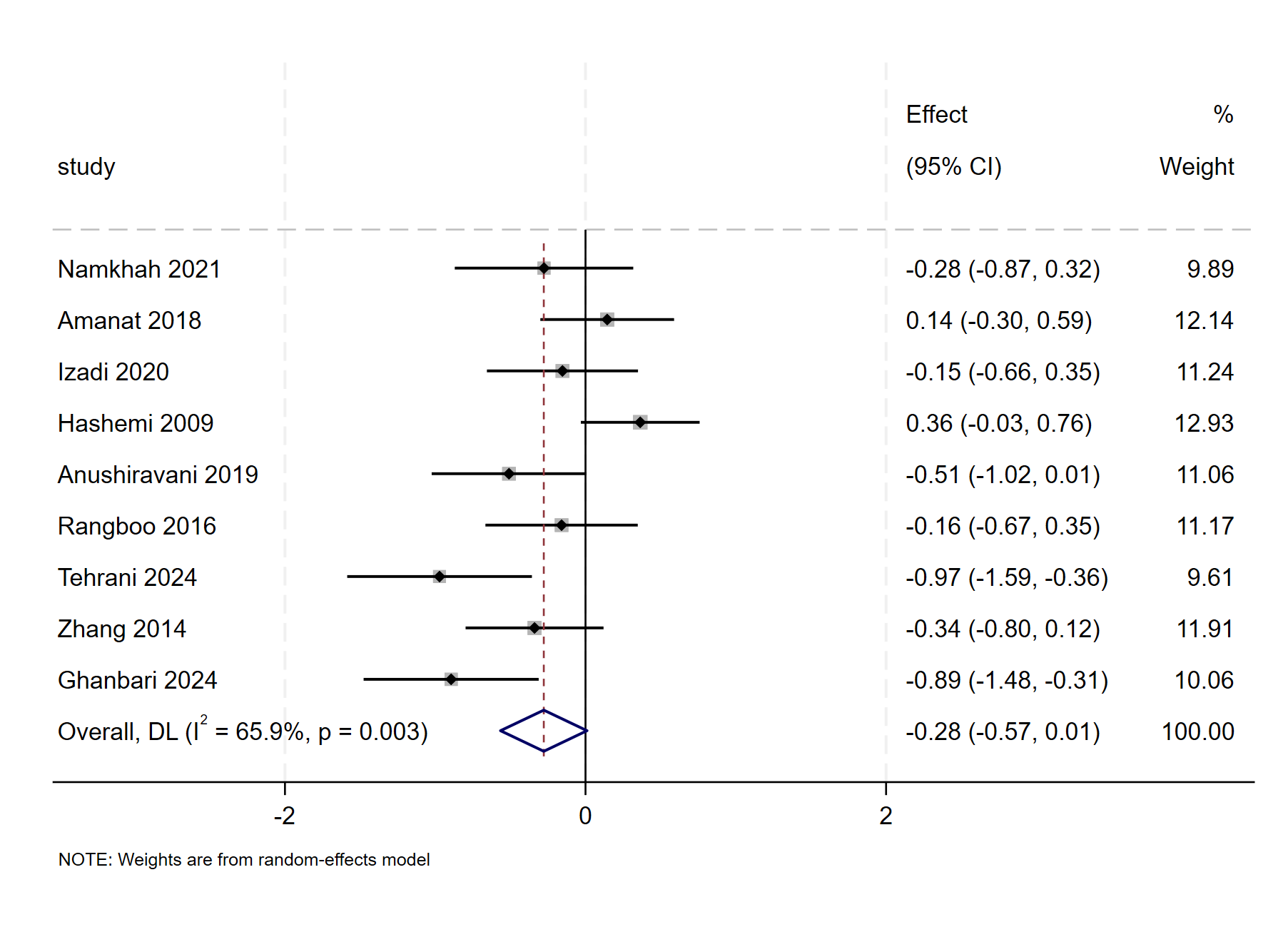


**Fig12 Effect of flavonoid on** LDL-C

13 High-Density Lipoprotein Cholesterol(HDL-C)


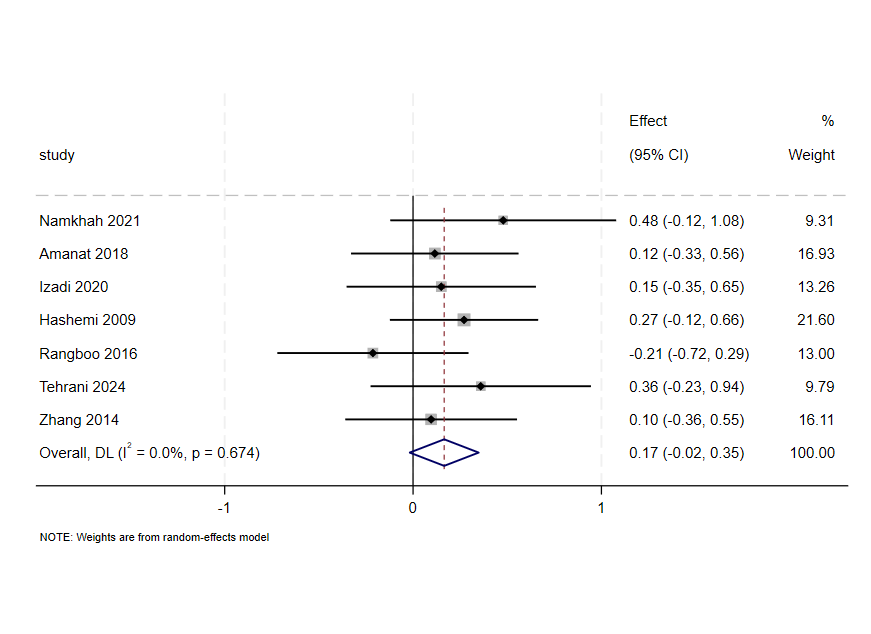


**Fig13 Effect of flavonoid on HDL-C**
